# Supplementary material for: TMBIM5 is the Ca2+/H+ antiporter of mammalian mitochondria
Source: EMBO Rep. 2022 Nov 2;23(12):e54978. doi: 10.15252/embr.202254978 (PMC9724676; doi:10.15252/embr.202254978)

Appendix

Table of contents

Appendix Table S1: Antibodies used in this study………………………………………………	page 1
Appendix Figure Legends………………………………………………………………………………	page 2
References……………………………………………………………………………………………………	page 4

Appendix Table S1: Antibodies used in this study

Antibody	*Dilution*	*Company*
Influenza-Hemagglutinin HA clone 7	1:1000	Sigma, #H9658
SLC24A6	1:1000	Abcam, #ab136975
LETM1	1:1000	Abnova, #H00003954
LETM1	1:1000	Santa Cruz Biotechnology, #sc-163013
LETM1 C-terminal region	1:1000	Aviva, Systems Biology, #OAAB12878
TMBIM5	1:1000	Abcam, #ab106754
TMBIM5	1:1000	Aviva systems biology, #OAAF06415
TMBIM5 serum (Oka et al, 2008)	1:200	Toshihiko Oka, Department of Life Science, Rikkyo University, Tokyo, Japan
OPA1	1:1000	BD Biosciences, #612606
SIRT3	1:1000	Cell Signaling Technology, #5490
DRP1	1:1000	Santa Cruz Biotechnology, #sc-271583
HSP60	1:1000	Santa Cruz Biotechnology, #sc-1052
TOM20	1:1000	Cell Signaling Technology, #42406
TOM40	1:1000	Santa Cruz Biotechnology, #sc-365467
OMA1	1:1000	Santa Cruz Biotechnology, #sc-515788
Prohibitin	1:1000	Abcam, #ab210082
β-Actin	1:1000	Invitrogen, #MA5-11869
PolyHistidine-Peroxidase	1:10000	Sigma Aldrich, #a7058
Goat-α-mouse	1:5000	Jackson ImmunoResearch, #115-035-003
Rabbit-α-goat	1:5000	Jackson ImmunoResearch, #305-035-003
Goat-α-rabbit	1:5000	Jackson ImmunoResearch, #111-035-144

Appendix Figure Legend

Appendix Figure S1 AP-MS experiments

(A) Proteins identified by AP-MS were scored for probability of interaction using SAINT score and fold change B using raw data and the CRAPome (left), proteins identified in Austin et al (red) and Sancak et al (blue). Both GFP & CRAPome controls of similar experimental setup were used for analysis to filter nonspecific interactors (right), proteins identified in Austin et al (red), Sancak et al (blue) and validated interaction partners found in both (green). **(B)** Venn diagram illustrating the number of proteins identified in mitochondrial single streptavidin AP (156 proteins) or whole cell tandem AP (98 proteins). While 31 proteins were found in common with both approaches, with 124 proteins being unique to the mitochondrial single streptavidin AP and 67 being unique to the whole cell tandem AP after nonspecific interactors removed from the Crapome. **(C)** SAINT and Fold change B (FC-B) for proteins identified in mitochondrial AP-MS experiment, interactors identified as high confidence interactors of LETM1 in both approaches shown in red and labelled where possible. **(D)** Native immunoblot of LETM1 (top panel) and TMBIM5 (lower panel) using three or two different antibodies respectively show that proteins can be found in protein complexes of the same size, LETM1 and TMBIM5 additionally reside in other protein complexes.

Appendix Figure S2 Ca^{2+} uptake/release in presence and absence of TMBIM5 and NCLX

(A) Western blot analysis of NCLX in total protein lysates from HEK293 WT cells and HEK293 NCLXKD (NCLXKD1 and 2) in the background of TMBIM5 KO1 cells. **(B)** Densitometric analysis of bands in (A) normalized to housekeeping gene (β-Actin). **(C)** Ca^{2+} uptake/release dynamics of permeabilized HEK293 cells are shown as extra-mitochondrial Ca^{2+} changes of fluorescence intensities of Calcium Green 5N (Ca^{2+}-5N) (0.24 μM), in presence of CGP37157 (2 μM). Ca^{2+}

(10 μM), RR (0.2 μM) and FCCP (2 μM) were added when indicated**.** **(D)** Quantification of Ca^{2+} release rates. Data are mean ±SD from three independent experiments (biological replicates) (t: 300-920 s)**.**

Appendix Figure S3 Quantification of Mitochondrial Ca^{2+} uptake rates

The decay rate was calculated as exponential decrease of the Ca^{2+} 5N Signals using the equation: Y= (Y0 - Plateau)*exp(-K*X) + Plateau, Rate of Decay/sec: K*Y0; X: Time (sec), Y: Starts at Y0 (Ca^{2+} peak) and decays with one phase down to Plateau; K: Rate constant equal to the reciprocal of the X axis units. Quantification of the Ca^{2+} uptake rates recorded in Fig 4A **(A)**, Fig 4C **(B)**, Fig 4G **(C)**, Fig 4K **(D)**, Fig 4I **(E)**, Fig EV5A **(F)** and Fig EV5G **(G).** Calculations were done using the GraphPad software and statistical analysis using Brown-Forsythe and Welch ANOVA. Data are mean ±SD, n=3 (biological replicates), significance p<0.05 for **(F)**.

Appendix Figure S4 TMBIM5^{D325R} is not important for the mitochondrial KHE activity

KOAc-induced swelling was measured in mitochondria from HEK293 TMBIM5WT (WT), TMBIM5KO cells stably expressing TMBIM5^{D325R} (D325R), or empty vector (EV) (left panel). Quantification of swelling amplitudes (right panel) Data are mean ±SD, n=3 (biological replicates).

Appendix Figure S5 western blot analysis of LETM1

(A) Western blot analysis of LETM1 in total protein lysates from HEK293 scr and LETM1KD. **(B)** Densitometric analysis of bands in (A) normalized to housekeeping gene (β-Actin). Data are mean ±SD n=3 (biological replicates), significance **p<0.01 using unpaired t test.

Appendix Figure S6 CRC in presence Tg and absence of CGP37157

Permeabilized HEK293 TMBIM5 WT (black trace) and TMBIM5KO (red trace) cells exposed **(A)** or not **(B)** to CsA were subjected to sequential Ca^{2+} bolus of 5 µM Ca^{2+} while NCLX was not inhibited and fluorescence intensity was recorded.

Appendix Figure S7 TMBIM5 optimization, induction and structure overview

(A) Nucleotide sequence of the codon optimized sequence of TMBIM5 **(B)** TMBIM5 expression and purification. Lane 1: page ruler prestained plus marker; lane 2: insoluble fraction of cell lysate after 2 hrs of 0.4 mM IPTG induction at 37 °C; lane 3: insoluble fraction of not induced cell lysate (negative control); lane 4: purified TMBIM5 protein. **(C)** Ribbon representation of the hTMBIM5 protein (Q9H3K2). PDB file retrieved from AlphaFhold at https://alphafold.ebi.ac.uk/entry/Q9H3K2. The unstructured loop containing the first 55 amino acids has been removed.

Appendix Figure S8 TMBIM5 affects LETM1 protein level through post-translational modifications

(A) qPCR analysis showing changes in gene expression in HeLa WT (ctrl) vs TMBIM5KO (KO) Data are mean ±SD, n=3 (biological replicates). **(B)** Left panel Western blot analysis of LETM1 in total protein lysates from HEK293 WT and TMBIM5KO treated with 10uM MG-132 or vehicle (DMSO) for 6 hours. Middle panel, Densitometric analysis of bands in MG-132 vs DMSO in the left panel normalized to housekeeping gene (TOM40). Right panel turnover rate of LETM1 in TMBIM5KO vs WT.

References

Oka T, Sayano T, Tamai S, Yokota S, Kato H, Fujii G, Mihara K (2008) Identification of a novel protein MICS1 that is involved in maintenance of mitochondrial morphology and apoptotic release of cytochrome c. *Molecular biology of the cell* **19:** 2597-2608

Appendix Figure S1

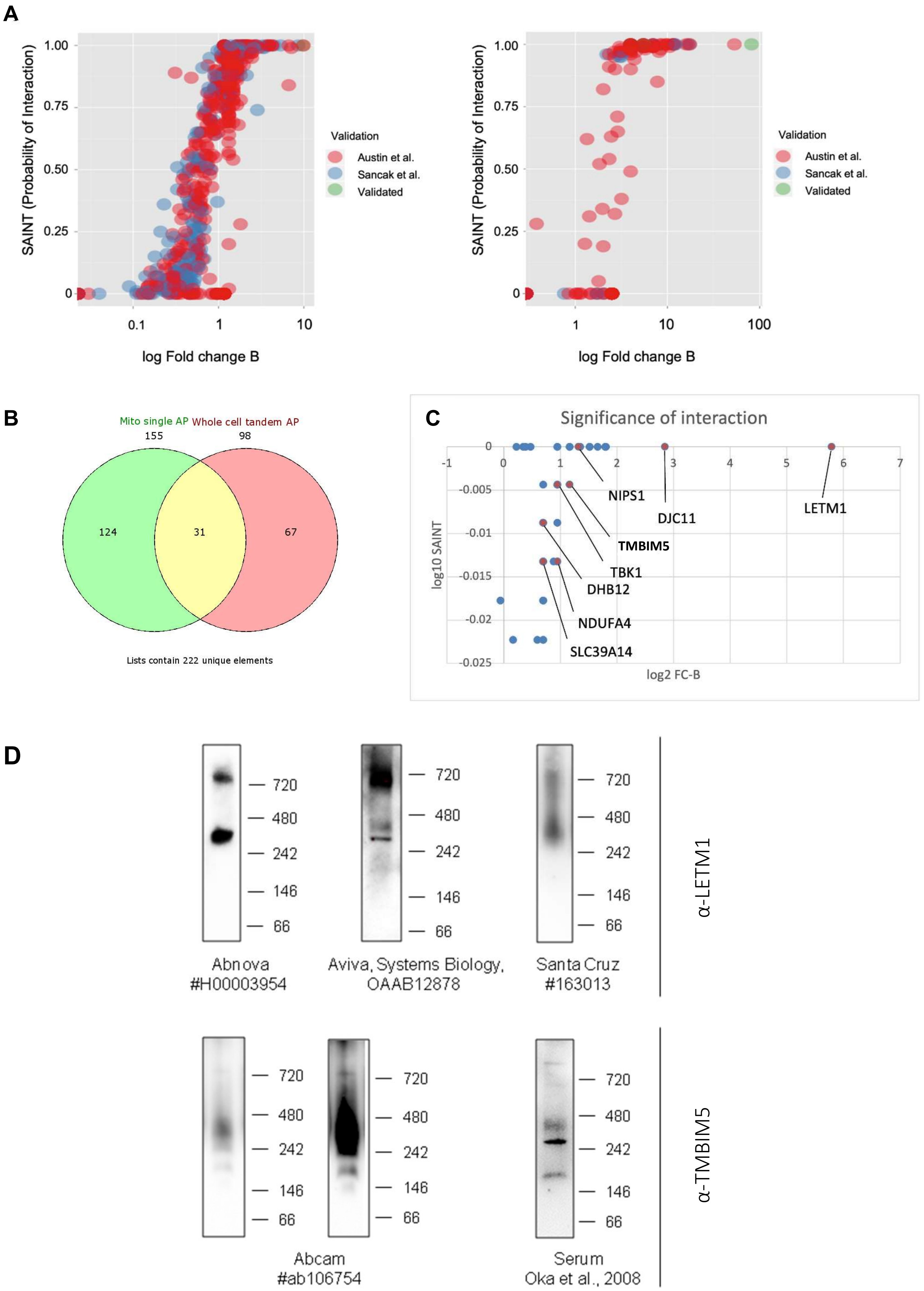

Appendix Figure S2

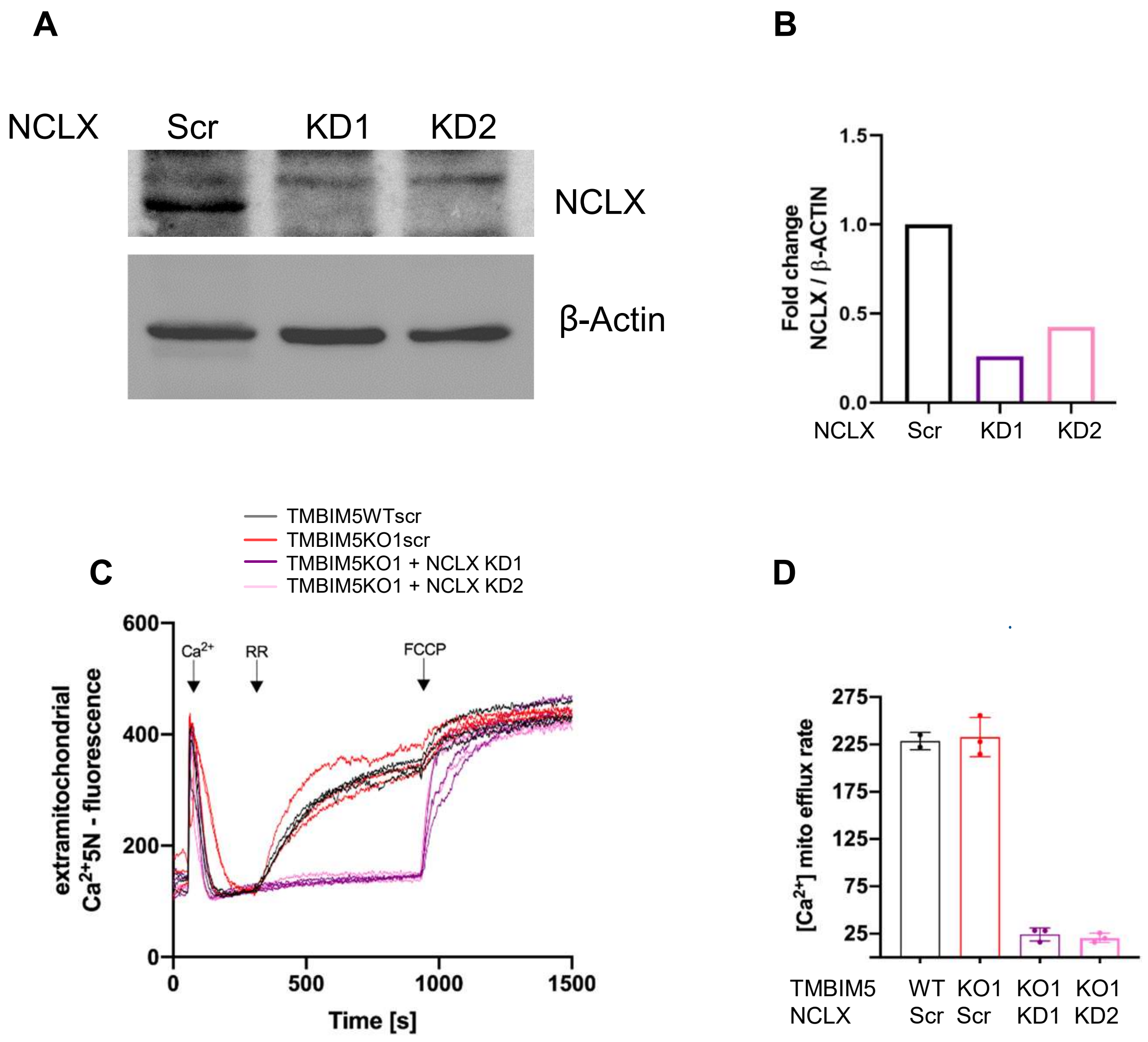

Appendix Figure S3

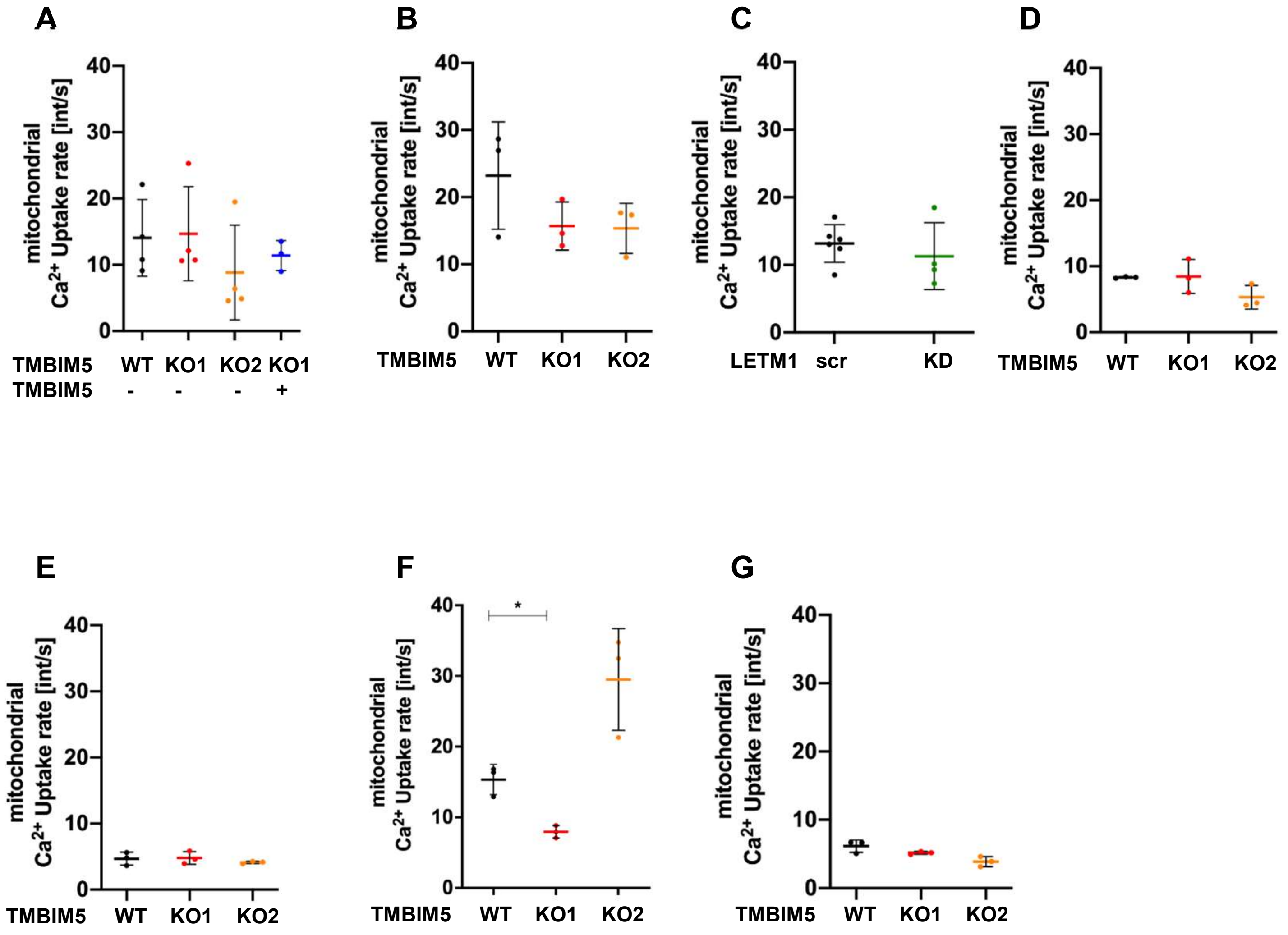

Appendix Figure S4

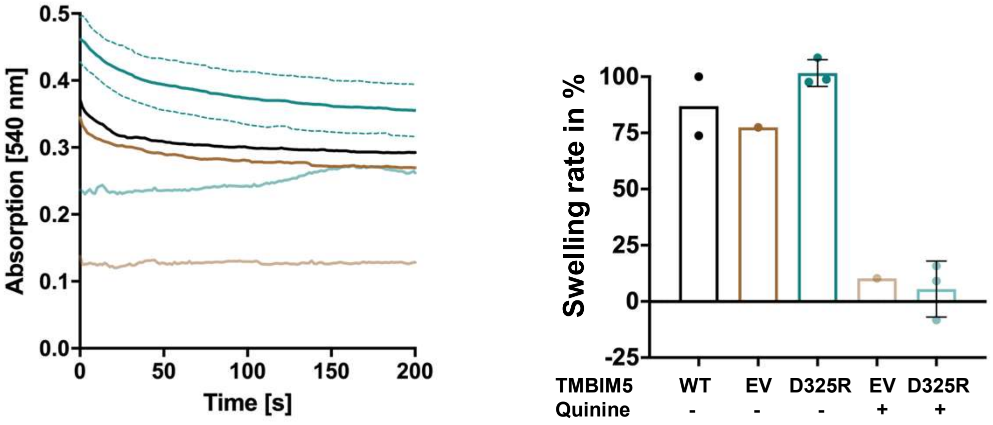

Appendix Figure S5

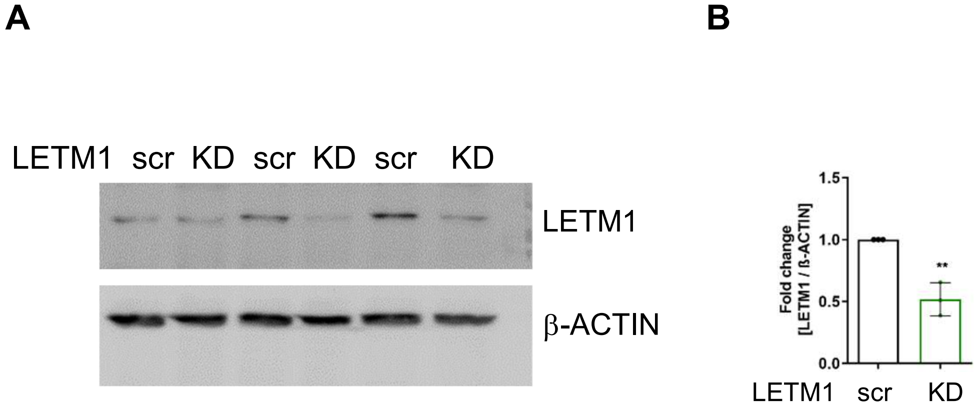

Appendix Figure S6

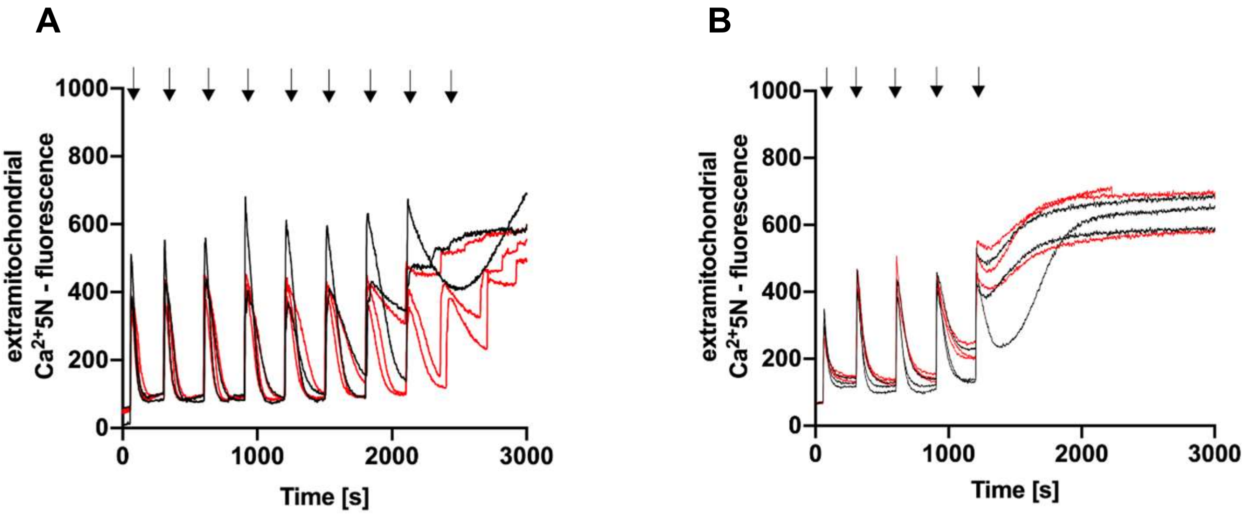

Appendix Figure S7

A

```
>Optimized_TMBIM5
ATGCTGGCGGCGCGTCTGGTGTGCCTGCGTACCCTGCCGAGCCGTGTTTTCCATCCGGCG
TTTACCAAAGCGAGCCCGGTTGTGAAGAACAGCATCACCAAGAACCAGTGGCTGCTGACC
CCGAGCCGTGAGTACGCGACCAAAACCCGTATCGGTATTCGTCGTGGCCGTACCGGTCAG
GAGCTGAAGGAAGCGGCGCTGGAGCCGAGCATGGAAAAGATCTTCAAAATTGACCAAATG
GGCCGTTGGTTCGTTGCGGGTGGCGCGGCGGTTGGTCTGGGTGCGCTGTGCTACTATGGT
CTGGGCCTGAGCAACGAGATCGGTGCGATTGAAAAGGCGGTGATCTGGCCGCAGTATGTT
AAAGATCGTATTCACAGCACCTACATGTATCTGGCGGGTAGCATTGGTCTGACCGCGCTG
AGCGCGATCGCGATTAGCCGTACCCCGGTTCTGATGAACTTCATGATGCGTGGCAGCTGG
GTGACCATCGGTGTTACCTTTGCGGCGATGGTGGGTGCGGGCATGCTGGTTCGTAGCATT
CCGTATGACCAAAGCCCGGGTCCGAAACATCTGGCGTGGCTGCTGCACAGCGGCGTGATG
GGTGCGGTGGTTGCGCCGCTGACCATTCTGGGTGGCCCGCTGCTGATTCGTGCGGCGTGG
TATACCGCGGGTATTGTGGGTGGCCTGAGCACCGTTGCGATGTGCGCGCCGAGCGAGAAA
TTCCTGAACATGGGTGCGCCGCTGGGTGTTGGCCTGGGTCTGGTGTTCGTTAGCAGCCTG
GGCAGCATGTTTCTGCCGCCGACCACCGTGGCGGGTGCGACCCTGTACAGCGTTGCGATG
TATGGTGGCCTGGTGCTGTTCAGCATGTTTCTGCTGTACGATACCCAGAAAGTGATTAAA
CGTGCGGAAGTTAGCCCGATGTACGGTGTGCAAAAATATGACCCGATCAACAGCATGCTG
AGCATTTATATGGATACCCTGAACATTTTTATGCGTGTGGCGACCATGCTGGCGACCGGC
GGCAACCGTAAGAAA
```

B

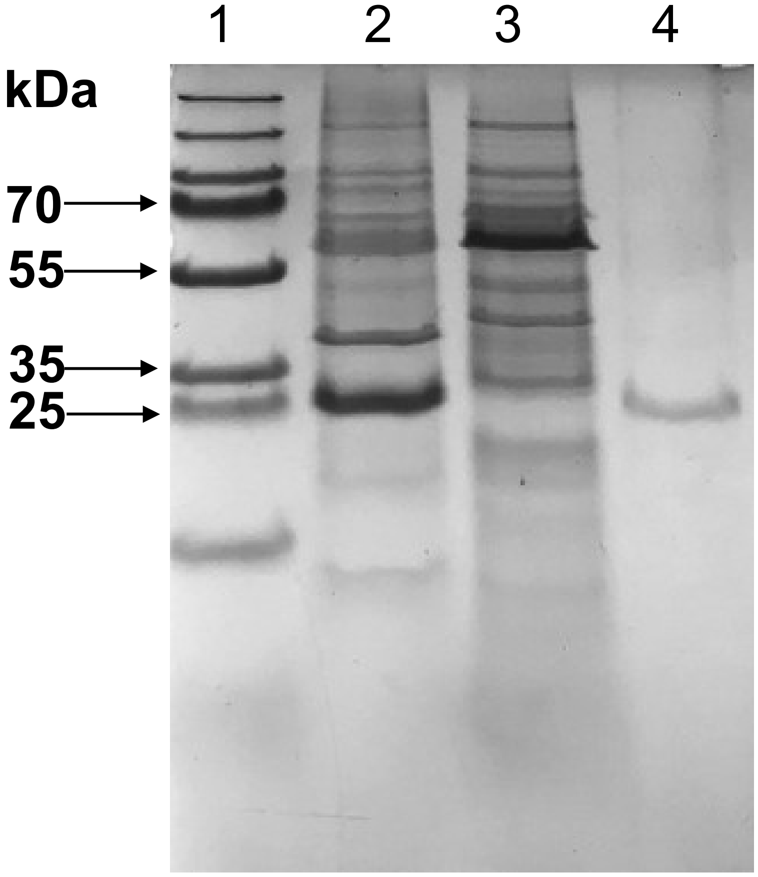

C

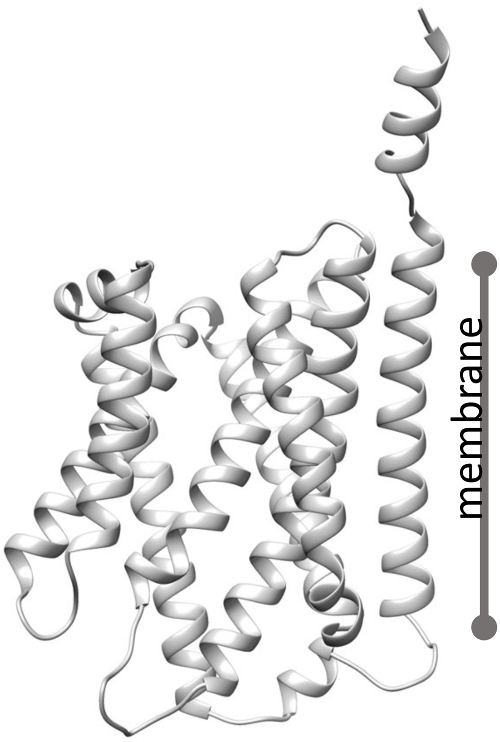

Appendix Figure S8

A

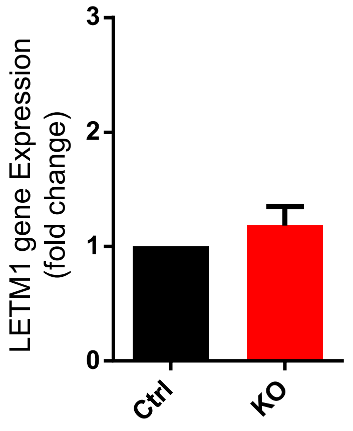

B

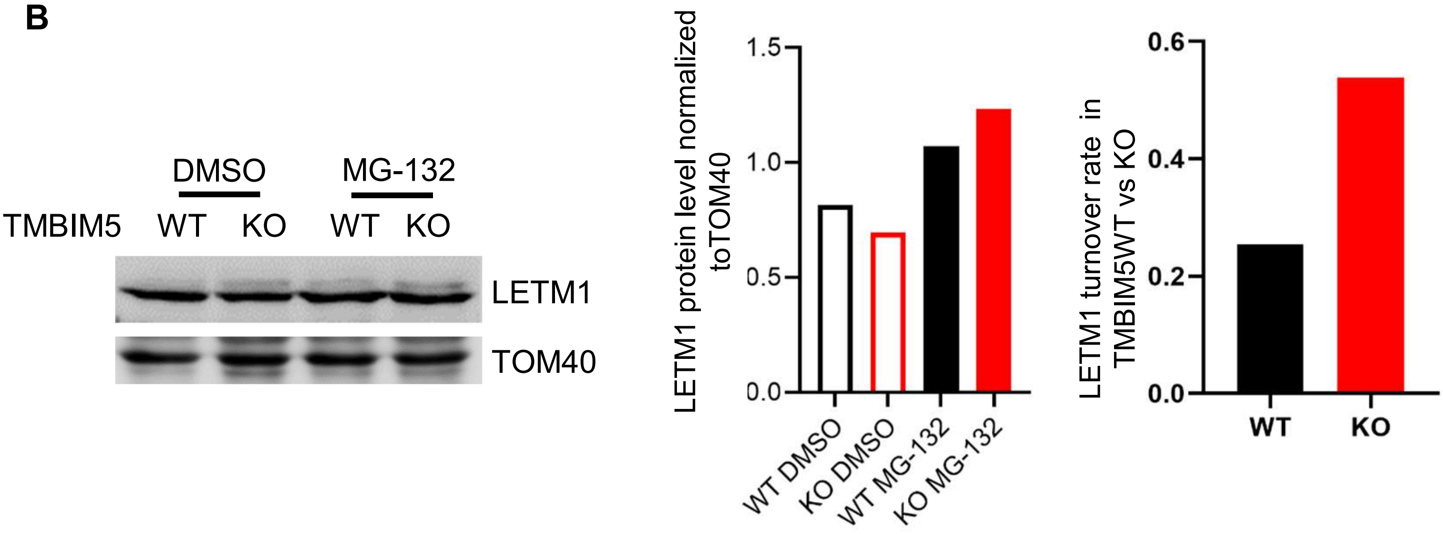

Supplement: Supplementary file 1 — Appendix [file EMBR-23-e54978-s006.pdf]
