# Supplementary material for: TMBIM5 is the Ca2+/H+ antiporter of mammalian mitochondria
Source: EMBO Rep. 2022 Nov 2;23(12):e54978. doi: 10.15252/embr.202254978 (PMC9724676; doi:10.15252/embr.202254978)
Supplement: Supplementary file 2 — Expanded View Figures PDF [file EMBR-23-e54978-s005.pdf]

Expanded View Figures

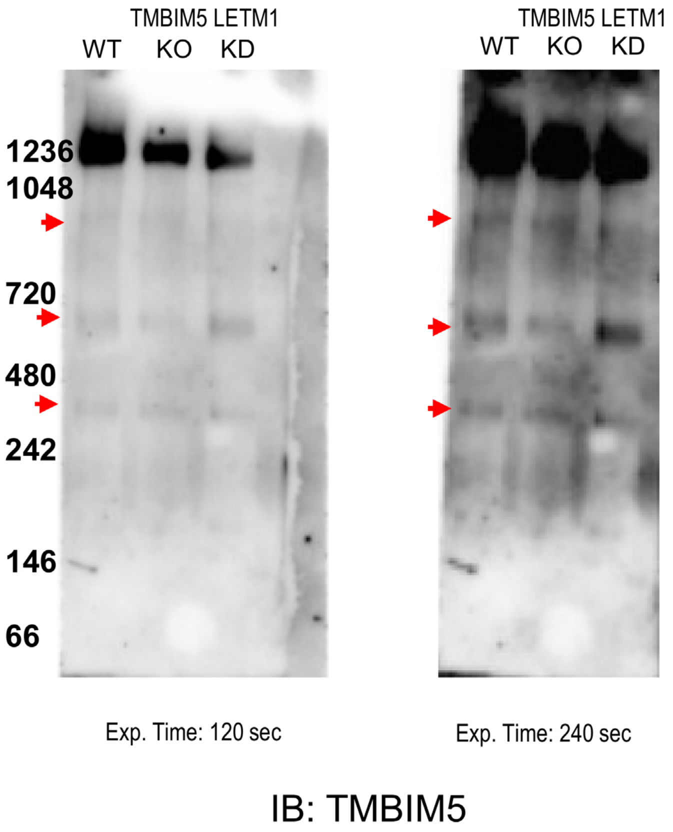

Figure EV1. TMBIM5 and LETM1 are present in protein complexes of the same molecular weight.

Immunoblotting analysis of blue native PAGE of isolated mitochondria from HeLa WT, TMBIM5KO, or LETM1KD cells, using the indicated antibody, arrows indicate TMBIM5 complexes.

 © 2022 The Authors

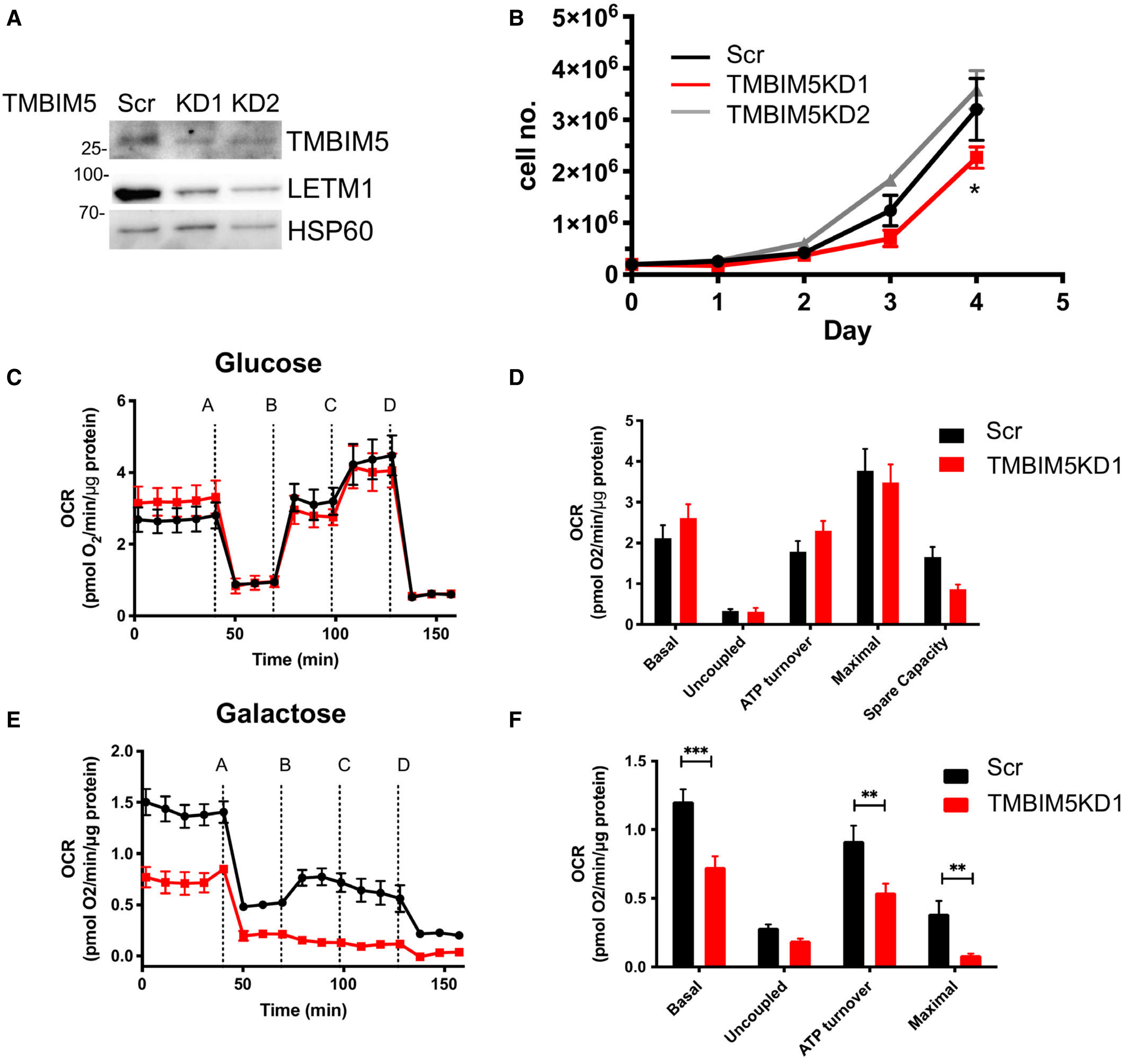

Figure EV2. TMBIM5KD decreases LETM1 and mitochondrial bioenergetics.

A Western blot analysis of LETM1 and TMBIM5 in HEK293 TMBIM5WT cells with a scramble shRNA and two different TMBIM5 knockdowns, HSP60 served as a loading control.

B Proliferation curve of TMBIM5WT scramble controls (scr) compared with TMBIM5KD cells (KD) over 4 days. Data are means ± SEM (scr, KD2 $n = 3$, KD1 $n = 5$) (biological replicates), at 96 h statistical analysis using an unpaired student's t-test (*$P < 0.05$).

C–F Cellular bioenergetics of TMBIM5KD cells in various nutrient conditions. Oxygen consumption rate of WT cells with a scrambled control (WT) and TMBIM5KD cells (KD) grown in (C) 25 mM glucose, (E) 10 mM galactose for 24 h before measurement. Data are representative of at least three independent experiments (biological replicates). Shown are the mean data of triplicate measurements ± SEM. Inhibitors as indicated: (A) oligomycin (0.5 µM), (B, C) FCCP (0.2 µM each), (D) antimycin A/rotenone (0.5 µM). (D, F) Bar charts of XF experiment traces (C, E), data are means of multiple time points after experiment start or drug addition of at least three independent experiments ± SEM (biological replicates). Statistical analysis using an unpaired student's t-test (**$P < 0.01$, ***$P < 0.001$).

Source data are available online for this figure.

© 2022 The Authors

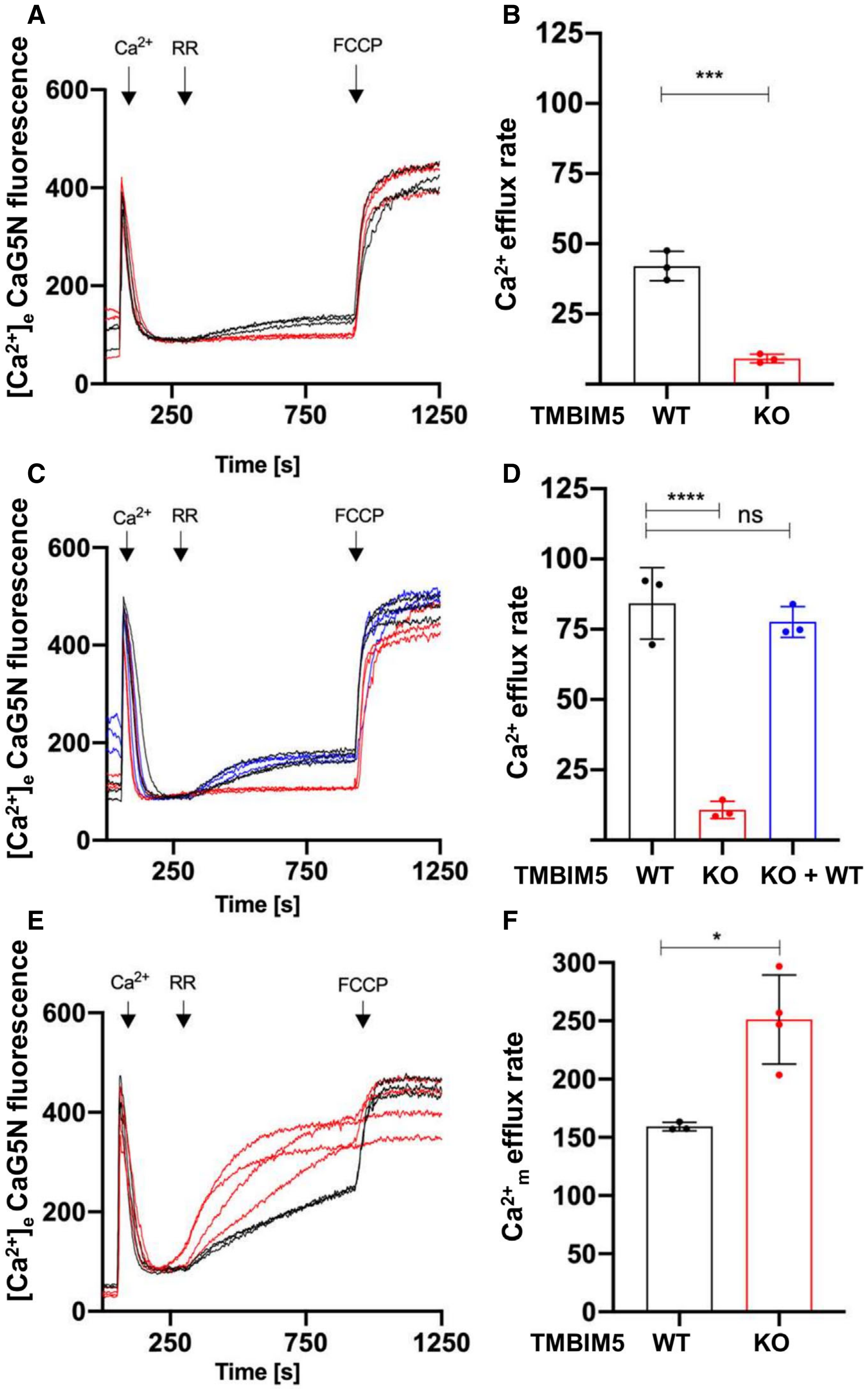

Figure EV3. TMBIM5 mitochondrial Na^+-dependent Ca^{2+} release in HeLa cells.

A–D Ca^{2+} uptake release assays were conducted in permeabilized HeLa TMBIM5WT or TMBIM5KO cells or TMBIM5KO cells expressing pcDNA3.1+TMBIM5 in presence of CGP37157 (2 μM) as described in Fig 4A–D applying a 10 μM (A) or 20 μM (C) Ca^{2+} pulse. In (C) Ca^{2+} uptake release was recorded in permeabilized HeLa TMBIM5WT or TMBIM5KO cells or TMBIM5KO re-expressing TMBIM5 cells.

E, F Same experimental setting as in (C) but in presence of Tg (1 μM). Quantification of ≥ 3 independent experiments (biological replicates), data are the mean ± SD with an unpaired student's test (B, F) and one-way ANOVA with Bonferroni correction (D), (* $P < 0.05$, *** $P < 0.001$, **** $P < 0.0001$, ns, not significant).

Source data are available online for this figure.

 © 2022 The Authors

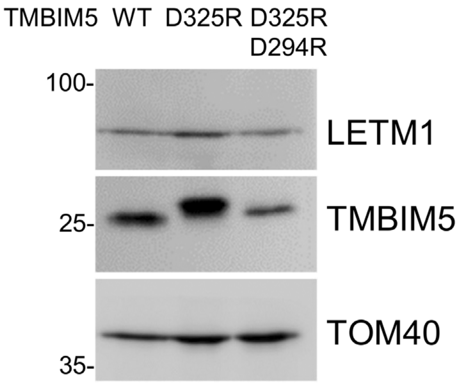

Figure EV4. TMBIM5 protein levels are not decreased by TMBIM5^{D325R}.

Immunoblots of Isolated mitochondria from HEK293 TMBIM5WT and TMBIM5KO expressing TMBIM5^{D325R} or double mutant TMBIM5$^{D325R/D294R}$ using the indicated antibodies, TOM40 served as mitochondrial loading control.

Source data are available online for this figure.

Figure EV5. TMBIM5KO induces PTP opening under Ca^{2+} overload.

A–H Ca^{2+} uptake/release dynamics in presence of CGP37157 (2 μM) and Tg (1 μM) were monitored as in Fig 5. Ca^{2+} (10 μM), RR (0.2 μM), and FCCP (2 μM) were added when indicated. Membrane potential was recorded as the change in fluorescence intensities of TMRM (330 nM) (C, D) corresponding to the measurement of Ca^{2+} fluxes in (A, B). CsA was added 2 min before measurements in (E, F dotted lines). Quantification of Ca^{2+} release rates from three independent experiments (biological replicates) are shown as means ± SD (t: 300–920 s) and statistical analysis: One-Way ANOVA with Bonferroni correction (*$P < 0.05$, **$P < 0.01$, ***$P < 0.001$, ****$P < 0.0001$). Quantification of TMRM performed with an unpaired two-sided t-test (Welsh correction), *$P < 0.05$. (E, F) Calcium retention capacity (CRC) assays showing that the absence of TMBIM5 supersensitizes mitochondria to Ca^{2+}-induced PTP opening by Tg. See also Appendix Fig S6A and B for CRCs in absence of Tg. Permeabilized HEK293 TMBIM5WT (E) and TMBIM5KO1 (F) cells exposed or not to CsA were subjected to sequential Ca^{2+} bolus of 5 μM Ca^{2+}, and fluorescence intensity was recorded.

Source data are available online for this figure.

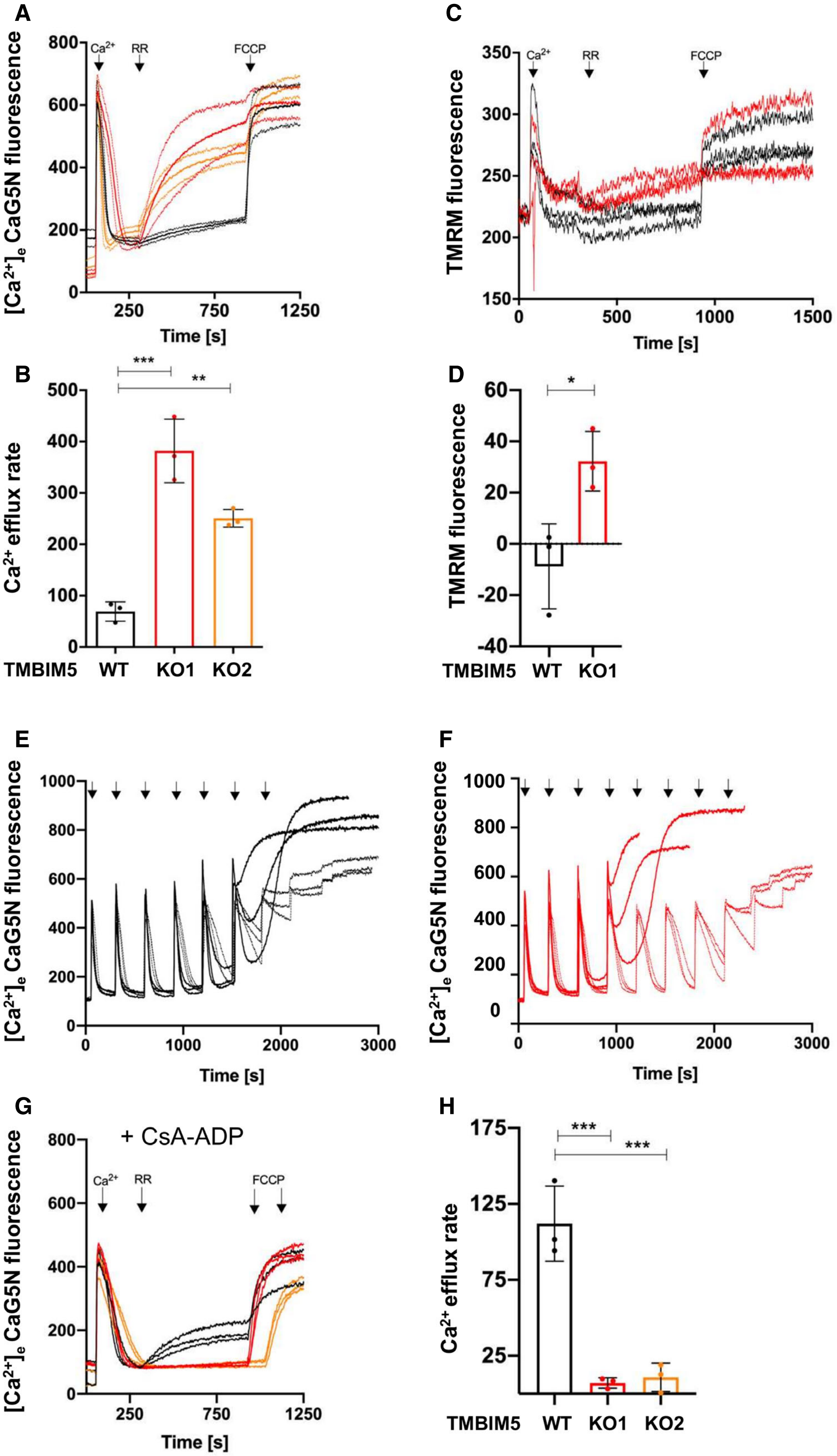

Figure EV5.

 © 2022 The Authors
