# Supplementary material for: TMBIM5 is the Ca2+/H+ antiporter of mammalian mitochondria
Source: EMBO Rep. 2022 Nov 2;23(12):e54978. doi: 10.15252/embr.202254978 (PMC9724676; doi:10.15252/embr.202254978)
Supplement: Supplementary file 4 — Source Data for Expanded View and Appendix [file EMBR-23-e54978-s013.zip › Appendix Figure S1D_source data.pptx]

## Slide 1
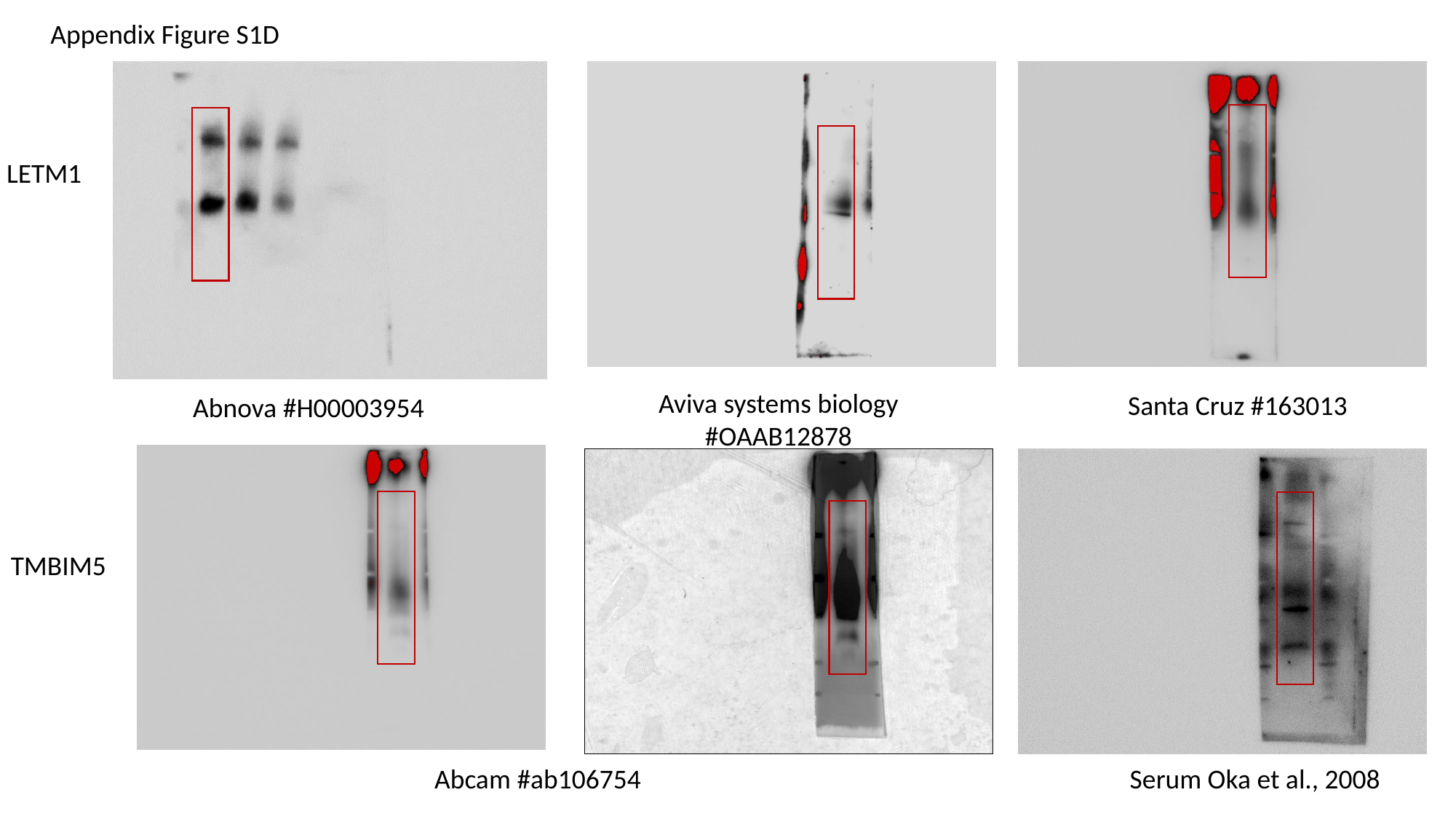

Appendix Figure S1D
LETM1
Aviva systems biology #OAAB12878
Santa Cruz #163013
Abnova #H00003954
TMBIM5
Serum Oka et al., 2008
Abcam #ab106754
