# Supplementary figures and images for: TMBIM5 is the Ca2+/H+ antiporter of mammalian mitochondria
Source: EMBO Rep. 2022 Nov 2;23(12):e54978. doi: 10.15252/embr.202254978 (PMC9724676; doi:10.15252/embr.202254978)

## Slide 1
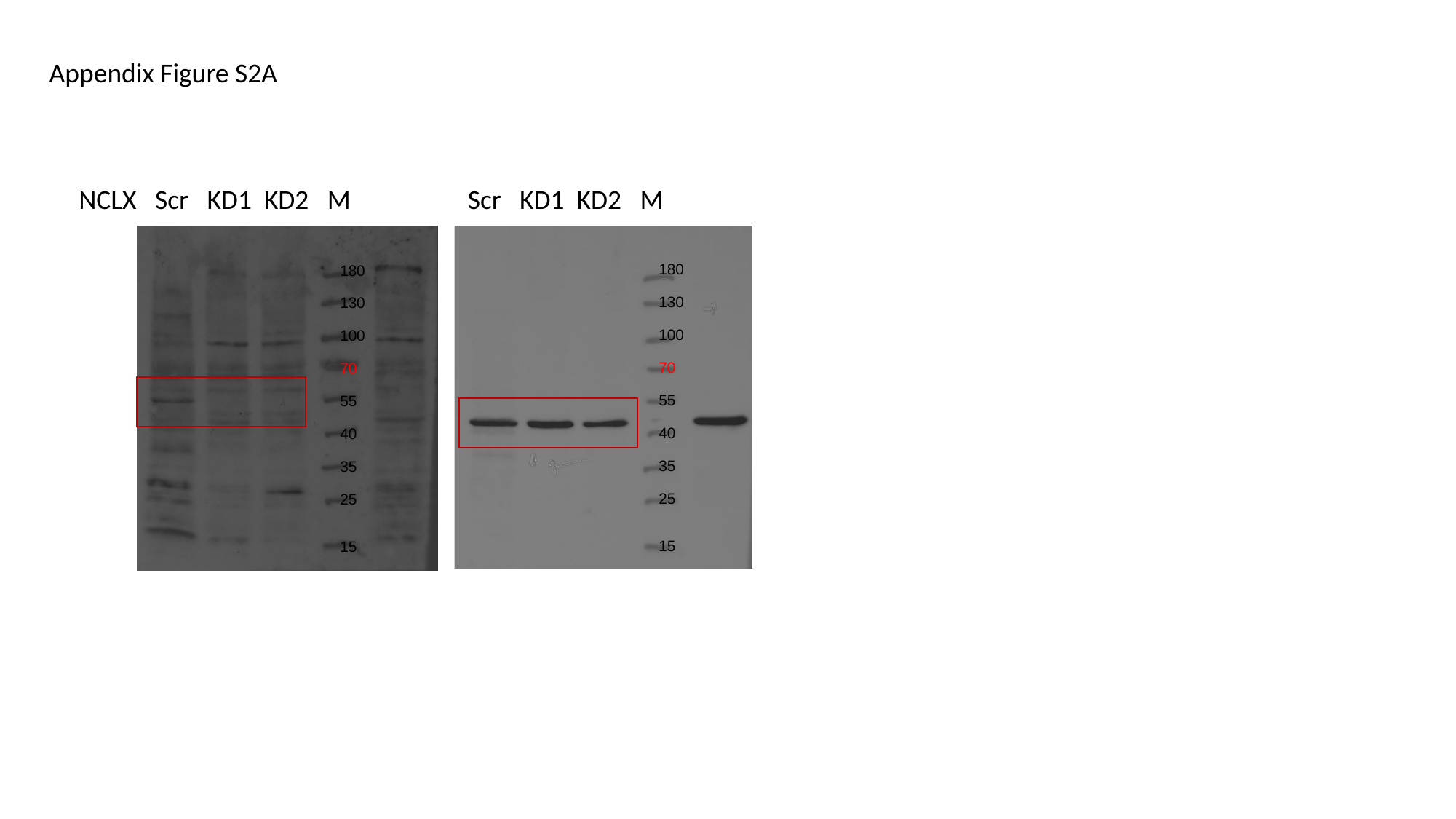

Appendix Figure S2A
NCLX Scr KD1 KD2 M Scr KD1 KD2 M
180
130
100
70
55
40
35
25
15
180
130
100
70
55
40
35
25
15

Supplement: Supplementary file 4 — Source Data for Expanded View and Appendix [file EMBR-23-e54978-s013.zip › Appendix Figure S2A_source data.pptx]

## Slide 1
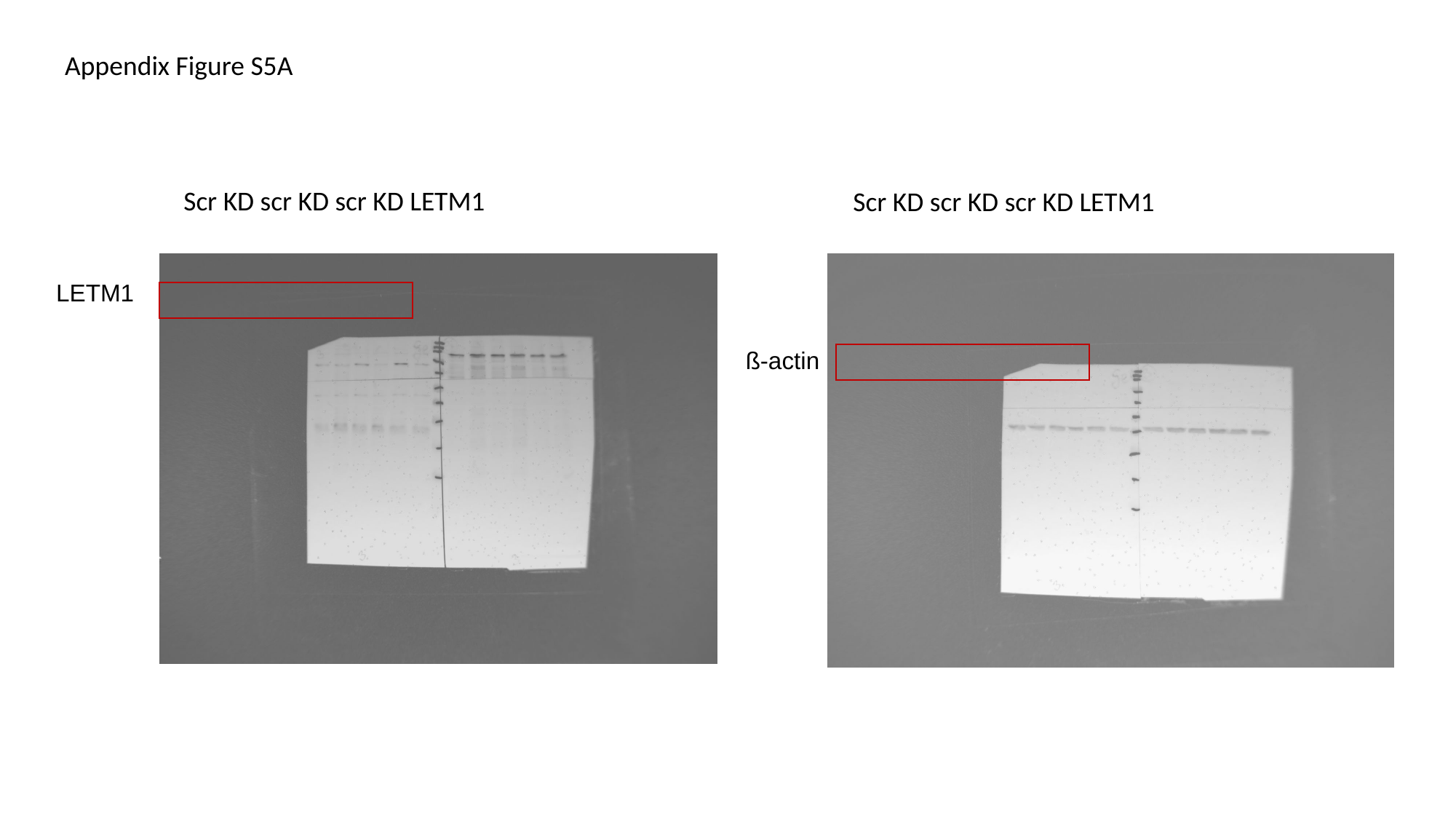

Appendix Figure S5A
Scr KD scr KD scr KD LETM1
Scr KD scr KD scr KD LETM1
LETM1
ß-actin

Supplement: Supplementary file 4 — Source Data for Expanded View and Appendix [file EMBR-23-e54978-s013.zip › Appendix Figure S5A_source data.pptx]

## Slide 1
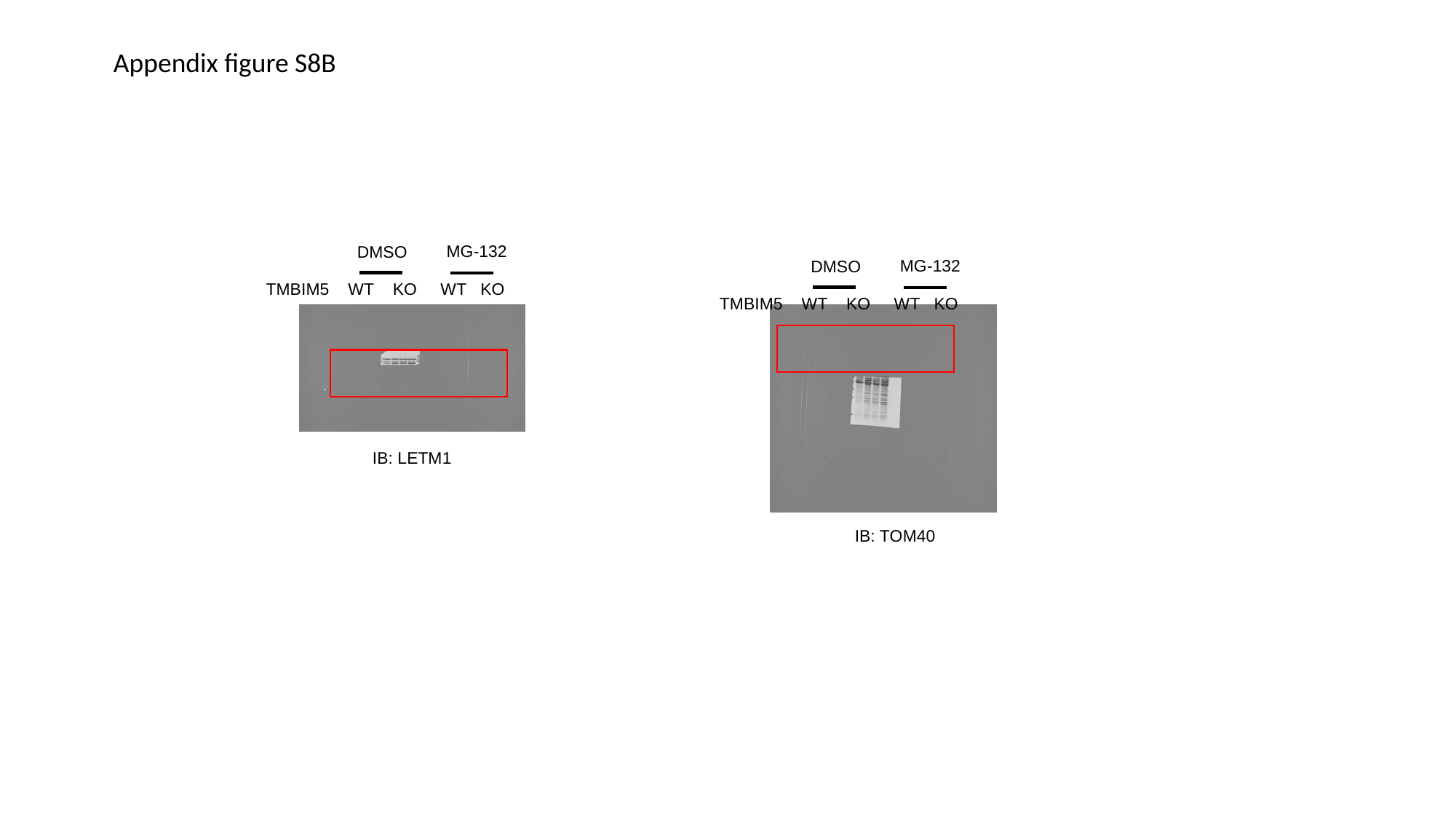

Appendix figure S8B
MG-132
DMSO
MG-132
DMSO
TMBIM5 WT KO WT KO
TMBIM5 WT KO WT KO
IB: LETM1
IB: TOM40

Supplement: Supplementary file 4 — Source Data for Expanded View and Appendix [file EMBR-23-e54978-s013.zip › Appendix Figure S8B source data.pptx]

## Slide 1
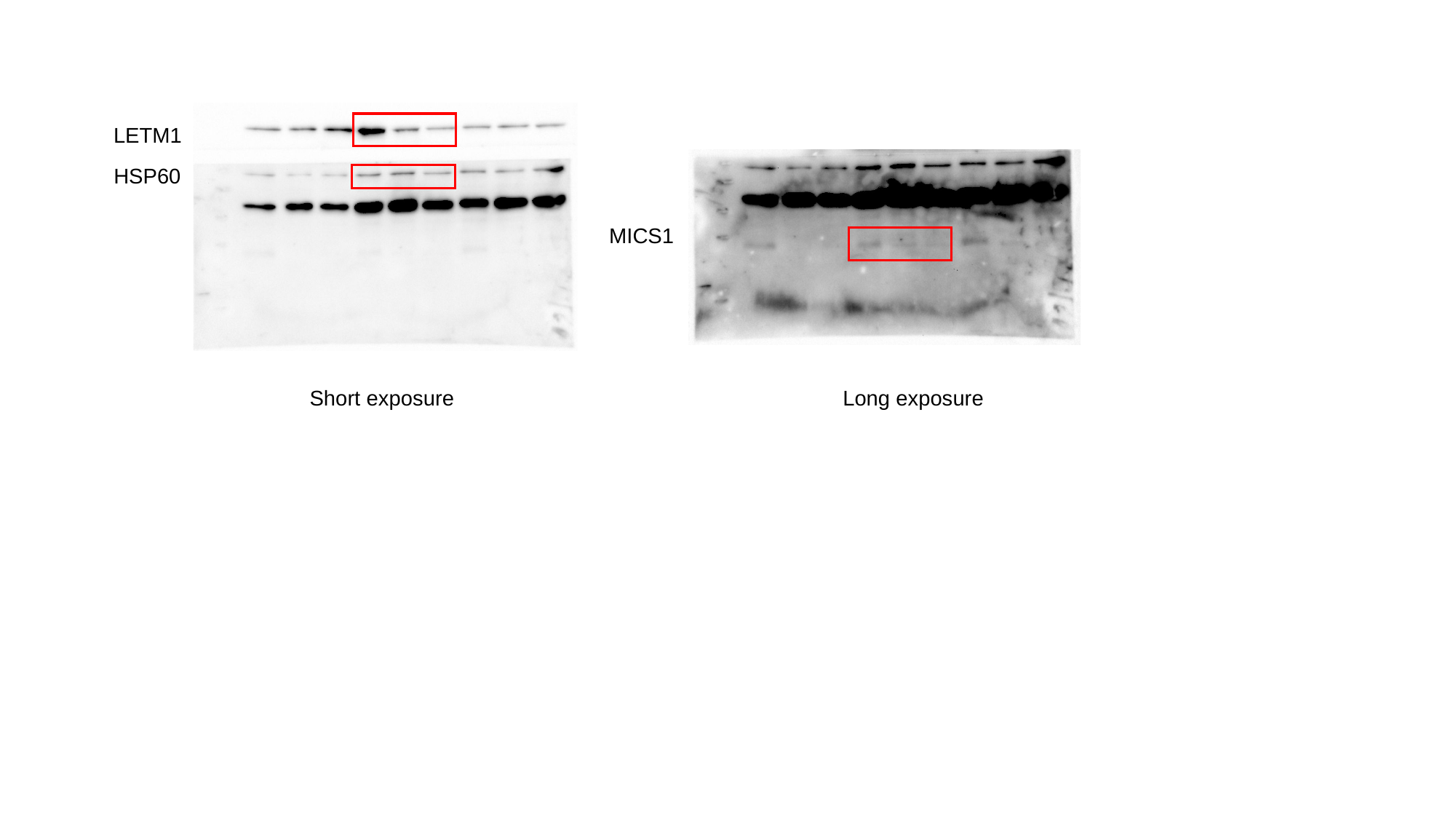

LETM1
HSP60
MICS1
Long exposure
Short exposure

Supplement: Supplementary file 4 — Source Data for Expanded View and Appendix [file EMBR-23-e54978-s013.zip › Figure EV2A source data.pptx]

## Slide 1
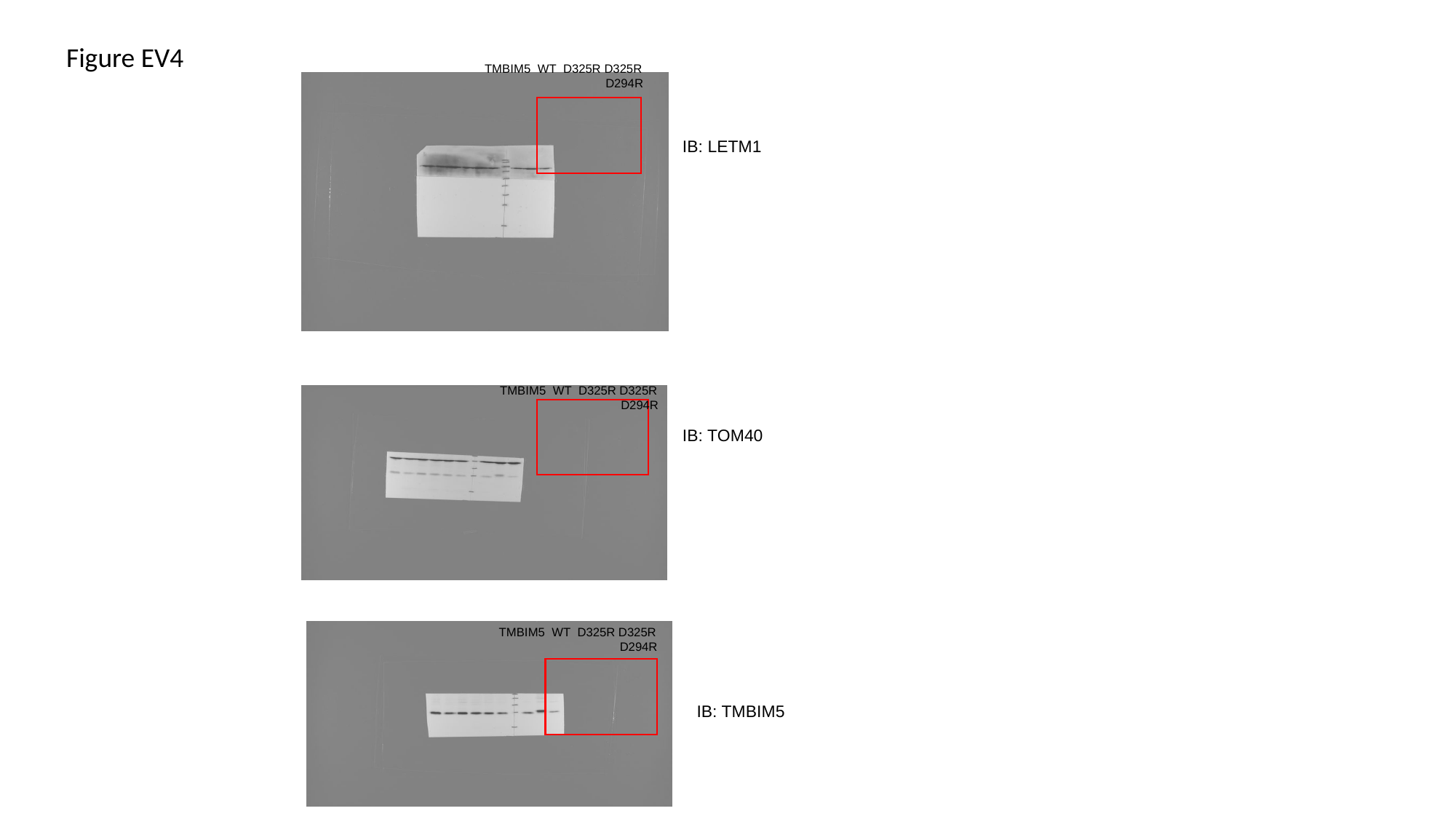

Figure EV4
TMBIM5 WT D325R D325R
 D294R
IB: LETM1
TMBIM5 WT D325R D325R
 D294R
IB: TOM40
TMBIM5 WT D325R D325R
 D294R
IB: TMBIM5

Supplement: Supplementary file 4 — Source Data for Expanded View and Appendix [file EMBR-23-e54978-s013.zip › Figure EV4 source data.pptx]

## Slide 1
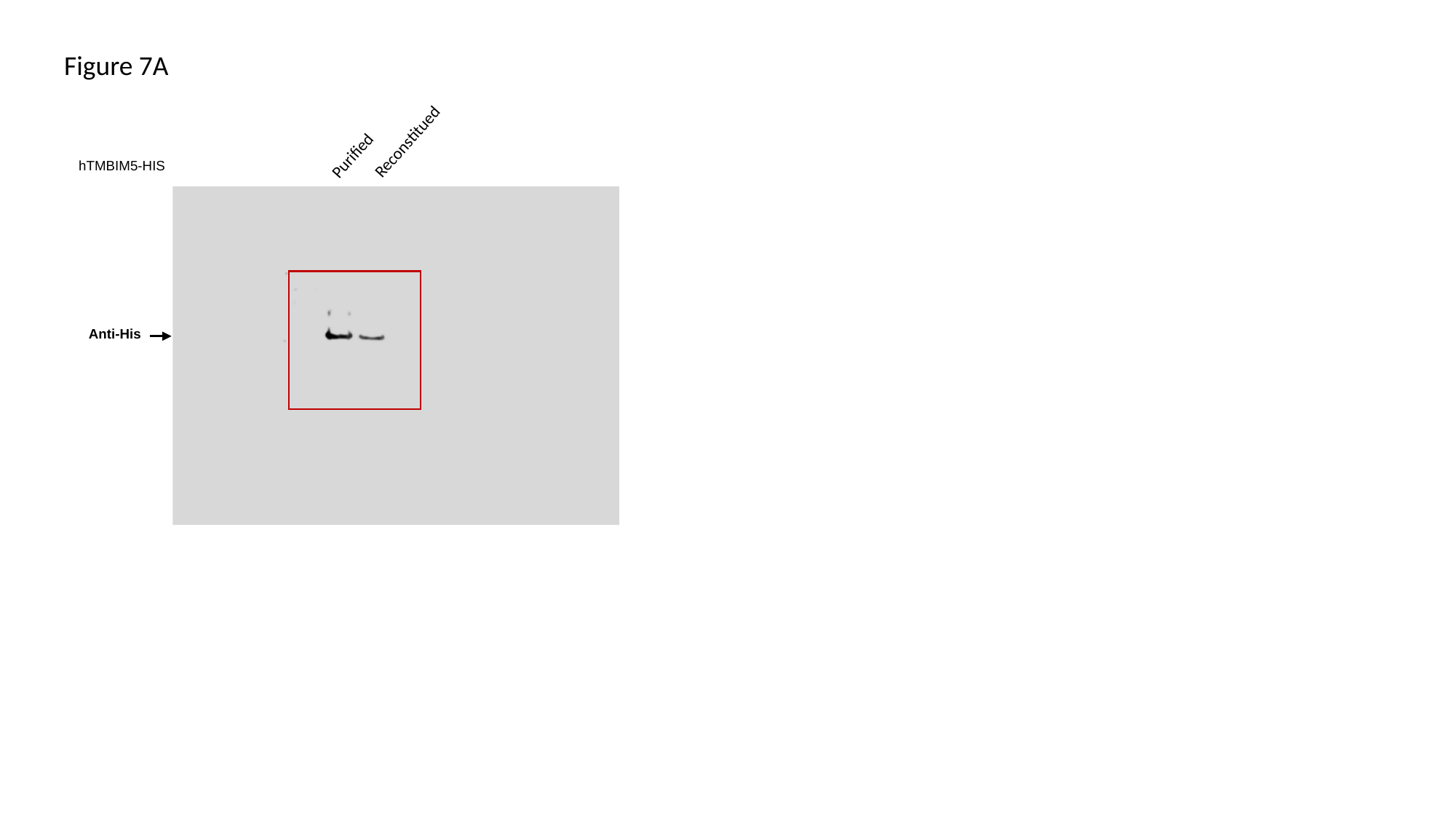

Figure 7A
Reconstitued
Purified
hTMBIM5-HIS
Anti-His

Supplement: Supplementary file 11 — Source Data for Figure 7 [file EMBR-23-e54978-s010.zip › Figure 7A_source data.pptx]
