# Supplementary material for: TMBIM5 is the Ca2+/H+ antiporter of mammalian mitochondria
Source: EMBO Rep. 2022 Nov 2;23(12):e54978. doi: 10.15252/embr.202254978 (PMC9724676; doi:10.15252/embr.202254978)
Supplement: Supplementary file 5 — Source Data for Figure 1 [file EMBR-23-e54978-s008.pptx]

## Slide 1
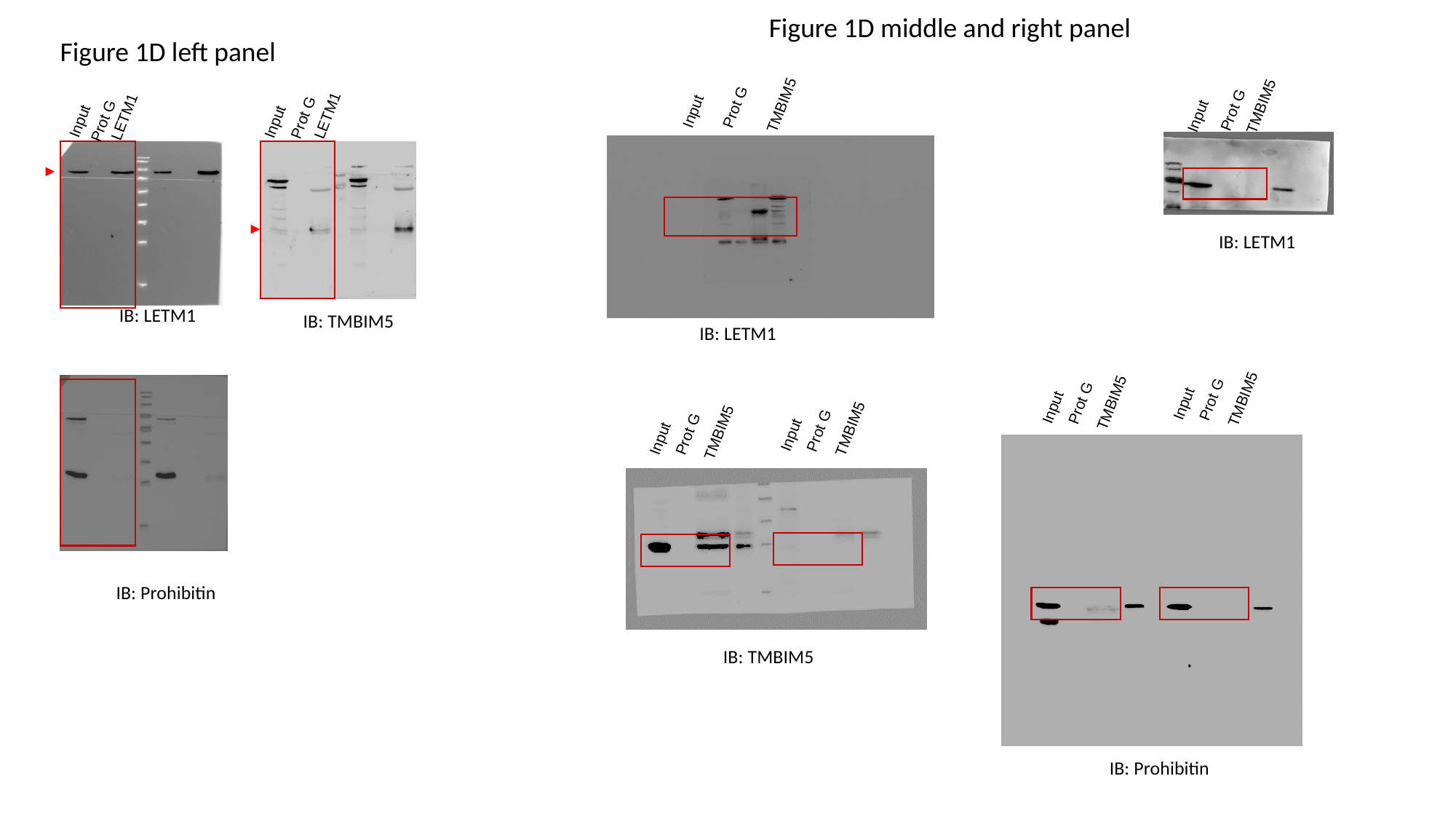

Figure 1D middle and right panel
Figure 1D left panel
TMBIM5
TMBIM5
Prot G
Prot G
Input
LETM1
LETM1
Input
Input
Prot G
Prot G
Input
IB: LETM1
IB: LETM1
IB: TMBIM5
IB: LETM1
TMBIM5
Prot G
TMBIM5
Prot G
Input
Input
TMBIM5
Prot G
TMBIM5
Prot G
Input
Input
IB: Prohibitin
IB: TMBIM5
IB: Prohibitin
