# Supplementary material for: TMBIM5 is the Ca2+/H+ antiporter of mammalian mitochondria
Source: EMBO Rep. 2022 Nov 2;23(12):e54978. doi: 10.15252/embr.202254978 (PMC9724676; doi:10.15252/embr.202254978)
Supplement: Supplementary file 6 — Source Data for Figure 2 [file EMBR-23-e54978-s003.zip › Figure 2A_source data.pptx]

## Slide 1
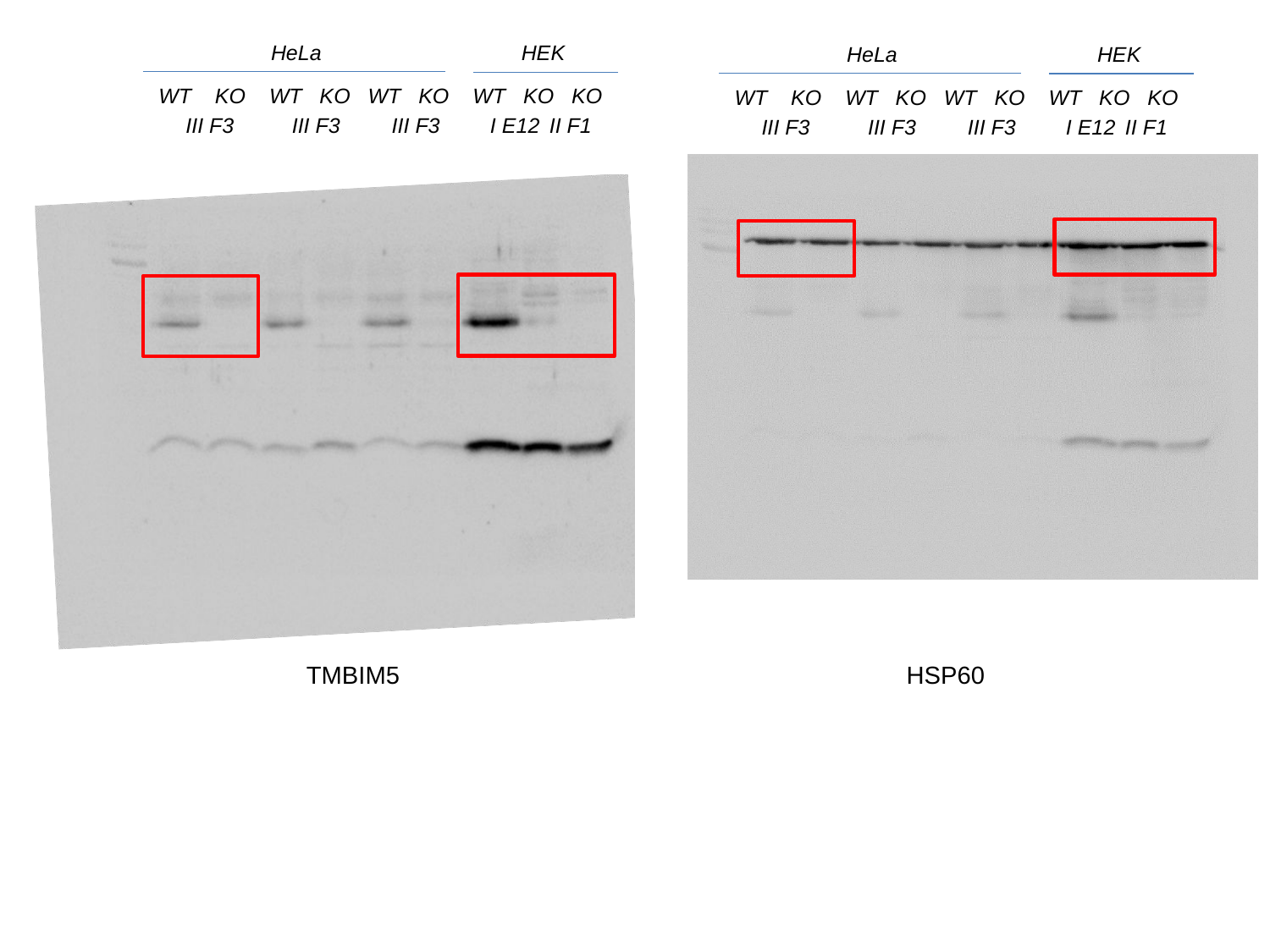

HeLa
HEK
HeLa
HEK
WT KO WT KO WT KO WT KO KO
WT KO WT KO WT KO WT KO KO
II F1
III F3
III F3
III F3
I E12
II F1
III F3
III F3
III F3
I E12
TMBIM5
HSP60
