# Supplementary material for: TMBIM5 is the Ca2+/H+ antiporter of mammalian mitochondria
Source: EMBO Rep. 2022 Nov 2;23(12):e54978. doi: 10.15252/embr.202254978 (PMC9724676; doi:10.15252/embr.202254978)
Supplement: Supplementary file 6 — Source Data for Figure 2 [file EMBR-23-e54978-s003.zip › Figure 2E_source data.pptx]

## Slide 1
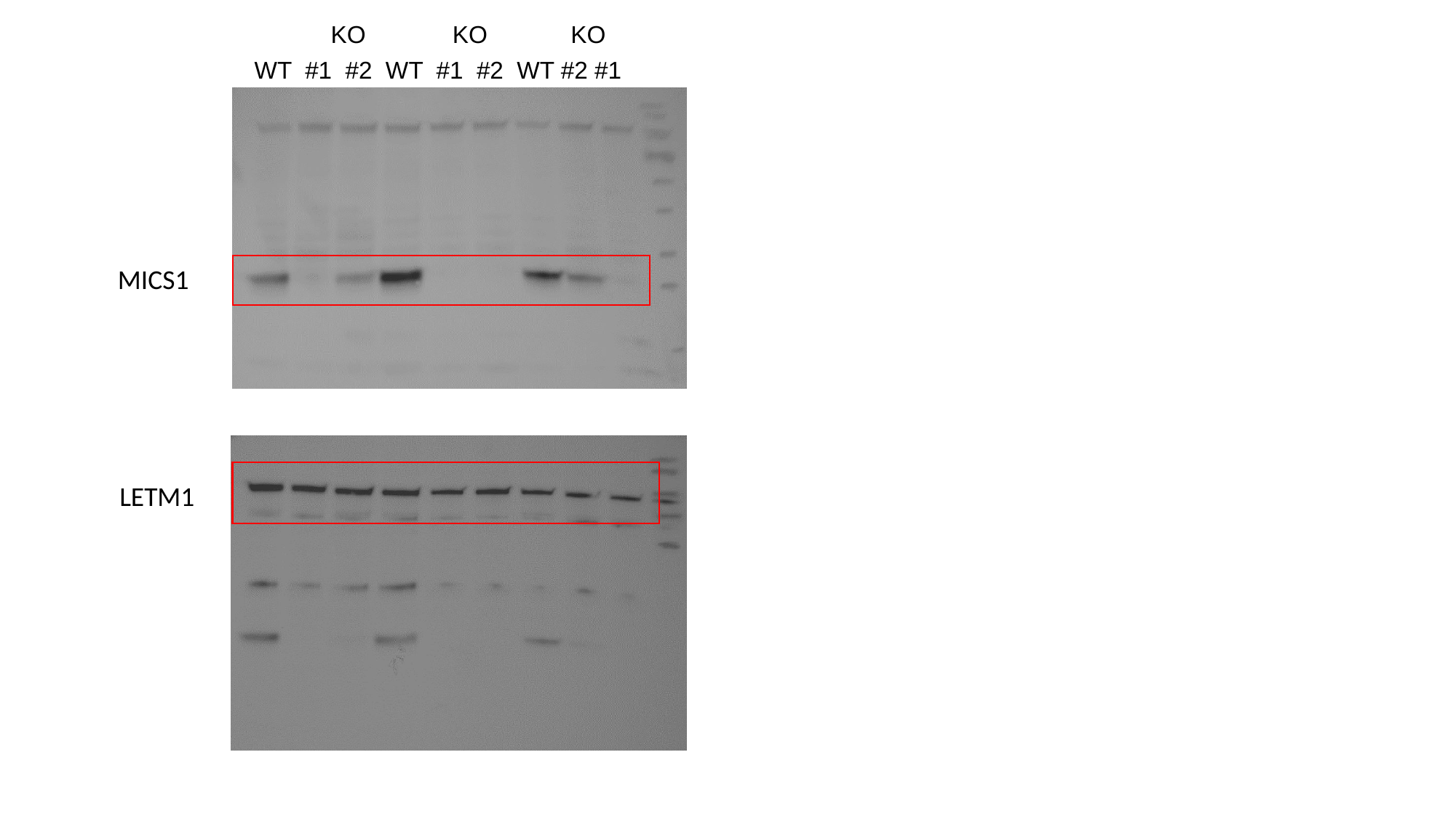

KO
KO
KO
WT #1 #2 WT #1 #2 WT #2 #1
MICS1
LETM1

## Slide 2
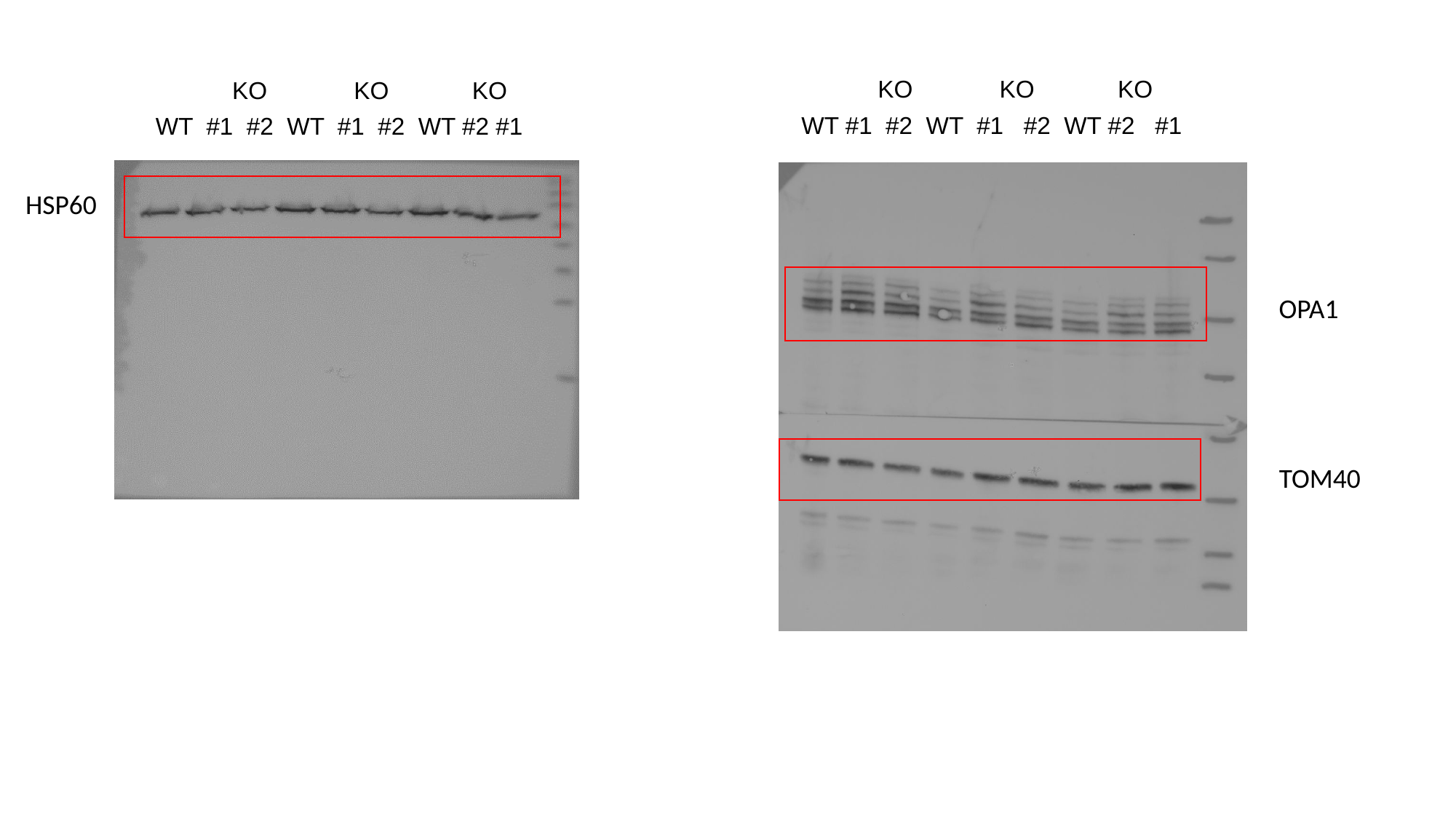

KO
KO
KO
KO
KO
KO
WT #1 #2 WT #1 #2 WT #2 #1
WT #1 #2 WT #1 #2 WT #2 #1
HSP60
OPA1
TOM40

## Slide 3
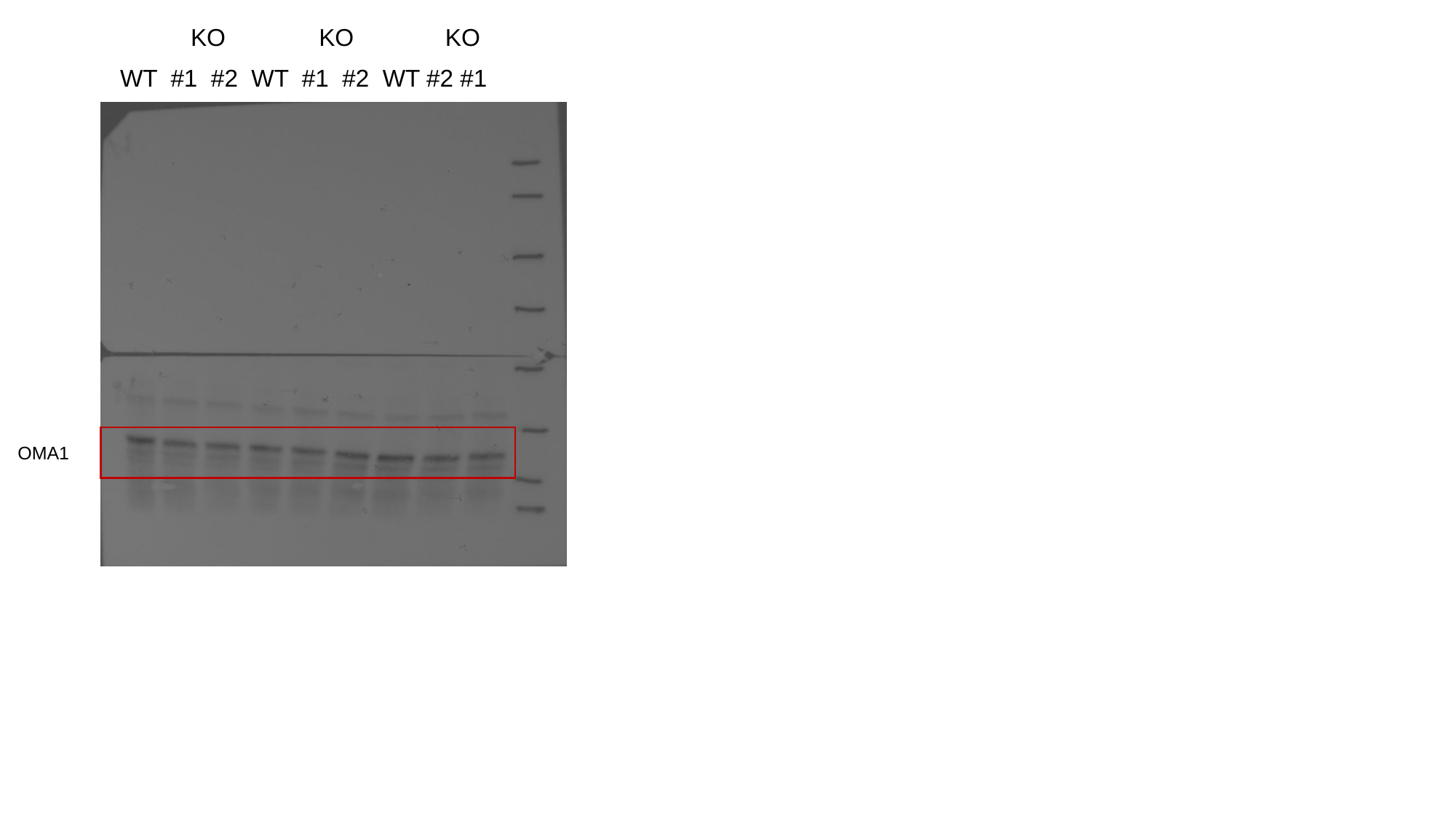

KO
KO
KO
WT #1 #2 WT #1 #2 WT #2 #1
OMA1

## Slide 4
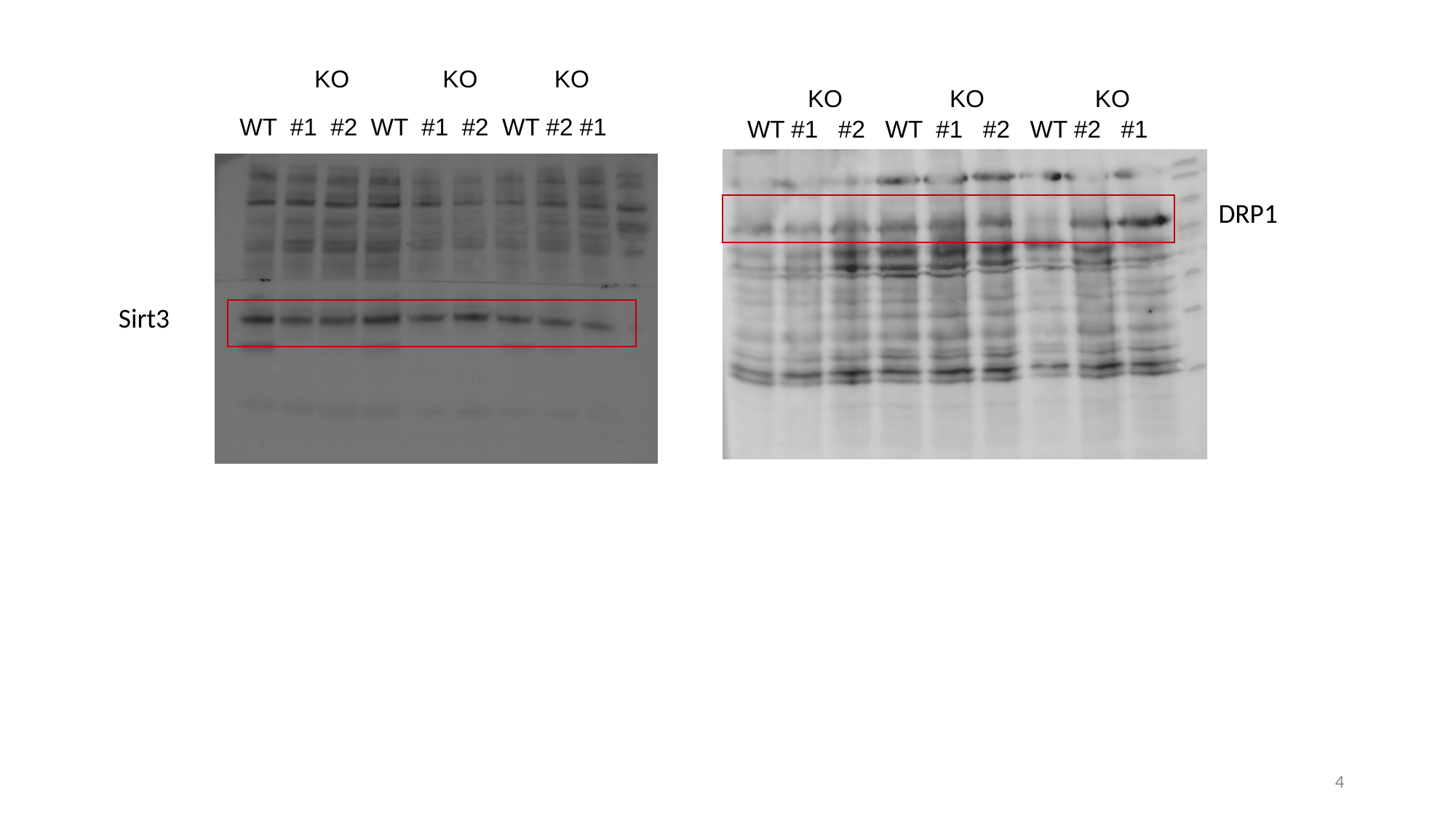

KO
KO
KO
KO
KO
KO
WT #1 #2 WT #1 #2 WT #2 #1
WT #1 #2 WT #1 #2 WT #2 #1
DRP1
Sirt3
4

## Slide 5
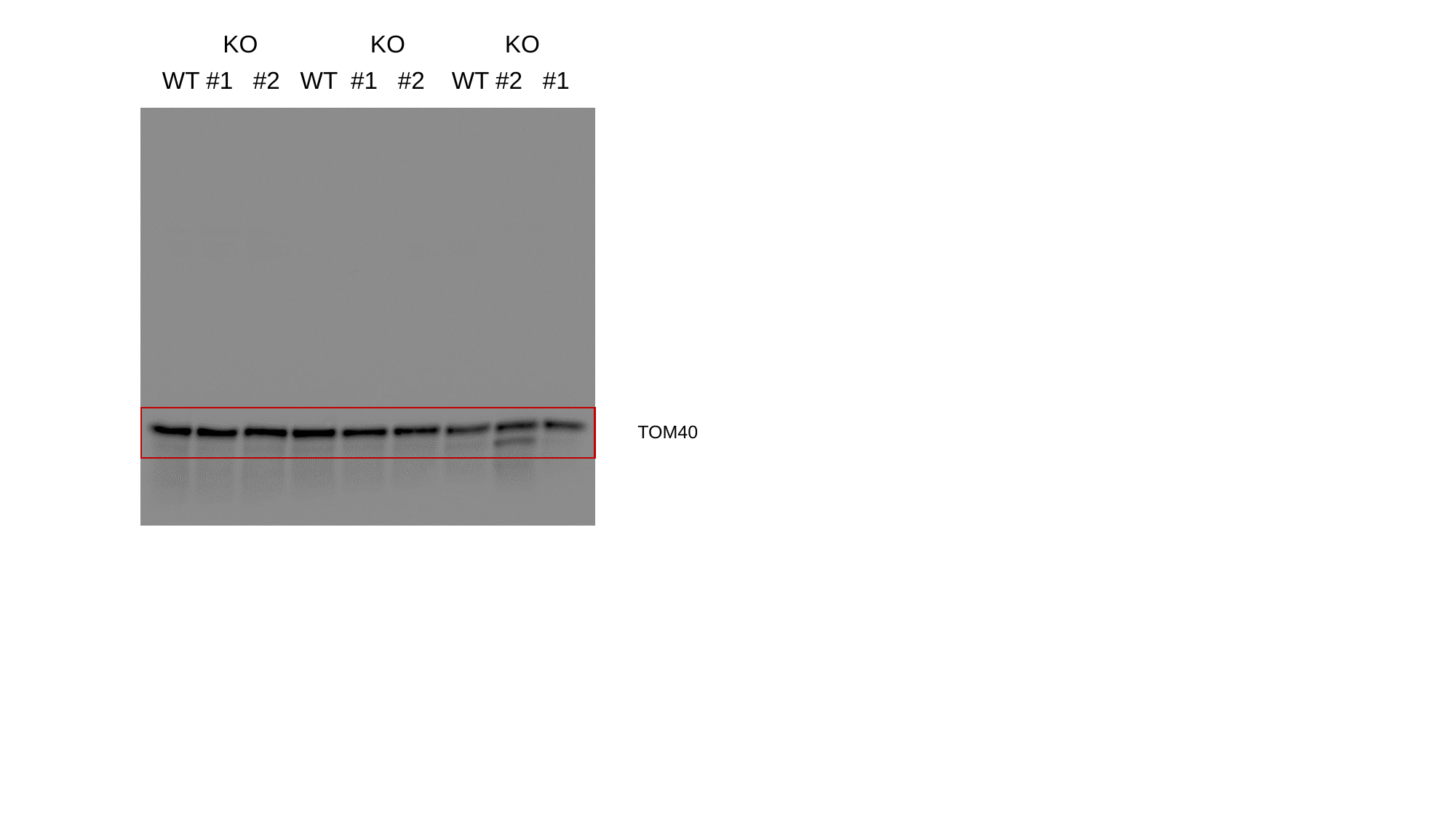

KO
KO
KO
WT #1 #2 WT #1 #2 WT #2 #1
TOM40
