# Supplementary material for: TMBIM5 is the Ca2+/H+ antiporter of mammalian mitochondria
Source: EMBO Rep. 2022 Nov 2;23(12):e54978. doi: 10.15252/embr.202254978 (PMC9724676; doi:10.15252/embr.202254978)
Supplement: Supplementary file 12 — PDF+ [file EMBR-23-e54978-s004.pdf]

Article

SOURCE DATA TRANSPARENT PROCESS OPEN ACCESS

EMBO reports

TMBIM5 is the Ca^{2+}/H^{+} antiporter of mammalian mitochondria

Shane Austin[1,†,‡], Ronald Mekis[1,2,†], Sami E M Mohammed[2,†], Mariafrancesca Scalise[3], Wen-An Wang[4], Michele Galluccio[3], Christina Pfeiffer[1], Tamara Borovec[1,2], Katja Parapatics[5], Dijana Vitko[5], Nora Dinhopl[6], Nicolas Demaurex[4], Keiryn L Bennett[5], Cesare Indiveri[3,7] & Karin Nowikovsky[1,2,*]

Abstract

Mitochondrial Ca^{2+} ions are crucial regulators of bioenergetics and cell death pathways. Mitochondrial Ca^{2+} content and cytosolic Ca^{2+} homeostasis strictly depend on Ca^{2+} transporters. In recent decades, the major players responsible for mitochondrial Ca^{2+} uptake and release have been identified, except the mitochondrial Ca^{2+}/H^{+} exchanger (CHE). Originally identified as the mitochondrial K^{+}/H^{+} exchanger, LETM1 was also considered as a candidate for the mitochondrial CHE. Defining the mitochondrial interactome of LETM1, we identify TMBIM5/MICS1, the only mitochondrial member of the TMBIM family, and validate the physical interaction of TMBIM5 and LETM1. Cell-based and cell-free biochemical assays demonstrate the absence or greatly reduced Na^{+}-independent mitochondrial Ca^{2+} release in TMBIM5 knockout or pH-sensing site mutants, respectively, and pH-dependent Ca^{2+} transport by recombinant TMBIM5. Taken together, we demonstrate that TMBIM5, but not LETM1, is the long-sought mitochondrial CHE, involved in setting and regulating the mitochondrial proton gradient. This finding provides the final piece of the puzzle of mitochondrial Ca^{2+} transporters and opens the door to exploring its importance in health and disease, and to developing drugs modulating Ca^{2+} exchange.

Keywords LETM1; mitochondrial Ca^{2+}-H^{+} exchanger; mitochondrial metabolism; permeability transition pore; TMBIM5 (MICS1)
Subject Categories Membrane & Trafficking; Organelles
DOI 10.15252/embr.202254978 | Received 4 March 2022 | Revised 7 September 2022 | Accepted 7 October 2022 | Published online 2 November 2022
EMBO Reports (2022) 23: e54978

Introduction

Ion homeostasis is critical for mitochondrial function. The dynamic balance of cations is achieved by a set of integrated transport systems for K^{+}, Na^{+}, and Ca^{2+}. Loss of this balance between cation uptake and release has consequences for the organelle and ultimately the cell, and includes mitochondrial swelling, disrupted cristae structure, deregulated bioenergetics, and may result in cell death. Intracellularly, mitochondria are major sinks of Ca^{2+}, an ion of comparatively low concentration to K^{+} and Na^{+}. The role of mitochondrial Ca^{2+} buffering has been extensively studied (Giorgi *et al*, 2018; Pallafacchina *et al*, 2018), yet some of the players in maintaining Ca^{2+} balance have not been identified (De Stefani *et al*, 2016; Urbani *et al*, 2020). One of the missing pieces in this molecular puzzle is the Na^{+}-independent Ca^{2+} efflux pathway, a putative Ca^{2+}/H^{+} exchanger (CHE). This exchanger, whose existence has been postulated since the 1970s (Carafoli *et al*, 1974) is critical for maintaining mitochondrial Ca^{2+} levels and pH homeostasis.

One of the CHE candidate proteins is LETM1. LETM1, a single transmembrane domain-containing protein, has initially been characterized as the mitochondrial K^{+}/H^{+} exchanger (KHE) (Nowikovsky *et al*, 2004, 2007; Hasegawa & van der Bliek, 2007; McQuibban *et al*, 2010; Hashimi *et al*, 2013). The proposal that LETM1 could also be a CHE was based on a *Drosophila* S2 genome-wide RNAi screen of modulators of mitochondrial Ca^{2+} transport (Jiang *et al*, 2009). Subsequent studies have confirmed an involvement of LETM1 in Ca^{2+} and K^{+} transport, but key questions remained, perhaps the most important being how a single transmembrane protein can mediate a process of ion exchange (Nowikovsky & Bernardi, 2014; Austin & Nowikovsky, 2019, 2021). It appeared possible that LETM1 acts as a multimer, or as part of a protein complex. The first possibility is supported by cryo-EM structures of LETM1 oligomers, which facilitated pH-dependent Ca^{2+}-movement in a

1 Department of Internal Medicine I and Comprehensive Cancer Center, Medical University of Vienna, Vienna, Austria
2 Department of Biomedical Sciences, Institute of Physiology, Pathophysiology and Biophysics, University of Veterinary Medicine Vienna, Vienna, Austria
3 Department DiBEST (Biologia, Ecologia, Scienze della Terra) Unit of Biochemistry and Molecular Biotechnology, University of Calabria, Arcavacata di Rende, Italy
4 Department of Cell Physiology & Metabolism, University of Geneva, Geneva, Switzerland
5 CeMM Research Center for Molecular Medicine of the Austrian Academy of Sciences, Vienna, Austria
6 Department of Pathobiology, Institute of Pathology, University of Veterinary Medicine, Vienna, Austria
7 CNR Institute of Biomembranes, Bioenergetics and Molecular Biotechnologies (IBIOM), Bari, Italy
*Corresponding author. Tel: +43 125077/4573; E-mail: karin.nowikovsky@vetmeduni.ac.at
†These authors contributed equally to this work
‡Present address: Department of Biological & Chemical Sciences, The University of the West Indies, Cave Hill Campus, Cave Hill, Barbados

© 2022 The Authors. Published under the terms of the CC BY 4.0 license.

cell-free system (Shao *et al*, 2016). Whether LETM1 is part of a protein complex remains unaddressed.

Here, we searched for partners of LETM1 and found the interactor Transmembrane BAX Inhibitor Motif containing protein 5 (TMBIM5), also called Mitochondrial Morphology and Cristae Structure 1 (MICS1), a member of the TMBIM family, which has been implicated in the regulation of intracellular Ca^{2+} by a number of studies (Hung *et al*, 2011; Carrara *et al*, 2012; Lisak *et al*, 2015; Rojas-Rivera & Hetz, 2015; Liu, 2017; Kim *et al*, 2021). TMBIM5/MICS1 is the only species with a mitochondrial localization (Oka *et al*, 2008) while other TMBIM family members are localized to the ER, Golgi, and plasma membrane (Rojas-Rivera & Hetz, 2015). Importantly, TMBIM5 was reported as a regulator of Ca^{2+} and apoptosis (Oka *et al*, 2008; Lisak *et al*, 2015). Here, we demonstrate that TMBIM5 is the long-sought mitochondrial CHE, a crucial component of mitochondrial Ca^{2+} homeostasis.

Results

TMBIM5/MICS1 interacts with LETM1

To determine the interactome of LETM1, we generated HEK293 cell lines with inducible expression of LETM1 fused to hemagglutinin and streptavidin (SH) as originally described in Glatter *et al* (2009) and Rudashevskaya *et al* (2013). We applied a powerful two-step tandem affinity purification (TAP) approach to identify high-confidence interaction partners of LETM1-SH from whole cell lysates and one-step affinity purification (AP) coupled with mass spectrometry (MS) from isolated mitochondria (Fig 1A). In the latter approach, only the streptavidin component of the tag was utilized and the protein complex eluted with biotin. As few studies have used the limited amounts of material from isolated organelles for AP-MS, we first assessed the reliability of our one-step method to investigate organellar interactomes. As a benchmark, the inner mitochondrial membrane protein mitochondrial Ca^{2+} uniporter (MCU) was fused to SH. The members of the published core interactome were identified except for the tertiary interactor MICU2 (Sancak *et al*, 2013; Fig 1B and Appendix Fig S1A). Thus, the method was sufficiently robust to cover approximately 75% of the MCU mitochondrial core interactome and therefore likely to detect other mitochondrial interactomes with similar accuracy. Next, we determined the LETM1 interactome using TAP- and AP-MS from both whole cells and isolated mitochondria (Dataset EV1), obtaining 31 overlapping proteins (Appendix Fig S1B), including TBK1, a protein previously observed to interact with LETM1 in similar AP-MS studies (Li *et al*, 2011). We compared these 31 proteins to nonspecific interactors from similar AP-MS experiments using the data from the CRAPome (Mellacheruvu *et al*, 2013b) and were able to identify 12 high-confidence interactors with scores greater than 0.95 (Fig 1C and Appendix Fig S1C).

Of immediate interest was TMBIM5, an inner mitochondrial membrane protein with 8 predicted transmembrane helices (https://alphafold.ebi.ac.uk/entry/Q9H3K2). Similar to LETM1, TMBIM5 is involved in the regulation of mitochondrial structure (Oka *et al*, 2008; Seitaj *et al*, 2020). The interaction of TMBIM5 and LETM1 was confirmed with co-IP and reverse co-IP experiments with TMBIM5 and LETM1 antibodies (Fig 1D left panels). Probing for mitochondrial Prohibitin demonstrated this interaction was not an unspecific enrichment of membrane-associated proteins. Furthermore, immunoblots of blue native gel electrophoresis indicated that LETM1 and TMBIM5 both migrated equally at the estimated mass of ~400 and ~700 kDa (Fig 1D right and Appendix Fig S1D). In the absence of TMBIM5, the signals for both TMBIM5 and LETM1 at ~700 kDa markedly decreased and those at ~400 kDa became weaker for TMBIM5 but not for LETM1. These data suggest that the proteins oligomerize in protein complexes of ~400 and 700 kDa and that LETM1 requires TMBIM5 to oligomerize in the latter. TMBIM5-containing complexes in HeLa mitochondria were comparable, and their levels markedly increased when LETM1 was knocked-down, suggesting that TMBIM5 may compensate for the decrease in LETM1, without changing the mass of the complex (Fig EV1).

TMBIM5 depletion impairs mitochondrial bioenergetics and morphology

We generated TMBIM5 stable knockdown (KD) cells by short hairpins targeting various exons. TMBIM5KD cells had up to

Figure 1. LETM1 and TMBIM5 interact.

A Scheme illustrating workflow for miniaturized AP-MS experiments, left to right: whole cells or isolated mitochondria were lysed or solubilized, respectively. The cell/mitochondrial lysates were used for affinity purification (AP) using the STREP tag and tandem affinity purification (TAP) using STREP and HA tag found on the bait protein. Eluates of the AP and control experiments were reduced, alkylated, and digested by trypsin. Peptides are purified on a C18 stage tip and then run on an LTQ Orbitrap Velos. Protein identifications were made by internal tools using MASCOT and Phenyx and the removal of nonspecific interactors done using the CRAPome. Created with Biorender.com.

B MCU was selected as a model protein, the functional complex consists of the five proteins above (MCU, MCUb, MICU1, MICU2, EMRE). Note that an additional tissue-specific tertiary interaction partner (MICU3) is only expressed at very low levels in HEK293 cells (Diego De Stefani, personal communication). Illustration adapted from Sancak *et al* (2013).

C All high-confidence interaction partners of LETM1 are shown as nodes. Node color indicates SAINT score, a probability-based measure of interaction confidence. See also Appendix Fig S1A–C.

D Co-immunoprecipitation of TMBIM5 and LETM1 protein in tandem in the left 3 panels. The input represents the mitochondrial crudely isolated from HEK293 cells and was used as input for the co-IP, LETM1 was immunoprecipitated (left panel, IP: LETM1) using a LETM1 monoclonal antibody and Protein G magnetic beads (ProtG). ProtG beads alone were used as a negative control for binding, immunoprecipitates were immunoblotted (IB) for the indicated proteins to demonstrate interaction. 10% of the input was used for immunoblotting. Prohibitin (PHB) was used as a control to illustrate no nonspecific binding of inner mitochondrial membrane protein complexes. The middle and right panel of the co-IPs illustrates the converse experiment, in the middle in TMBIM5WT and right TMBIMKO, using TMBIM5 as bait (right panel, IP: TMBIM5). The last two right panels show blots from BN–PAGE conducted in TMBIM5WT and KO.

Source data are available online for this figure.

 © 2022 The Authors

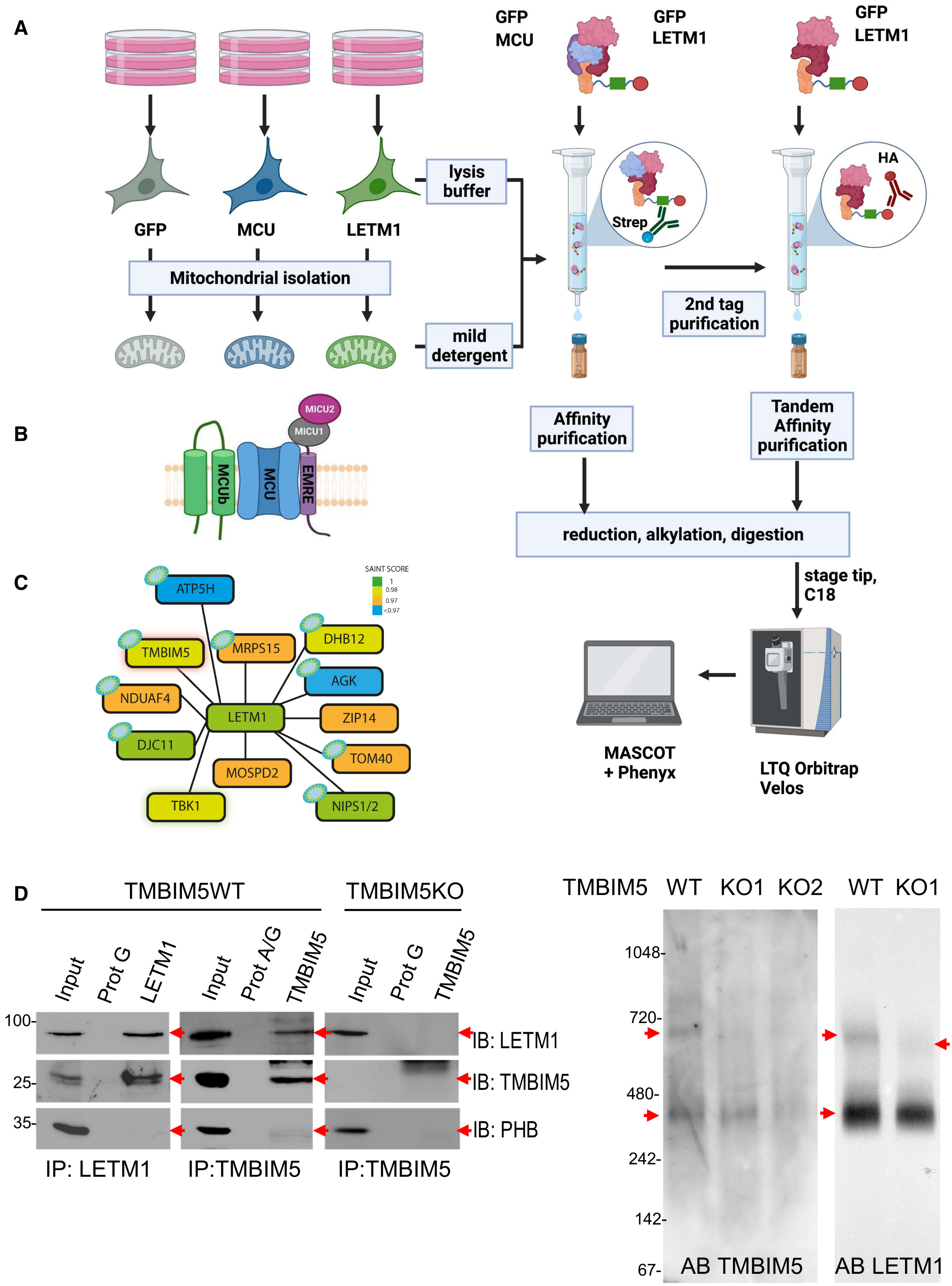

A
GFP
MCU
LETM1
lysis buffer
Mitochondrial isolation
mild detergent
GFP MCU
GFP LETM1
Strep
HA
2nd tag purification
Affinity purification
Tandem Affinity purification
reduction, alkylation, digestion
stage tip, C18
MASCOT + Phenyx
LTQ Orbitrap Velos
B
MICU2
MICU1
MCUb
MCU
EMRE
C
SAINT SCORE
1
0.98
0.97
<0.97
ATP5H
TMBIM5
MRPS15
DHB12
AGK
NDUAF4
LETM1
ZIP14
DJC11
TOM40
MOSPD2
TBK1
NIPS1/2
D
TMBIM5WT
TMBIM5KO
Input
Prot G
LETM1
Input
Prot A/G
TMBIM5
Input
Prot G
TMBIM5
100-
25-
35-
IB: LETM1
IB: TMBIM5
IB: PHB
IP: LETM1
IP:TMBIM5
IP:TMBIM5
TMBIM5 WT KO1 KO2
WT KO1
1048-
720-
480-
242-
142-
67-
AB TMBIM5
AB LETM1

Figure 1.

80% reduced TMBIM5 levels compared with scrambled controls with matching decrease in LETM1 (Fig EV2A). The proliferation rate of TMBIM5KD cells in a glucose-containing medium was reduced marginally and only the final time point being significantly affected (Fig EV2B). TMBIM5KD respiratory parameters were not significantly affected (Fig EV2C and D), in contrast to severely compromised galactose-dependent respiration (Fig EV2E and F), indicating that TMBIM5 impacts mitochondrial function.

To address the specific function of TMBIM5 in mitochondrial morphology and cation homeostasis, we generated TMBIM5 knockout (KO) HEK293 and HeLa cells by CRISPR/Cas9 genome editing. At the gene expression level, we obtained HEK293 and HeLa knockout KO individual clones with entirely abrogated transcript levels of TMBIM5. At the protein level, the total knockout was confirmed in HeLa cells clone IIIF3 (HeLa TMBIM5KO) and in HEK293 cells clone IIF1 (HEK293 TMBIM5KO1). In several other clones, translation was not entirely abolished, like in HEK293 clone IE12 (HEK293 TMBIM5KO2; Fig 2A and E). These clones were used in parallel when indicated to exclude off-target effects or check for gene dose effects. Cell growth was somewhat slowed under TMBIM5 depletion especially in TMBIM5KO2 (Fig 2B). As previously shown for HeLa and HAP cells (Oka *et al*, 2008; Seitaj *et al*, 2020), compared with wild-type (WT) cells HEK293 TMBIM5KO1 and TMBIM5KO2 displayed fragmented and less elongated mitochondria, respectively (Fig 2C). Electron micrographs showed TMBIM5KO mitochondria with swollen sections and altered cristae structures, cristae being also affected in the incomplete TMBIM5KO2 (Fig 2D, arrows). LETM1 levels were somewhat reduced in TMBIM5KO (Fig 2E and F). Since TMBIM5 is involved in cristae structures (Oka *et al*, 2008), and OPA1 controls cristae volume and junction organization, critical for mitochondrial cytochrome c retention (Olichon *et al*, 2003; Del Dotto *et al*, 2017), we investigated whether cristae structure and OPA1-cleavage pattern were coupled. OPA1 subunits c and e, both cleavage products of OMA1, appeared to increase in HEK293 TMBIM5KO compared with controls (Fig 2E and F). Consistent with the autocatalytic degradation of activated OMA1, TMBIM5KO had significantly reduced levels of OMA1 (Fig 2E and F). Furthermore, DRP1 was upregulated (Fig 2E and F), matching the shift toward mitochondrial fission and stress-sensitive activation of OMA1 and OMA1-dependent OPA1 cleavage. Thus, TMBIM5KO affected the dynamics of OPA1-dependent cristae structures (Fig 2E and F).

Mitochondrial KHE requires LETM1 and TMBIM5

The interaction of TMBIM5 with the mitochondrial KHE component LETM1 (Nowikovsky *et al*, 2012), raised the question of whether TMBIM5 contributes to KHE activity. Light scattering methods have been classically used to monitor the swelling of mitochondria (Mitchell, 1966; Bernardi, 1999). Previous studies have established that potassium acetate- (KOAc-) induced passive swelling is impaired in LETM1KD mitochondria. HeLa and HEK293 TMBIM5KO mitochondria showed a reduced initial optical density compared with TMBIM5WT, suggesting enlarged mitochondria. TMBIM5KO mitochondria also swell significantly less than TMBIM5WT in KOAc media (Fig 3A–D) as also seen for LETM1KD mitochondria (Fig 3E and F) and Austin *et al* (2017). Re-expression of TMBIM5 in HEK293 TMBIM5KO cells restored the swelling amplitude to WT levels (Fig 3A and B). Thus, TMBIM5KO led to swollen mitochondria and lower KHE activity, perhaps by reducing LETM1 levels and/or function, or by disrupting the osmotic balance through the overload of another ion.

TMBIM5 mediates mitochondrial Na^+-independent Ca^{2+} efflux

The TMBIM protein family controls intracellular Ca^{2+}, and a pH-sensitive Ca^{2+} channel function has been proposed for the bacterial TMBIM-homolog BsYetJ (Guo *et al*, 2019b). We asked whether TMBIM5 controls mitochondrial Ca^{2+} homeostasis through Ca^{2+}/H^+ exchange. To this end, we performed mitochondrial Ca^{2+} uptake and release assays in digitonin-permeabilized HEK293 cells pulsed with external Ca^{2+}. TMBIM5WT or TMBIM5KO mitochondria were able to release Ca^{2+} after inhibition of Ca^{2+} uptake with the selective MCU inhibitor ruthenium red (RR). To exclude Na^+-dependent Ca^{2+} fluxes, we next used the NCLX inhibitor CGP37157 or NCLXKD (Appendix Fig S2A–D). TMBIM5WT and TMBIM5KO mitochondria exhibited similar rates of energy-dependent Ca^{2+} uptake (Fig 4 and Appendix Fig S3A, B, and D–G). Remarkably, TMBIM5KO mitochondria displayed dramatically decreased RR-induced mitochondrial Ca^{2+} release, which was proportional to the depletion of TMBIM5 (Fig 4A–D red and orange traces). TMBIM5WT and TMBIM5KO mitochondria readily released Ca^{2+} upon the addition of alamethicin or FCCP, suggesting that they had comparable levels of matrix Ca^{2+}. Re-expression of TMBIM5 in HEK293 TMBIM5KO cells was able to restore Ca^{2+} efflux (Fig 4A and B). TMBIM5WT HeLa mitochondria (Fig EV3) released matrix Ca^{2+} comparably to

Figure 2. TMBIM5KO causes mitochondrial matrix swelling and cristae disorganization.

A Western blot analysis of TMBIM5 in control and targeted HeLa and HEK293 clones.

B Proliferation assay of HEK293 cells in the function of TMBIM5. Graph shows the mean ± SD of three individual counts, One-way ANOVA with the Dunnett's multiple comparisons test performed against TMBIM5WT $^*P = 0.0155$.

C Live imaging of HEK293 TMBIM5WT and KO cells stained with MitoTracker Green FM. Scale bars: 10 μm.

D Alteration of the mitochondrial ultrastructure shown by transmission electron microscopy, red arrow pointing to the dilated matrix. Wider mitochondria in the middle and right panel compared with controls, a middle panel showing the strongest phenotype of matrix width and cristae forms. Scale bars: 1 μm.

E Isolated mitochondria from three independent replicates of HEK293 TMBIM5WT, and TMBIM5KO1 and KO2 were analyzed by immunoblotting using the indicated antibodies, HSP60 and TOM40 served as mitochondrial loading controls.

F Densitometric analysis of the bands in (E) normalized to loading control, bar graph of three individual experiments (biological replicates), mean ± SD, one-way ANOVA with the Bonferroni's multiple comparisons test performed against TMBIM5WT $^*P < 0.05$, $^{**}P < 0.008$, two-way ANOVA with the Bonferroni's multiple comparisons test performed for the OPA1 statistics against TMBIM5WT, $^{***}P = 0.0009$, $^{****}P < 0.0001$.

Source data are available online for this figure.

 © 2022 The Authors

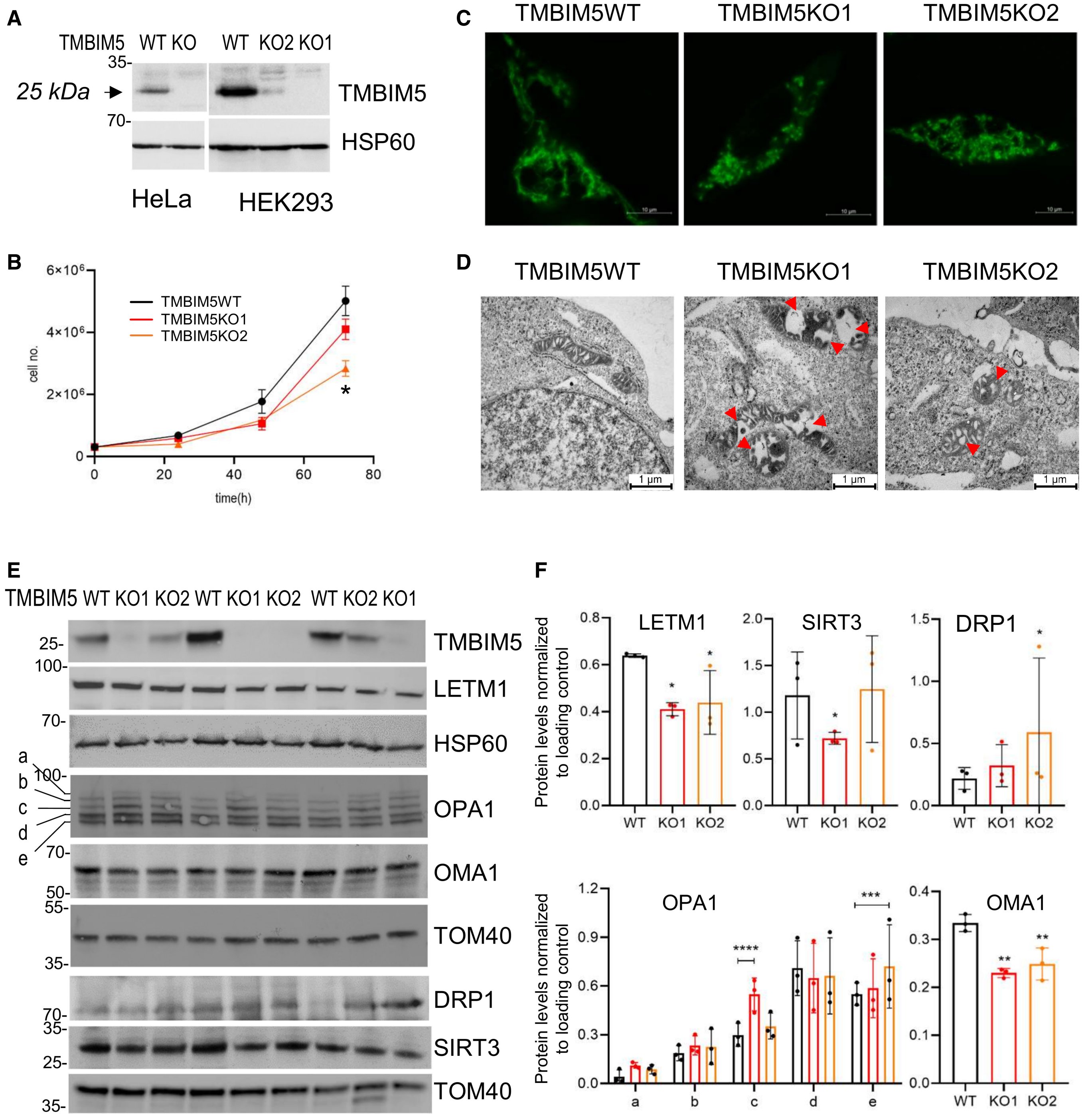

Figure 2.

HEK293, especially when applying a higher dose of Ca^{2+} pulse, possibly due to more prominent Ca^{2+} buffering whereas TMBIM5KO HeLa mitochondria remained refractory to Na^{+}-independent Ca^{2+} release (Fig EV3A–D). Re-expression of TMBIM5 restored Ca^{2+} efflux (Fig EV3C and D), confirming the CHE function of TMBIM5 in another mammalian cell type.

To assess whether the permeability transition pore (PTP) contributes to the recorded Ca^{2+} fluxes in absence of NCLX, we repeated Ca^{2+} uptake/efflux assays in presence of cyclosporin A (CsA), the PTP desensitizer (Basso *et al*, 2008). TMBIM5WT displayed comparable Ca^{2+} efflux as in the absence of CsA, indicating that Na^{+}-independent Ca^{2+} release was also independent of PTP flickering or opening (Fig 4E and F).

The TMBIM protein family shares a conserved aspartyl dyad that affects Ca^{2+} binding and ensures the pH sensitivity of Ca^{2+} transport (Guo *et al*, 2019b). Mutation of the predicted pH-sensitive site D325

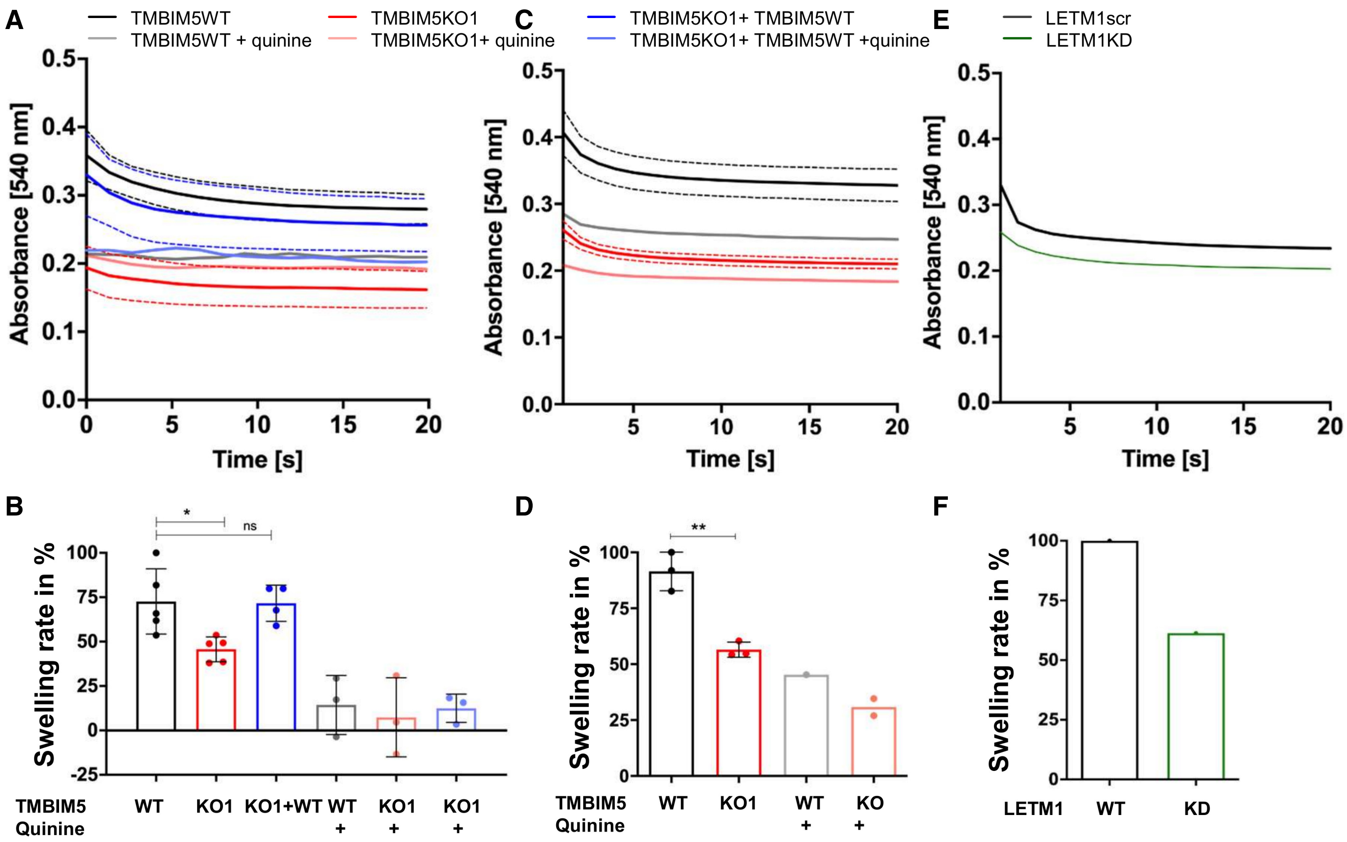

Figure 3. TMBIM5 and LETM1 are involved in mitochondrial KHE activity.

A–F KOAc-induced swelling was measured in mitochondria from HEK293 TMBIM5WT, TMBIM5KO and TMBIM5KO cells stably re-expressing TMBIM5WT (A, B), HeLa TMBIM5WT and TMBIM5KO (C, D) and HeLa LETM1 scramble (scr) and LETM1KD (E, F) cells. TMBIM5WT: black traces/bars, TMBIM5KO: red traces/bars, TMBIM5KO + TMBIM5WT: blue traces/bars, LETM1scr: black trace/bar, LETM1KD: green trace/bar. (B–E) Quantification of swelling amplitudes, data shown in (B, D) are the mean ± SD from three to five independent experiments (biological replicates). (B) HEK293 TMBIM5WT (100 ± 19.71) and HEK293 TMBIM5KO (48.08 ± 11.906). TMBIM5KO + TMBIM5WT (90.55 ± 12.93). (D) HeLa TMBIM5WT (100 ± 9.47) and HeLa TMBIM5KO (63.48 ± 8.60). Lower basal optical density indicates swollen matrix prior KOAc addition; Inhibition of KHE with quinine (WT gray bar, 18.14 ± 21.02; KO1 pink bar, 9.33 ± 28.17; TMBIM5KO + TMBIM5WT, light blue 15.79 ± 10.04. Statistical analysis: One-Way ANOVA with Bonferroni correction (*$P < 0.05$, **$P < 0.01$) against HEK293 TMBIM5WT and an unpaired student's *t*-test (**$P < 0.01$) against HeLaTMBIM5.

Source data are available online for this figure.

did not lower TMBIM5 protein levels (Fig EV4) but significantly decreased Ca^{2+} release from TMBIM5KO mitochondria stably expressing TMBIM5^{D325R} (Fig 4G and H). This result confirmed that D325 is required to allow Ca^{2+} release by TMBIM5. By contrast, TMBIM5^{D325R} did not decrease the amplitude of KOAc-dependent swelling (Appendix Fig S4) suggesting that protein interaction rather than changes in mitochondrial Ca^{2+} or pH affect LETM1 activity.

We next examined the CHE function of TMBIM5 in intact HEK293 cells. Since NCLX is responsible for most of the mitochondrial efflux, we compared TMBIM5WT and TMBIM5KO in an NCLXKD background to unmask TMBIM5-dependent Ca^{2+} fluxes. The mitochondrial Ca^{2+} elevations evoked by the Ca^{2+}-mobilizing agonists ATP and carbachol were increased and prolonged in TMBIM5KO cells. By contrast, the presence of TMBIM5 could compensate for the loss of NCLX since mitochondrial Ca^{2+} levels returned to basal levels in ~4 min (Fig 5A and B). The fourfold reduction in the mitochondrial efflux rates in TMBIM5KO confirmed that NCLX-independent mitochondrial Ca^{2+} release mediated by TMBIM5 also occurs in intact cells.

Altogether, the data confirmed that mitochondria can extrude matrix Ca^{2+} through an NCLX-independent pathway, which is widely assumed to be a CHE, and that TMBIM5 mediates CHE activity.

We next examined Ca^{2+} fluxes in HEK293 LETM1KD. The presence or absence of LETM1 (Fig 4I and J, and Appendix Fig S5A and B) did not alter Ca^{2+} uptake (Appendix Fig S3C) nor the Na^+-independent Ca^{2+} fluxes (Fig 4G and H). Since depletion of TMBIM5 or LETM1 reduces KHE activity, we asked whether increasing KHE activity would restore Ca^{2+} release in TMBIM5KO mitochondria. Therefore, we repeated the previous experiment in the presence of nigericin, a highly selective ionophore catalyzing KHE. Nigericin did not restore Ca^{2+} efflux (Fig 4K and L), suggesting that Na^+-independent Ca^{2+} efflux requires TMBIM5 but not LETM1 or LETM1-mediated KHE activity.

 © 2022 The Authors

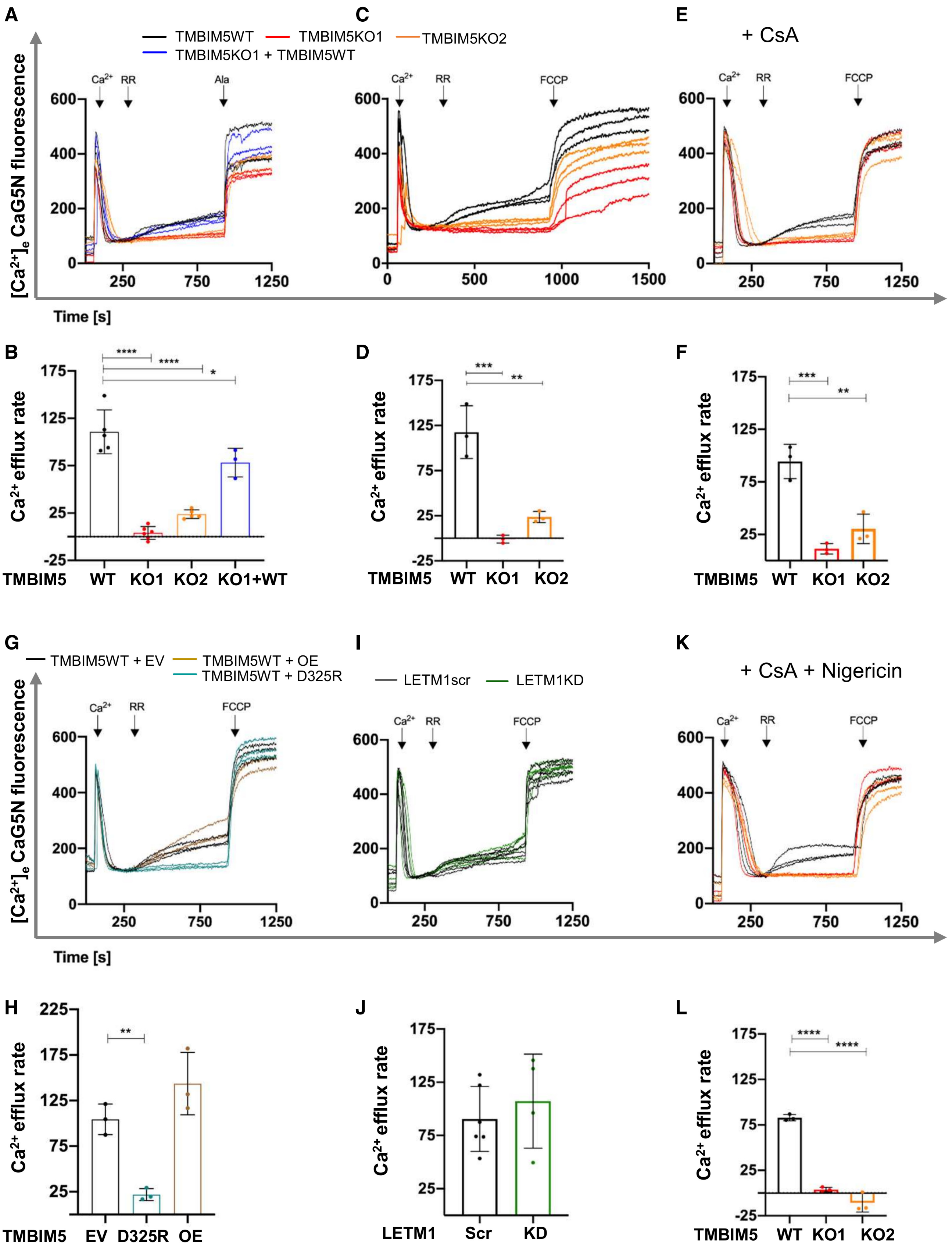
A
C
E
TMBIM5WT
TMBIM5KO1
TMBIM5KO2
TMBIM5KO1 + TMBIM5WT
+ CsA
[Ca2+]e CaG5N fluorescence
Time [s]
Ca2+
RR
Ala
FCCP
B
D
F
Ca2+ efflux rate
TMBIM5 WT KO1 KO2 KO1+WT
TMBIM5 WT KO1 KO2
TMBIM5 WT KO1 KO2
G
I
K
TMBIM5WT + EV
TMBIM5WT + OE
TMBIM5WT + D325R
LETM1scr
LETM1KD
+ CsA + Nigericin
H
J
L
TMBIM5 EV D325R OE
LETM1 Scr KD
TMBIM5 WT KO1 KO2

Figure 4.

Figure 4. TMBIM5 controls Na^+-independent Ca^{2+} release.

A–L Ca^{2+} uptake/release dynamics are shown as extramitochondrial Ca^{2+} changes of fluorescence intensities of Calcium Green 5N (Ca^{2+}-5N; 0.24 μM). Experiments conducted in permeabilized HEK293 TMBIM5WT, TMBIM5KO (A–H and K, L), TMBIM5KO + TMBIM5WT cells (A, B), WT overexpressing an empty vector (EV), or TMBIM5WT (OE) and TMBIM5KO overexpressing TMBIM5D325R (G, H), and HEK293 LETM1scr and LETM1KD cells (I, J). CGP37157 (2 μM) present in (A–L). Ca^{2+} (10 μM), RR (0.2 μM), and FCCP (2 μM) or alamethicin (2.5 μM) were added when indicated. Cyclosporin A (CsA) was added 2 min before measurements in (E, F and K, L), and nigericin 1 min before measurements (K, L). Quantification of Ca^{2+} release rates from three (D, H and F–L) and three to six (B, J) independent experiments (biological replicates; *t*: 300–920 s). Data are the mean ± SD and statistical analysis: One-Way ANOVA with Bonferroni correction (*$P < 0.05$, **$P < 0.01$, ***$P < 0.001$, ****$P < 0.0001$). See also Appendix Fig S3 for quantification of Ca^{2+} uptake.

Source data are available online for this figure.

Thapsigargin-mobilized Ca^{2+} induces PTP opening in TMBIM5KO cells

The similar vigorous Ca^{2+} uptake by TMBIM5KO and WT mitochondria but unequal Ca^{2+} release, unless alamethicin or FCCP was used, raised the intriguing question of the fate of intramitochondrial Ca^{2+}. To exclude the ER as a Ca^{2+} sink and deplete ER stores, we assessed Ca^{2+} uptake/release in permeabilized cells using measurement media containing the SERCA pump inhibitor thapsigargin (Tg). TMBIM5WT mitochondria behaved as in the absence of Tg (Fig EV5A and B). Ca^{2+} uptake was comparable in TMBIM5WT and TMBIM5KO2, while somewhat slowed in TMBIM5KO1 (Appendix Fig S3F). By contrast, TMBIM5KO1 and TMBIM5KO2 mitochondria, which were unable to release Ca^{2+} in absence of Tg, showed RR-induced Ca^{2+} efflux rates that were 4–6 times higher than those of TMBIM5WT. The Ca^{2+} efflux levels seemed saturated, as they almost reached those of total Ca^{2+} release after FCCP addition (Fig EV5A and B). These drastic effects of Tg on mitochondrial RR-induced Ca^{2+} efflux when TMBIM5 was deleted and NCLX-inhibited, suggesting stimulation of the CHE or opening of the PTP, both possibly caused by increased matrix Ca^{2+} load. Consistent with PTP opening (Beghi & Giussani, 2018), Ca^{2+} release was accompanied by significant depolarization of TMBIM5KO but not TMBIM5WT mitochondria as indicated by the membrane potential dye TMRM (Fig EV5C and D). To verify Ca^{2+} sensitivity of PTP opening, the total free Ca^{2+} load tolerated by TMBIM5KO mitochondria was assessed by Ca^{2+} retention capacity (CRC) assays. TMBIM5WT mitochondria exposed to Tg and CGP37157 tolerated 5 Ca^{2+} pulses, corresponding to 25 μM Ca^{2+} before PTP opening (Fig EV5E), whereas TMBIM5KO1 only tolerated three Ca^{2+} pulses, corresponding to 15 μM Ca^{2+} (Fig EV5F). PTP desensitization with CsA delayed the onset of pore opening by increasing the Ca^{2+} retention capacity in TMBIM5KO to a similar level as in TMBIM5WT mitochondria (Fig EV5E and F). Without blocking NCLX, the mitochondrial CRCs were comparable for TMBIM5WT and TMBIM5KO1 (Appendix Fig S6A and B). Consistent with the role of PTP opening as a Ca^{2+} release pathway, the addition of CsA and ADP prevented excess Ca^{2+} release from TMBIM5KO mitochondria (Fig EV5G and H). In Hela cells, Tg similarly triggered significantly increased Ca^{2+} release from TMBIM5KO mitochondria, reaching the level of total release by FCCP; Ca^{2+} release from TMBIM5WT mitochondria was to some extent more vigorous than in HEK293 cells (Fig EV3E and F). Consistent with increased sensitization to Ca^{2+}-induced PTP opening when NCLX is inhibited (Luongo *et al*, 2017), data suggest

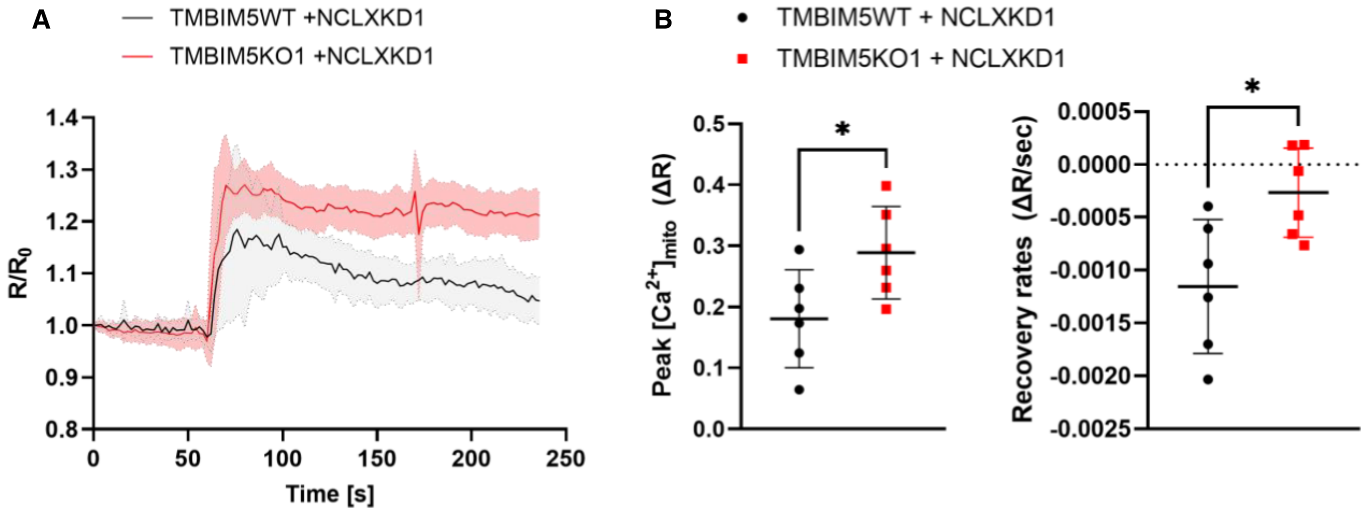

Figure 5. TMBIM5KO reduces mitochondrial Ca^{2+} ($[Ca^{2+}]_{mit}$) responses evoked by Ca^{2+}-mobilizing agonists.

A 4mtD3cpv ratio fluorescence changes (R/R0) evoked by 50 μM NaATP and 100 μM Carbachol (at time 60 seconds) in HEK293 cells depleted of NCLX and lacking or not TMBIM5.

B Statistical evaluation of the $[Ca^{2+}]_{mit}$ response amplitude (left) and recovery rates (right). Data are mean ± SD of six separate recordings with 10–14 cells each in three independent experiments (biological replicates), *$P < 0.05$, unpaired two-tailed Student's *t*-test.

Source data are available online for this figure.

© 2022 The Authors

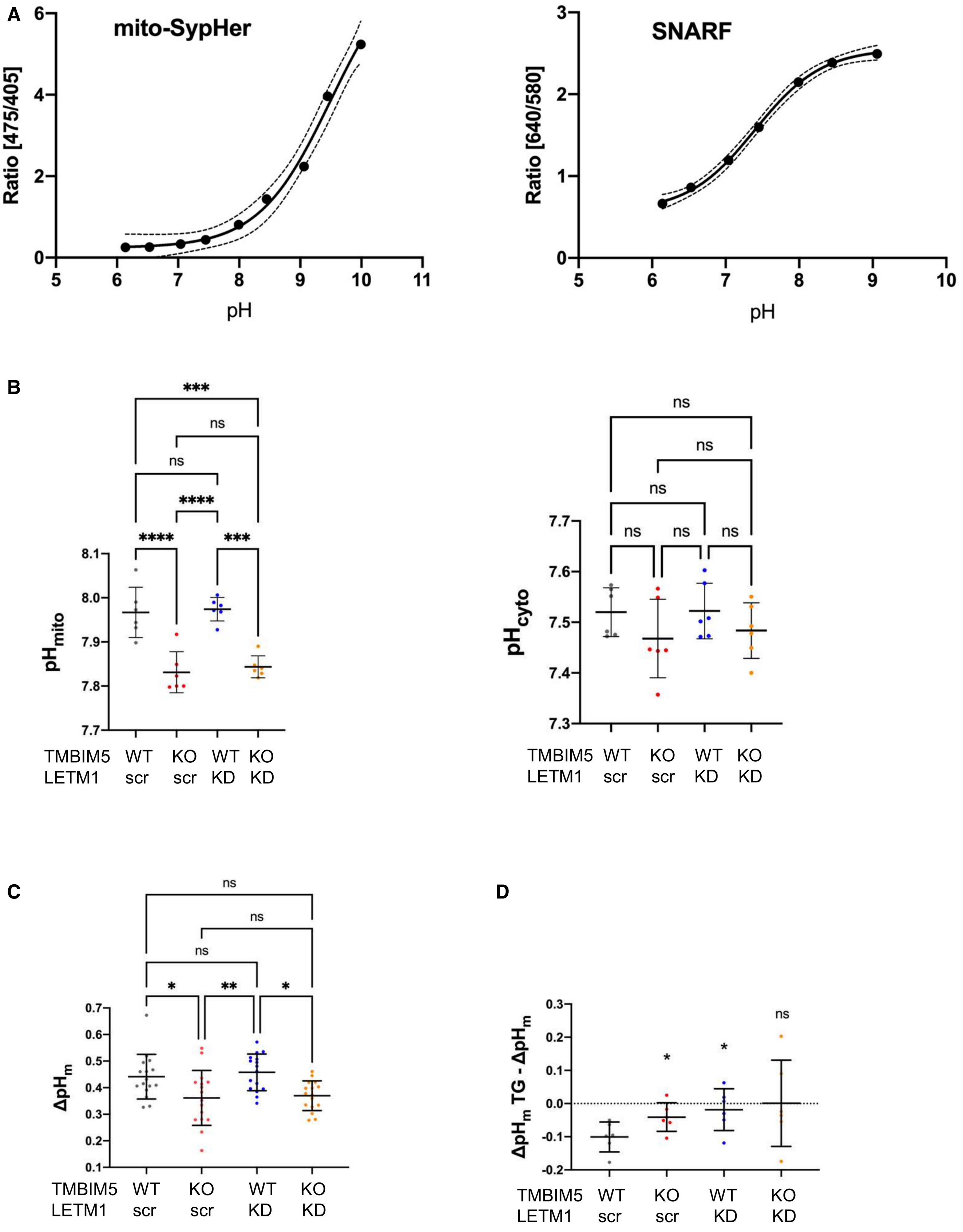
A
mito-SypHer
Ratio [475/405]
pH
SNARF
Ratio [640/580]
pH
B
pH$_{mito}$
pH$_{cyto}$
TMBIM5 WT KO WT KO
LETM1 scr scr KD KD
C
ΔpH$_m$
D
ΔpH$_m$ TG - ΔpH$_m$
ns
TMBIM5 WT KO WT KO
LETM1 scr scr KD KD

Figure 6.

Figure 6. TMBIM5 depletion alters mitochondrial pH and ΔpH.

A *In situ* calibration of mitoSypHer (left) and SNARF (right) in HEK293 cells. Data are the average of 120 cells from three independent experiments (biological replicates).

B Average resting pH_{mito} (left) and pH_{cyto} (right) of WT and TMBIM5KO cells expressing the indicated shRNA, measured by high-throughput ratio fluorescence imaging. Data are the mean ± SD of 20–60 individual cells in duplicates from three independent experiments (biological replicates). $^{****}P < 0.0001$; $^{***}P < 0.0005$; NS, not significant, one-way ANOVA.

C Averaged mitochondrial pH gradient ($\Delta pHm = pH_{mito} - pH_{cyto}$) of the indicated cell lines in resting conditions. Data are the mean ± SD of 10–15 cells of 2–4 images, in duplicates from three independent experiments (biological replicates). $^{**}P < 0.01$;$^{*}P < 0.05$; NS, not significant, one-way ANOVA.

D Effect of Tg (1 μM for 10 min, right) on the ΔpHm of the indicated cell lines. Data are the mean ± SD of 10–15 cells of 2–4 images, in duplicates from three independent experiments (biological replicates). $^{*}P < 0.05$ vs. WT Ctrl, NS, not significant, Student's *T*-test.

Source data are available online for this figure.

that NCLX-inhibited TMBIM5KO mitochondria released Ca^{2+} via the PTP in presence of Tg, which possibly increases the Ca^{2+} load by inducing higher Ca^{2+} uptake rates or mobilizing an additional source of matrix Ca^{2+}, and lowers the threshold for tolerated Ca^{2+}, thus reducing Ca^{2+} buffering capacity.

Since both NCLX and TMBIM5 regulate mitochondrial Ca^{2+} release and therefore Ca^{2+}-dependent activities, we assessed whether loss of TMBIM5 altered the pH gradient across the inner mitochondrial membrane, ΔpH_m, which contributes to the proton-motive force. To determine ΔpH_m, we measured the mitochondrial and cytosolic pH by high-throughput ratio imaging using the matrix-targeted pH-sensitive probe mitoSypHer and the cytosolic pH dye SNARF-1. *In situ* calibration of mitoSypHer and SNARF confirmed that the two probes reliably report pH changes in the physiological range (Fig 6A). Simultaneous SypHer and SNARF recordings in 200 cells per condition revealed that regardless of LETM1 depletion pH_{mito} was significantly lower in TMBIM5KO while pH_{cyto} was only marginally decreased (Fig 6B). Consequently, ΔpH_m was significantly reduced in TMBIM5KO, regardless of LETM1 depletion with shRNA (Fig 6C). The steady-state pH_{mito} and ΔpH_m of quiescent cells are thus reduced by TMBIM5 ablation.

Mitochondria acidify during cytosolic Ca^{2+} elevations as the acid generated by the activity of the plasma membrane Ca^{2+} ATPase PMCA is transmitted to the mitochondrial matrix (Poburko *et al*, 2011). To test whether the lack of TMBIM5 and NCLX impacts this Ca^{2+}-dependent acidification, we measured pH_{mito} and pH_{cyto} in cells exposed to Tg. The sustained Ca^{2+} elevation induced by Tg was associated with a significant decrease in ΔpH_m that was not observed in TMBIM5KO or in LETM1KD (Fig 6D). These data indicate that TMBIM5 ablation and LETM1 depletion both hinder the dynamic, Ca^{2+}-dependent regulation of the mitochondrial proton gradient.

Purified reconstituted TMBIM5 transports Ca^{2+}

To assess the mechanism and selectivity of TMBIM5-dependent in cation transport we produced purified TMBIM5 for reconstitution studies. Codon-optimized hTMBIM5 cDNA (Appendix Fig S7A) was cloned in pH6EX3 (Galluccio *et al*, 2013) and the recombinant construct served to transform *Escherichia coli* Rosetta cells. Protein synthesis was induced during exponential growth (OD ~ 0.8–1) by setting the temperature to 37°C and adding 0.4 mM IPTG. After 2 h TMBIM5 was overexpressed in the insoluble fraction (Appendix Fig S7B). The protein was purified by Ni-chelating chromatography and reconstituted in proteoliposomes for *in vitro* Ca^{2+} transport activity assays (see Materials and Methods). The incorporation of TMBIM5 in proteoliposomes was verified by immunoblot analysis (Fig 7A). In assays using Ca^{2+} or H^+ sensitive dyes (Fig 7B), reconstituted TMBIM5 mediated Ca^{2+} fluxes in a pH-dependent manner (Fig 7C–E), with sizable activity at pH 6.5 and pH 7.0 (Fig 7C and D), and repressed fluxes at pH 8.0 (Fig 7E). To confirm the involvement of H^+ in the transport cycle, we measured H^+ flux using the pH-sensitive dye pyranine (Fig 7F). Remarkably, alkalinization of the internal compartment of proteoliposomes detected by the increase in pyranine fluorescence indicated H^+ flux toward the external compartment induced by Ca^{2+} addition, i.e., concomitant to the inwardly directed Ca^{2+} flux (Fig 7D). To further investigate the pH dependence of the CHE activity mediated by TMBIM5, a H^+ gradient was created across proteoliposome membrane by adding Ca^{2+} in acidic or alkaline buffers (Fig 7G). According to the proposed CHE activity, an inwardly directed proton gradient impaired Ca^{2+} uptake (light green trace, Fig 7G); on the contrary, an outwardly directed H^+ gradient stimulated Ca^{2+} uptake (dark green trace, Fig 7G) with respect to the control, i.e., absence of proton gradient (red trace, Fig 7G). As a further proof of the H^+ involvement in the transport cycle, intraliposomal alkalinization was created by inducing H^+ efflux from proteoliposomes by preincubation with nigericin in the presence of external K^+ (Fig 7H, orange trace). In this condition, Ca^{2+} uptake was depressed in agreement with all the previous data.

Discussion

The role and selectivity of LETM1 as an ion transporter/channel have not been univocally assessed. The open questions remained whether it transports K^+ or Ca^{2+} and whether it operates as an exchanger or rather as a component of the transport system. The work by Shao *et al* (2016) showed that purified LETM1 oligomerizes into a high molecular weight complex of > 404 kDa, which forms a central cavity that undergoes pH-dependent conformational changes. In line with other reports, they proposed that LETM1 is a mitochondrial CHE (Jiang *et al*, 2013; Doonan *et al*, 2014). However, other studies clearly demonstrated a key role of LETM1 in mitochondrial K^+ transport (Nowikovsky *et al*, 2012; Hashimi *et al*, 2013; Austin *et al*, 2017). The unresolved identity of the mitochondrial CHE and the controversy on LETM1 motivated us to further search for LETM1 interactors that could functionally cooperate with LETM1 in mitochondrial K^+ and/or Ca^{2+} efflux.

To address the relatively low mitochondrial protein yield from mammalian cell cultures, we developed a miniaturized proteomic approach that was based on the AP-MS protocol first described by

 © 2022 The Authors

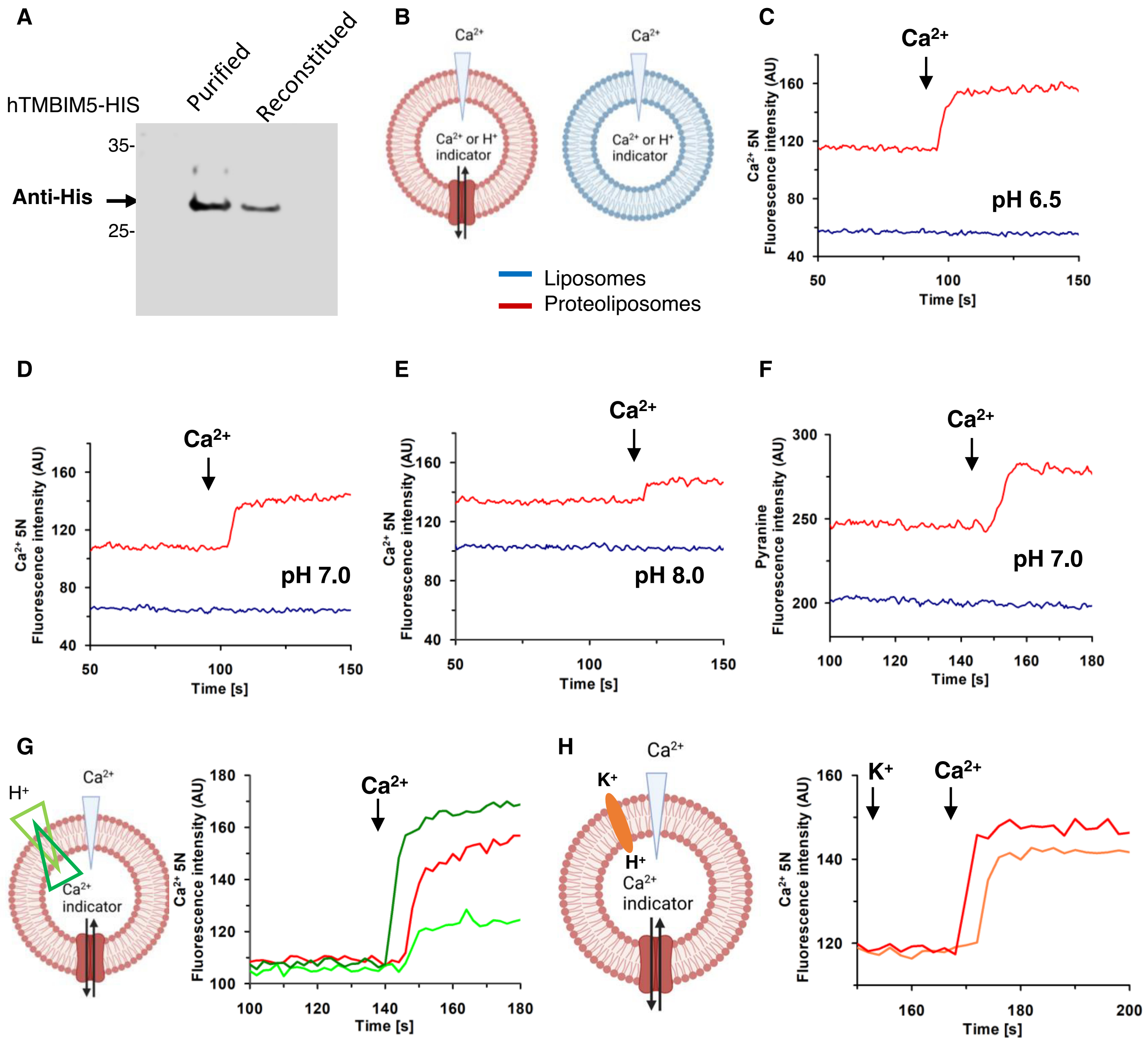

Figure 7. TMBIM5 proteoliposomes mediate Ca^{2+} and Ca^{2+}-dependent H^{+} transport.

A Western blot analysis of purified and reconstituted hTMBIM5 for evaluating the incorporation of hTMBIM5 into proteoliposomes prepared as described in Materials and Methods.

B Sketch illustrating the reconstitution of hTMBIM5 in proteoliposomes (red), and the empty liposomes (blue) prepared with Ca^{2+}-5N or pyranine for (C–F). Created with Biorender.com.

C–F Transport of Ca^{2+} by hTMBIM5 reconstituted in proteoliposomes containing 10 μM Ca^{2+}-5N at the pH indicated in the panels (C–E) or 20 μM pyranine at pH 7.0 (F). After reconstitution, the fluorescence measurement was started by diluting 200 μl proteoliposomes (red trace) up to 3 ml with transport buffer prepared as described in Materials and Methods at the indicated pH (C, E) or at pH 7.0 (D, F). After 100 s, as indicated by the arrow, 7 mM Ca^{2+} was added to the sample and fluorescence change was recorded. As a control, the same measurement was performed by diluting 200 μl liposomes (without incorporated protein, blue trace) up to 3 ml with the same transport buffer. See also Appendix Fig S7 for TMBIM5 optimization, induction, and structure overview.

G, H Transport of Ca^{2+} by hTMBIM5 reconstituted in proteoliposomes containing 10 μM Ca^{2+}-5N at pH 7.0. recorded as in (D). (G) and (H) left: sketch illustrating the assay. In (G), after reconstitution 200 μl proteoliposomes were diluted up to 3 ml with transport buffer prepared as described in Materials and Methods. Delta pH was generated adding Ca^{2+} at pH 6.0 (light green trace), 7.0 (red trace), or 8.0 (deep green trace). In (H), after reconstitution 200 μl proteoliposomes were diluted up to 3 ml with transport buffer prepared as described in Materials and Methods in the presence of 20 mM K^{+} and ethanol (red trace) or 10 μM nigericin (orange blue) to generate delta pH. Fluorescence intensity is indicated as Arbitrary Units (AU). Results are representative of three independent experiments (biological replicates).

Source data are available online for this figure.

Glatter *et al* (2009). This powerful method has been extensively used in large-scale protein interaction network studies (Pichlmair *et al*, 2012; Giambruno *et al*, 2013; Blomen *et al*, 2015; Skucha *et al*, 2018). Our adaptation for low quantities of input material was initially validated using the MCU interactome as a model and then applied to LETM1. Among the most promising identified interactors of LETM1, we focused on TMBIM5.

Our study suggests a physical interaction of LETM1 and TMBIM5 involved in K^+/H^+ exchange, which was supported by decreased LETM1 and LETM1-containing high molecular complexes in TMBIM5KO. Post-translational modifications for proteolytic degradation could also occur since gene expression was not changed (Appendix Fig S8A). Blocking the proteasomal degradation of cytosolic and organellar proteins with MG-132 did not greatly accumulate LETM1 protein levels, but comparing the LETM1 turnover rate in TMBIM5KO vs. WT indicated a relatively increased turnover rate in TMBIM5KO (Appendix Fig S8B). However, as shown in Patron *et al* (2022), TMBIM5 binds to and inhibits m-AAA proteases, raising the question of whether a higher protease activity under TMBIM5KO may initiate LETM1 degradation. Decreased levels of both LETM1 and TMBIM5 led to decreased K^+ transport. Mitochondrial Ca^{2+} overload, reduced LETM1 levels, or loss of protein–protein interaction could be the reason for lower KHE in TMBIM5KO. Interestingly, the pH- and Ca^{2+}-binding-sensitive $TMBIM5^{D325R}$ mutant had no effect on K^+ transport, suggesting that the pH-sensing or Ca^{2+}-binding function of TMBIM5 is not relevant for K^+ transport. Comparison of the roles of TMBIM5 and LETM1 in mitochondrial Ca^{2+} efflux clearly showed that LETM1 is not required for CHE activity. Using permeabilized cells, we show that in contrast to LETM1, loss of TMBIM5 abrogated the function of CHE, which was restored by re-expression of TMBIM5. Furthermore, monitoring of Ca^{2+} mobilization in intact cells confirmed that Na^+-independent Ca^{2+} release was dependent on TMBIM5. Taking advantage of *in vitro* assays, we assessed Ca^{2+} transport of TMBIM5 at different pH, or applying concomitantly with the Ca^{2+} an additional inward or outward H^+ gradient. Independent of any interaction partner or protein complex, reconstituted TMBIM5 was able to transport Ca^{2+} across proteoliposomes in a pH-dependent manner and to drive Ca^{2+}-dependent H^+ transport. Thus, based on the consistency between cellular and cell-free activity of TMBIM5 in Na^+-independent and pH-dependent mitochondrial Ca^{2+} translocation, we have identified TMBIM5 as the long-sought mitochondrial CHE. TMBIM5 has no mitochondrial homolog in *Saccharomyces cerevisiae*, which lacks a mitochondrial Ca^{2+} uptake pathway. The TMBIM5 structure predicted by AlphaFold (Jumper *et al*, 2021) shows a typical fold of membrane proteins with transport function with eight transmembrane segments and a long unresolved extra membrane domain (Appendix Fig S7C).

As previously shown (Oka *et al*, 2008) and confirmed here, loss of TMBIM5 affects mitochondrial morphology. Our data additionally demonstrate that the morphological alterations are matched by the reduced respiratory capacity that becomes evident with galactose as a substrate. The basis of this may reside in perturbation of Ca^{2+} homeostasis leading to excessive Ca^{2+} accumulation and possible alterations of K^+ homeostasis linked to secondary effects on LETM1. Thus, our findings link mitochondrial dysfunction to cation deregulation and provide a solid molecular framework for future studies.

Using high-throughput ratio imaging, we concurrently measured pH_{mito} and pH_{cyto} in individual cells to determine the pH component of the proton-motive force, ΔpH_m. ΔpH_m averaged 0.44 ± 0.08 pH units in WT HEK293 cells, a value is remarkably similar to the ΔpH_m of 0.46 pH units previously reported in HeLa cells whose pH_{mito} and pH_{cyto} were more acidic, possibly reflecting their higher glycolytic metabolism (Poburko *et al*, 2011). The matrix pH of HEK293 cells was significantly reduced by TMBIM5 ablation, while only a marginal decrease in cytosolic pH was observed. Consequently, the mitochondrial pH gradient, ΔpH_m, decreased by 0.08 pH units in cells lacking TMBIM5, a significant loss in proton-motive force. This confirms that the loss of TMBIM5 impairs mitochondrial bioenergetics, in line with the decreased oxygen consumption of TMBIM5KD cells observed in galactose media (Fig EV2E). Interestingly, both TMBIM5 ablation and LETM1 silencing prevented the dynamic equilibration of H^+ ions across the inner mitochondrial membrane during Ca^{2+} elevations, consistent with the CHE and KHE activity of these proteins.

Tg revealed severe implications of the lack of a functional CHE also on the permeability transition when Na^+-dependent Ca^{2+} efflux is concomitantly blocked, consistent with the modulatory effect of Tg on shifting the ratio between bound and free Ca^{2+} toward free Ca^{2+} (Korge & Weiss, 1999). Reduced levels of Sirt3, which is responsible for the deacetylation of CypD, a key PTP sensitizer (Sambri *et al*, 2020) may contribute to permeability transition. The hypersensitivity of Ca^{2+}-induced PTP opening also correlates with the observed cristae disorganization, OPA1 cleavage pattern, and OMA1 activation. Consistent with the role of OPA1 in cristae architecture, its interaction with the ATP synthase, the feedback loop between cristae structures and ATP synthase dimerization (Quintana-Cabrera *et al*, 2018), and the fact that the PTP may be formed by ATP dimers (Carrer *et al*, 2021), OPA1 changes under TMBIM5 ablation may explain an increased predisposition to cell death in TMBIM5KO cells exposed to Tg.

While this work was in revision, (Zhang *et al*, 2022) published a study, which is consistent with our data on TMBIM5KO perturbing LETM1-mediated KHE. Yet, the study is at odds with our conclusion that TMBIM5 interacts with LETM1. The conclusion of Zhang *et al* that TMBIM5 and LETM1 do not interact is based on (i) experiments that examined the interaction between the active processed TMBIM5 (26 kDa) tagged with GFP (27 kDa) and LETM1 (data not shown) and (ii) a BN blot from isolated mitochondria that were solubilized with 5% digitonin, which is an unusually high detergent/protein ratio (Guo *et al*, 2019a; Cogliati *et al*, 2021; Moreno-Justicia *et al*, 2022). Another discrepancy is Ca^{2+} transport, which in this study was performed in very different experimental settings, such as no depletion of NCLX to unmask the activity of the CHE (Zhang *et al*, 2022). Moreover, results obtained by overexpression of TMBIM5 with a tag that is larger than the protein may interfere with function. Data by Patron *et al* (2022) published while our revised paper was under review support our findings that TMBIM5 is the mitochondrial CHE. One discrepancy is that we do observe a more acidic matrix pH in TMBIM5 KO cells while Patron *et al*, and Zhang *et al*, reported an alkalinization. The direction of transport catalyzed by TMBIM5 cannot be directly inferred from changes in steady-state matrix pH, as the latter primarily reflects mitochondrial respiration and H^+ pumping to the intermembrane space, and secondary equilibration of substrates and phosphate (Poburko *et al*, 2011). The

© 2022 The Authors

matrix acidification that we report was tied to a decreased ΔpH_m and oxygen consumption, consistent with a switch from oxidative to glycolytic metabolism in TMBIM5KO cells. The matrix alkalinization reported in the two other studies might reflect an opposite switch, from glycolytic to oxidative, which would be consistent with the acidic matrix pH in HeLa WT cells reported by Patron *et al* (2022). A comparison with the data of Zhang *et al* (2022) is not easy, as in this paper the fluorescence changes were not calibrated against pH.

In conclusion, we demonstrated in cell-free and cell-culture models that TMBIM5 is the mitochondrial CHE. Results on TMBIM5-mediated Ca^{2+} transport shown in Patron *et al* (2022), confirmed our finding. Although being best suited for detailed and complementary studies of mitochondrial K^+/H^+ and Ca^{2+}/H^+ exchange, using cell-free and cellular systems is limited by not revealing the tissue-specific significance of TMBIM5 as a mitochondrial cation exchanger. However, this work opens the door for significant further study in an organismal context, which can be used to accurately determine the importance of the interaction. In view of the established involvement of LETM1 in both KHE and CHE activity, the identification of the LETM1 partner TMBIM5 is also a major step forward in resolving current controversies on their relative role in mitochondrial Ca^{2+} and K^+ homeostasis. While paving the way to further explore the molecular determinants of the interaction and interdependencies between LETM1 and TMBIM5, this study has demonstrated that TMBIM5 is necessary to maintain the KHE machinery, and its interaction with LETM1 fulfills a physiological role in the cell and in maintaining Ca^{2+} balance. Moreover, it has also identified a TMBIM5 mutant that allows discrimination between the functions of TMBIM5 in K^+ and Ca^{2+} transport. Further investigation is needed to understand how LETM1 and TMBIM5 link mitochondrial K^+ and Ca^{2+} cycles and to shed more light on the regulatory mechanism of LETM1 and its interaction partners in maintaining mitochondrial ion homeostasis.

Materials and Methods

Reagents

All reagents used in this study were from Sigma–Aldrich unless otherwise indicated.

Antibiotics: normocin, blasticidin, hygromycin, puromycin, and doxycycline were from Invivogen (San Diego, CA). Restriction endonucleases and specific reagents for cloning, pierce BCA protein assay kit, glutaraldehyde, lead citrate, propylene oxide, and osmium tetroxide were from Merck (Darmstadt, Germany), protein G magnetic beads from NEB (#S1430s), ProtA/G agarose and DMEM (#41966-029) from Thermo Fisher Scientific (NativeMark™ #LC0725) NativePAGE™ (3–12% Bis-Tris Protein, #BN1001), Turbofect, Lipofectamine 2000, Ca^{2+} Green 5N, and MitoTracker™ Green FM (#M7514) from Invitrogen, Streptactin beads from IBA lifesciences. Bradford was from BioRad, proteinase inhibitor from Roche (Basel, Switzerland), C12E8 from TCI Europe, TMRM from molecular probes, glycid ether 100 from Serva (Heidelberg, Germany). Fetal bovine serum (FBS) and pen/strep were from Gibco. Mycoplasma test kit was from MycoAlert Lonza kit, SNARF/AM (C1272; Invitrogen). The working concentration of ruthenium red was calculated with Lambert–Beer law, A = 533 nm, I = 1 cm, Ɛ = 65,000.

Cell culture

HEK293 Flp-In TRex (Invitrogen), HeLa (Austin *et al*, 2017), and HEK293 (ATCC CRL-1573) cells were maintained in DMEM supplemented with FBS (10% v/v), and penicillin/streptomycin (pen/strep) (1%). Cells were cultured in an incubator set to 37°C and 5% CO_2 and splitted when reaching confluency of ~70–90%, and regularly tested for mycoplasma.

Generation of knockdown, knockout, and transgene expression cells

shRNA constructs for LETM1 and nontarget control (scr) were obtained from Origene Technologies (Rockville, MD) as described in Austin *et al* (2017). TMBIM5KD cells were generated using the human shRNA plasmid kit (Origene, TR315671B) with the shRNA construct 1 (GGTCTTGGAGCATTCTGCTACTATGGCTT) and construct 2 (GCCATAGCAATCAGCAGAACGCCTGTTCT and GGTCCTCTTCT CATCAGAGCTGCATGGTA). NCLX stable knockdown was achieved by lentiviral transduction as in Grandits *et al* (2021), shRNA constructs for NCLX were TRCN0000005046 targeting CCGGGTATCTTC TAATACCAA (KD1) and TRCN0000005048 targeting GTGTGCTT TGTGTGCTGCTAA (KD2) from Sigma MISSION©.

TMBIM5KO cells were generated by the Protein Technologies Facility at Vienna BioCenter Core Facilities, member of the Vienna BioCenter, Austria (www.viennabiocenter.org/facilities/). Four gRNAs targeting TMBIM5 were designed using CRISPOR tool (crispor.tefor.net). gRNAs were selected primarily on the criterium of their specificity (at least three mismatches with at least one in the seed region to any off-target) and on predicted activity according to Doench score. Guide 1: CCAAAACAAGAATTGGGATC (targeting exon 3), guide 2: GCATTGTGCTACTATGGCTT (targeting exon 4), guide 3: CAGCCATTGATTCTTCGTGA (targeting exon 2) and guide 4: GGCTCCTCTGACAATATTA (targeting exon 7). Targeting sequences were introduced into pX459 Cas9-p2A-puro plasmid (Addgene 48139) via BbsI cloning. Plasmids (3 μg) were introduced into HEK293 cells (1×10^6) by electroporation with Neon electroporator (Thermo Fisher Scientific) according to the manufacturer's protocol. Twenty-four hours post electroporation cells were selected with puromycin (4 μg/ml) and 72 h later collected and lysed for genotyping. Editing efficiency was confirmed with TIDE algorithm (https://tide.deskgen.com/) based on chromatogram analysis with WT HEK293 PCR product used as a reference. Guide 2 (GCATTGTGCTACTATGGCTT) was selected for performing the KO in HEK293 and HeLa cells based on its highest activity (59.7%) and cloned into an in-house template vector p31 vector, which contains T7 promoter, BbsI cloning sites, optimized gRNA scaffold and DraI restriction site used for template linearization according to a standard protocol (Jinek *et al*, 2012) with minor modifications, as described in supplementary material in (Pinto *et al*, 2020). Resulting gRNA transcription was performed with HiScribe T7 High Yield RNA Synthesis Kit (NEB) according to the manufacturer's protocol and gRNA was purified and verified for concentration and RNA integrity. Twelve microgram of gRNA premixed with 5 μg Cas9 protein (2 × NLS) in Cas9 buffer (20 mM HEPES pH 7.5, 150 mM KCl,

0.5 mM DTT, 0.1 mM EDTA) were used for electroporation of 70–80% confluent cells (1 × 10^6). Cas9 protein was produced in-house according to Jinek *et al* (2012) and supplementary material in Pinto *et al* (2020). Electroporated cells were cultured in DMEM supplemented with 10% FCS and L-Gln. Normocin was added after approximately 2 h, and after 24 h genotyping was performed to confirm editing. Editing was assessed by PCR and sequencing on three occasions: first to select the most efficient editing of the cloned different guide RNAs, second to evaluate the efficiency of the pool editing within a batch electroporated with selected *in vitro* transcribed gRNA and recombinant Cas9, and finally genotyping of single clone colonies. Therefore, 24 h after electroporation, a serial dilution of the culture to 0.5 cells/well was prepared for single clones. Confluent clones were collected for genotyping by PCR and sequencing. The frameshift deletion of the selected clones: HEK293 clones: (KO1) IIF1: −5/−4/−1; (KO2) IE12: −8/−5/−4, and HeLa clone IIIF3: −5 (KO).

LETM1 scramble and LETM1KD in HeLa cells were described (Austin *et al*, 2017), generated in HEK293, and generated as in Austin *et al* (2017).

Unless other specified, Turbofect was used for cell transfection as per the manufacturer's instructions. After 48 h, transfection was selected using selection antibiotics as listed: puromycin (1 μg/ml), hygromycin (260 μg/ml), blasticidin S (38 μg/ml).

Lentiviral particles were produced in Lentix cells transfected with a mix of 3 μg pMD2.G, 6 μg psPAX2, and 6 μg pLKO.1-puro shRNA diluted in Opti-MEM® (Life Technologies). Virus-containing medium was collected at 48 and 72 h post-transfection, filtered through a 0.45 μm and lentiviral particles were delivered to 4 × 10^5 HEK293 cells along with 5 μg/μl polybrene. The selection of transduced cells was done with puromycin (1 μg/ml).

Cell lines with stable inducible transgene expression of SH-tagged LETM1, MCU- or GFP were generated by co-transfection of the plasmids with the Flp recombinase expression plasmid pOG44 (Life Technologies) into HEK293 Flp-In-TREx cells (Rudashevskaya *et al*, 2013). Inducible expression was verified by western blotting. For stable expression of empty, or TMBIM5WT or TMBIM5D325R containing vector, transfected cells were selected on puromycin (2 μg/ml).

For transient MitoSypher or 4mtD3cpv expression, cells were cultured at 60% confluency onto black clear-bottom 96 well plates (655090; Greiner Bio-One™) and transfected using Lipofectamine 2000 as per the manufacturer's instructions. Cells were treated with 0.25 μl of Lipofectamine 2000 and 0.1 μg of DNA in 125 μl medium per well for 24 h, and used for imaging experiments right after.

Molecular cloning

LETM1 cDNA was amplified from pVT-U LETM1 (Nowikovsky *et al*, 2004) and subcloned into the pTO-SII-HA-GW vector, which was a kind gift from M. Gstaiger (M. Gstaiger, ETH, Zurich). The streptavidin–hemagglutinin (S-HA) tag was sub-cloned in fusion to the C-terminal end of LETM1 by Gateway recombination cloning (Invitrogen, Carlsbad, CA). Primers: *attB LETM1 forward* 5′GGGGACAA GTTTGTACAAAAAAGCAGGCTAGACTGCCATGGCGTCCAT3′, *attB LETM1 reverse* 5′GGGGACCACTTTGTACAAGAAAGCTGGGTTGCTC TTCACCTCTGCGAC3′. MCU was sub-cloned into the pTO-SII-HA-GW vector as above using the primers *attB MCU forward* 5′GGGGACAAGTTTGTACAAAAAAGCAGGCTAGGCCACCATGGCG-GCCGCCGCAGGTAG3′ *attB MCU reverse* 5′GGGGACCACTTTGTAC AAGAAAGCTGGGTTATCTTTTTCACCAATTTGTC′. The plasmid (pTO-SII-HA-GW GFP) expressing GFP with SH tag used for tandem affinity purification control was a kind gift from A. Bergthaler (CeMM, Vienna).

The human TMBIM5 cDNA was amplified by reverse-transcriptase PCR using the primers *forward* 5′TAACTCGAG TCTAGAGGG3 and *reverse* 5′TTTCTTTCTGTTGCCTCC3′ and cloned into the pcDNA3.1+ plasmid (Sigma–Aldrich) using the restriction sites *HindIII* and *Xho1*. TMBIM5D325R was generated by site-directed mutagenesis using the Q5 site-directed mutagenesis kit (Neb #E0552S) using the primers: For. 5′TATCTACATGAGAACA TTAAATATATTTATGCG3′, Rev. 5′CTCAGCATCGAGTTAATG3′ and cloned into pcDNA3.1+. All primers were from Microsynth, Balgach, Switzerland.

For proteoliposomes, codon optimization of the human TMBIM5 sequence (UniProtKB: Q9H3K2; GenPept accession no. NP_055209. 2) was designed using Genscript and increased the Codon Adaptation Index (CAI) from 0.32 to 0.97. and it was sub-cloned from pUC57 by double digestion and inserted between *Hind*III and *Xho*I restriction sites of the pH6EX3 expression vector. The resulting recombinant plasmid encodes a 6His-tagged fusion protein corresponding to the hTMBIM5 carrying the extra N-terminal sequence MSPIHHHHHHLVPRGSEA.

The MitoSypher plasmid was cloned and characterized as previously described (Poburko *et al*, 2011).

Mitochondria isolation

Cell pellets resuspended in isolation media (Austin *et al*, 2017) containing 1.7 mM proteinase inhibitor cocktail were homogenized on ice with 12 strokes at 1,600 rpm with a yellow line OST basic homogenizer and mitochondria isolated by differential centrifugation according to Frezza *et al* (2007).

Sample preparation for tandem affinity and affinity purification

Protein expression was induced with doxycycline (1 μg/ml) for 24 h in standard culture media. Cells were lysed and the bait protein purified by TAP or AP from mitochondria as in Rudashevskaya *et al* (2013) with modification. Crudely isolated mitochondria were lysed using 6-aminocarproic acid with protease inhibitors and n-Dodecyl β-D-maltoside (2% w/v) and vortexed for 30 min at 4°C. Lysates were cleared at 15,000 × *g*, 4°C for 15 min and the supernatant was quantified by Bradford assay with BSA as standard. Protein complexes were purified from 2 mg crude mitochondrial input with Streptactin (IBA, Göttingen, Germany) beads. Washing steps were performed in a scaled volume of AP buffer, thrice with detergent, twice without, and then eluted with biotin (Alfa-Aesar, Ward Hill, MA). Protein complexes were reduced, alkylated, and digested with trypsin as described (Rudashevskaya *et al*, 2013). Peptides were desalted and concentrated by reversed-phase tips (Rappsilber *et al*, 2007) and reconstituted in formic acid (5%) for LC–MS analysis.

Reversed-phase liquid chromatography–mass spectrometry, data analysis, and data filtering

Mass spectrometry analysis was performed on a hybrid linear trap quadrupole (LTQ) Orbitrap Velos mass spectrometer (ThermoFisher

© 2022 The Authors

Scientific, Waltham, MA, USA) using the Xcalibur software (version 2.1.0). The instrument was coupled to an Agilent 1200 HPLC nanoflow system with a dual pump, one precolumn, and one analytical column (Agilent Biotechnologies, Palo Alto, CA, USA) via a nanoelectrospray ion source with a liquid junction (Proxeon, Odense, Denmark). The peptide mixtures were automatically loaded from the thermostatted autosampler (4°C) onto a trap column (Zorbax 300SB-C18 5 µm, 5 × 0.3 mm, Agilent Biotechnologies, Palo Alto, CA, USA) with the binary pump solvent comprised of 0.1% trifluoracetic acid (TFA) in water at a flow rate of 45 µl/min. The peptides were eluted by back-flushing from the trap column onto a 16 cm fused silica analytical column with an inner diameter of 50 µm packed with C18 reversed-phase material (ReproSil-Pur 120 C18-AQ, 3 µm, Dr. Maisch GmbH, Ammerbuch-Entringen, DE). Solvents for peptide separation were composed of 0.4% formic acid (FA) in water (solvent A) and 0.4% FA in 20% isopropanol, 70% methanol (solvent B). Multistep linear gradient elution of the peptides was achieved by a 27 min gradient ranging from 3 to 30% solvent B, followed by a 25 min gradient from 30 to 70% solvent B and, finally, a 7 min gradient from 70 to 100% solvent B at a constant flow rate of 100 nl/min. The global MS analyses were performed in a data-dependent acquisition mode. The top 15 most intense ions were selected for collision-induced dissociation (CID) at a normalized collision energy of 30%. Dynamic MS2 exclusion of selected ions for fragmentation was 60 s and a single lock mass at m/z 445.120024 (Olsen *et al*, 2005) for the siloxane Si(CH3)2O)6 was used for internal mass calibration with a target loss mass abundance of 0%. Maximal ion accumulation time allowed for MS1 was 500 ms in the C-trap, and for MS2, the ion accumulation time was 50 ms in the LTQ. Overfilling of the C-trap was prevented by automatic gain control (AGC) and set to 106 ions for a full FTMS scan and 5 × 105 ions for MSn mode. Intact peptides were detected in the Orbitrap mass analyzer at a resolution of 60,000. The signal threshold for triggering MS2 fragmentation was 2,000 ion counts. Raw data were matched to peptides and proteins using Mascot and Phenyx, with a false discovery rate of 1% at the protein level. CRAPome (v1) and SAINT (Choi *et al*, 2011) analyses were applied to all TAP or AP-MS data. GFP pulldowns were used as controls together with publically-available CRAPome data that used similar sample preparation and MS methods and instrumentation (Mellacheruvu *et al*, 2013b; Data ref: Mellacheruvu *et al*, 2013a). Common contaminants and proteins with a frequency greater than or equal to 0.1 in the CRAPome database were excluded. Proteins with a SAINT score greater than 0.97 were identified as high-confidence interactors.

Co-immunoprecipitation

Co-IPs were done with inducible LETM1-SH (used in tandem affinity and affinity purification experiments) or endogenous protein expression. HEK293 cells were washed with PBS and harvested in the co-IP buffer: 150 mM NaCl, 50 mM Tris, 2 mM EDTA, 1% IGEPAL C360, and protease inhibitor. Cell lysates were vortexed, cleared, and quantified as described above. Lysates (500 µg or 1 mg) were then incubated overnight with primary antibody as indicated. Primary antibody samples were incubated for 1 h at 4°C with ProtA agarose or ProtG magnetic beads. Beads were then washed three times with co-IP buffer then two times with PBS and eluted with 3× Laemmli buffer for SDS–PAGE and immunoblotting.

Western blotting

SDS–PAGE and immunoblotting were performed as in Austin *et al* (2017). Bradford or BCA assays were performed according to the manufacturer's protocol and blots were quantified using the BioRad Image Lab (v6.1.0), and VisionWorks softwares. Antibodies are listed in Appendix Table S1.

BN–PAGE and western blotting

Isolated mitochondria were solubilized with a final concentration of 1% digitonin (corresponding to 20 gDIG/gMITO, the titrated optimum concentration giving the same results was 1–2%) for 15 min on ice, centrifuged at 27,000 × *g* for 30 min in a Beckman Optima™ ultracentrifuge and the supernatant (corresponding to 5 µg) with G-250 Sample Additive (0.5 µl) was separated using precasted gels (NativePAGE™ 3–12% Bis-Tris Protein). Unstained Protein Standard NativeMark™ served as a marker. Protein complexes were transferred onto PVDF membranes overnight using wet blotting at 30 V. Antibodies are listed in Appendix Table S1.

Proliferation assay

Cell number was determined every 24 h using trypan blue staining. At least three independent counts were performed on each sample. Cell numbers were plotted, and data were shown as mean ± SD.

Seahorse Mito stress assay

Extracellular flux analyses were performed with the Agilent Seahorse XF24 Extracellular flux analyzer as outlined in Wilfinger *et al* (2016), with oligomycin (0.5 µM) and FCCP (0.2 µM). Carbon source as in the figure legend, (glucose 25 mM) or galactose (10 mM), all media were supplemented with sodium pyruvate (1 mM).

Light scattering assays

Light scattering experiments were adapted from previous protocols (Austin *et al*, 2017). Briefly, freshly isolated mitochondria were resuspended in isolation buffer: 200 mM Sucrose, 10 mM Mops-TRIS, 1 mM EGTA-TRIS, pH: 7.4. Antimycin A (5 µM) was used at RT to depolarize mitochondria and A123187 (1 µM) and EDTA (10 µM) to deplete matrix magnesium. Light scattering assays were conducted in a photometric 96 well plate reader (Varioscan) at RT; KOAc media (180 µl), as described in Austin *et al* (2017) was injected into 200 µg mitochondria to a total volume of 200 µl and absorbance was detected at OD_{540nm}. Quinine (0.5 mM) served to inhibit the KHE. The swelling rate was quantified by one phase decay on raw swelling data as shown, K value as rate constant. Of note, mitochondria isolated from frozen or fresh cell lysates gave comparable results.

Mitochondrial Ca^{2+} uptake/release assays

Cells (7×10^6) were permeabilized with digitonin (1.25%) in 400 µl permeabilization media PM1: KCl (130 mM), Mops-Tris pH 7.4 (10 mM) EGTA-Tris (1 mM), KPi pH.7.4 (1 mM). Permeabilization was stopped (immediately after 80–90% of the cells had become permeable to trypan blue) in 600 µl PM2: KCl (130 mM), Mops-Tris pH

7.4 (10 mM), EGTA-Tris (10 μM), KPi pH 7.4 (1 mM), and resuspended in measurement media (MM) contained sucrose (250 mM), MOPS-Tris (10 mM), EGTA-Tris (10 μM), KPi 7.4 (1 mM), sodium succinate (5 mM), and rotenone (2 μM). CGP37157 (2 μM) served to inhibit NCLX, and when indicated thapsigargin (1 μM) to block SERCA. Ca^{2+}-5N (0.24 μM) was used to record extramitochondrial Ca^{2+}, TMRM (0.33 μM) to measure the membrane potential. A bolus of $CaCl_2$ (10 μM) was applied to initiate Ca^{2+} uptake. MCU was inhibited by RR (0.2 μM). FCCP (2 μM) or alamethicin (2.5 μM) was added to induce the maximal release of total Ca^{2+} at the end of the measurement. The LS55 spectrofluorometer 211 (Perkin Elmer) was used with the following parameters: Ca^{2+} green-5N: λ_{ex} = 505 nm, λ_{em} = 530 nm, slit width: Ex-2.5 nm, Em-2.5 nm; TMRM: λ_{ex} = 546 nm, λ_{em} = 590 nm, slit width: 2.5 nm.

For Ca^{2+} recordings in intact cells, cells were plated on 25 mm coverslips coated with poly-lysine for 24 h to 60% confluency, followed by transfection with the pcDNA-4mtD3cpv plasmid with lipofectamine 2000 according to the manufacturer's instructions. Cells were treated with 0.25 μl of lipofectamine 2000 and 0.1 μg of DNA in 125 μl medium per well for 24 h prior to experiment day. For ratio imaging of 4mtD3cpv cells were excited at 430 nm through a 455DRLP dichroic and alternately imaged with 480AF30 and 535DF25 emission filters (Omega Optical) as previously described (De Marchi *et al*, 2011). Recordings were performed at 37°C in modified Ringer's buffer containing 2 mM Ca^{2+} using a combination of 50 μM NaATP (Sigma; A6419) and 100 μM carbachol (Sigma; C4382) to mobilize Ca^{2+} from ER stores.

Calcium retention capacity (CRC) experiment

CRC was performed in MM containing Ca^{2+}-5N and when indicated Tg (1 μM), CsA (1 μM), and CGP37157 (1 μM). $CaCl_2$ pulses (5 μM) were added sequentially until the opening of PTP occurred. Measurements in LS55 spectrofluorometer with the same parameters as above.

pH measurements

Prior to experiments MitoSypher expressing cells were loaded with SNARF (5 μM, 0.2% DMSO) for 30 min at room temperature in HEPES-based buffer (20 mM HEPES, 140 mM NaCl, 5 mM KCl, 1 mM $MgCl_2$, 2 mM $CaCl_2$, 10 mM glucose; with pH set to 7.4 with NaOH at 37°C), the cells were then incubated in HEPES buffer without SNARF for 20 min for de-esterification. Cells were then treated at 37°C for 10 min, with or without thapsigargin (1 μM) or with the pH calibration solutions (5 μg/ml of nigericin and 5 μM of monensin in 125 mM KCl, 20 mM NaCl, 0.5 mM $MgCl_2$, 0.2 mM EGTA, and 20 mM N-methyl-D-glutamine (for pH range 9.5–10.0), 20 mM Tris (for pH range 8.0–9.0), 20 mM HEPES (for pH range 7.0–7.5), or 20 mM MES (for pH range 5.5–6.5)). Images were acquired using the ImageXpress Micro plate reading microscope system set at 37°C with humidity and CO_2 (Molecular Devices). Using autofocusing and automated image acquisition (20× objective, 0.75 numerical aperture, in widefield mode), ratio images for MitoSypher (F405: ex1 390/22 nm, Dichroic1 415 nm, em 520/35 nm; F480: ex2 475/28 nm, Dichroic2 500 nm, em 520/35 nm), and SNARF (F580: ex 475/28 nm, Dichroic1 500 nm, em1 600/37 nm; F640: ex2 475/28 nm, Dichroic2 500 nm, em1 692/40 nm) were obtained with the Andor Zyla 4.2 camera. For each plate, a 9-points pH curve was obtained and fitted using a sigmoidal, 4PL, X is log (concentration) equation and ratios were converted to pH correspondingly.

Cell imaging

Transmission electron microscopy

Cells were fixed in glutaraldehyde (5%) phosphate buffer (0.1 M; Sigma–Aldrich, Vienna, Austria), pH 7.2, at 4°C for 2 h. Subsequently, samples were postfixed in 1% osmium tetroxide in the same buffer at 4°C for 1 h. After dehydration in an alcohol gradient series and propylene oxide, the tissue samples were embedded in glycid ether 100. Ultrathin sections were cut on a Leica ultramicrotome (Leica Ultracut S, Vienna, Austria), stained with uranyl acetate and lead citrate, and examined with a Zeiss TEM 900 electron microscope (Carl Zeiss, Oberkochen, Germany) operated at 80 kV.

Live cell imaging

5×10^4 cells/well were seeded onto poly-L-lysine coated μ-Slide 8 well plates (Ibidi, #80826). The next day mitochondria were loaded with MitoTracker™ Green FM (50 nM) for 30 min and then changed to fresh medium before they were monitored under 5% CO_2 at 37°C using an LSM880 microscope with Plan-Apochromat 63×/1.40 Oil DIC M27 lens. MTG was excited at a wavelength of 488 nm and images were processed in Adobe Photoshop CS2.

Overexpression, purification, and reconstitution in proteoliposomes of TMBIM5 for Ca^{2+} transport assays

Expression of TMBIM5 protein

To produce the 6His-TMBIM5 recombinant protein, *E. coli* Rosetta cells (Novagen) were transformed with the pH6EX3-hTMBIM5 construct. Selection of transformed colonies was performed on LB-agar plates added with ampicillin (100 μg/ml) and chloramphenicol (34 μg/ml). A colony was inoculated and cultured overnight at 37°C under rotary shaking (160 rpm). The day after, the culture was diluted 1:20 in fresh medium added with the specific antibiotics. When the optical density measured at $OD_{600\ nm}$ wavelength was 0.8–1, different IPTG concentrations (from 0.1 to 1 mM) were tested to induce protein expression except for one aliquot, grown in absence of inducer (negative control). The cultures were continued for up to 6 h at 28°C or 37°C at 160 rpm. Every 2 h, aliquots were collected and centrifuged at 3,000 × *g*, and at 4°C for 10 min; the pellets were stored at −20°C. A bacterial pellet aliquot, after thawing, was dissolved in a resuspension buffer (20 mM Hepes Tris, 200 mM NaCl pH 7.5) added with a protease inhibitor cocktail according to manufacturer instructions. The bacterial suspensions were sonicated in an ice bath for 10 min (pulse of 1 s on, and 1 s off) at 40 Watt, using a Vibracell VCX-130 sonifier. The insoluble cell fractions were analyzed by SDS–PAGE and western blotting.

Purification of hTMBIM5

hTMBIM5, overexpressed in *E. coli*, was purified by Ni-chelating chromatography. In brief, the insoluble fraction of bacterial cell lysates was firstly washed with a buffer containing Tris–HCl pH 8.0 (0.1 M). After centrifugation step (12,000 × *g* for 5 min at 4°C), pellet was resuspended with 100 mM 1,4-dithioerythritol (DTE) and then solubilized with a buffer containing urea (3.5 M), sarkosyl (0.8%), NaCl (100 mM), glycerol (5%), Tris–HCl pH 8.0 (10 mM). After solubilization, the

 © 2022 The Authors

sample was centrifuged at 12,000 × *g* for 10 min at 4°C and the supernatant was applied onto a column filled with 2 ml His select nickel affinity gel (0.5 cm diameter, 2.5 cm height) preconditioned with 8 ml of a buffer containing sarkosyl (0.1%), NaCl (200 mM), glycerol (10%), Tris–HCl pH 8.0 (20 mM). Then, 5 ml of a buffer containing Tris–HCl pH 8.0 (20 mM), glycerol (10%), NaCl (200 mM), n-Dodecyl β-D-maltoside (0.1%), and DTE (5 mM) was used to wash the column removing unbound proteins. In order to increase the purity of the recovered TMBIM5, another washing step was performed using 3 ml of the same above-described buffer added with 10 mM imidazole. Finally, TMBIM5 was eluted in 5 fractions of 1 ml, using the same above-described buffer added with 50 mM imidazole. The purified protein was eluted in a peak of 2.5 ml. The eluted protein was subjected to a buffer change for imidazole and Na^+ removal, using a PD-10 column preconditioned with a desalt buffer composed of Tris–HCl pH 8.0 (20 mM), glycerol (10%), n-Dodecyl β-D-maltoside (0.1%), and DTE (10 mM): 2.5 ml of the purified protein were loaded onto the PD10 column and collected in 3.5 ml of desalt buffer.

Reconstitution in proteoliposomes of the purified hTMBIM5

The desalted hTMBIM5 was reconstituted by removing detergent from mixed micelles of detergent, protein, and phospholipids using the batchwise method previously described for other membrane proteins (Cosco *et al*, 2020), with some modifications to increase the protein/phospholipid ratio required for fluorometric measurements (Scalise *et al*, 2020). The initial mixture contained: 25 µg of purified protein, 50 µl of 10% $C_{12}E_8$, 50 µl of 10% egg yolk phospholipids (w/v) in the form of liposomes prepared as previously described (Scalise *et al*, 2018), 20 mM Tris–HCl pH 7.0, except where differently indicated, 10 µM of Calcium Green-5N or 20 µM pyranine, in a final volume of 700 µl. The detergent was removed by incubating the reconstitution mixture with 0.5 g of the hydrophobic resin Amberlite XAD-4 for 40 min under rotatory stirring at room temperature.

Cation transport measurements by spectrofluorometric assays

The Ca^{2+} flux or the intraliposomal pH changes were monitored by measuring the fluorescence emission of Calcium Green-5N or pyranine, respectively, included inside the proteoliposomes. After reconstitution, 600 µl of proteoliposomes were passed through a Sephadex G-75 column, pre-equilibrated with Tris–HCl pH 7.0 (20 mM), except where differently indicated. Then, 200 µl proteoliposomes were diluted in 3 ml of the same buffer and incubated for 10 min in the dark prior to measurements. To start the transport assay, $CaCl_2$ (7 mM) buffered at pH 7.0, except where differently indicated, was added to proteoliposomes; the uptake of Ca^{2+} or the efflux of H^+ was measured as an increase in Calcium green-5N or pyranine fluorescence, respectively. As a control, the same measurements were performed using liposomes, i.e., vesicles without reconstituted hTMBIM5. The measurements were performed in the fluorescence spectrometer (LS55) from Perkin Elmer under rotatory stirring. The fluorescence was measured following time drive acquisition protocol with λ excitation = 506 nm and λ emission = 532 nm (slit 5/5) for Calcium Green-5 N and λ excitation = 450 nm and λ emission = 520 nm (slit 5/5) for pyranine.

Statistical analysis

All statistical analyses were done in GraphPad (La Jolla, CA) Prism v6 for Windows. Bar graphs were generated with GraphPad Prism. Tests and individual *P* values as indicated in figure legends. The data are presented as mean ± SD of independent (biological) replicates unless specified.

Data availability

The data mass spectrometry proteomics ProteomeXchange datasets with identifiers PXD029607 and PXD029646 are publicly available via the PRIDE database (Perez-Riverol *et al*, 2018) under http://www.ebi.ac.uk/pride/archive/projects/PXD029607 and http://www.ebi.ac.uk/pride/archive/projects/PXD029646.

Expanded View for this article is available online.

Acknowledgements

We thank Dr Martha Giacomello for constructive discussion and experimental support and Dr Paolo Bernardi for critical reading of the manuscript. This work was supported by the fellowship DOC-24273 from the Austrian Academy of Science ÖAW to SA, by the Austrian Science Funds FWF research project grant numbers P-31471 and P-29077 to KN and by the Swiss National Science Foundation grant number 310030_189042 to NDe.

Author contributions

Shane Austin: Conceptualization; data curation; formal analysis; validation; investigation; visualization; methodology; writing—original draft; writing—review and editing. **Ronald Mekis:** Data curation; formal analysis; validation; investigation; visualization; methodology; writing—review and editing. **Sami EM Mohammed:** Data curation; formal analysis; validation; investigation; visualization; methodology; writing—original draft; writing—review and editing. **Mariafrancesca Scalise:** Data curation; formal analysis; validation; investigation; visualization; methodology; writing—review and editing. **Wen-An Wang:** Data curation; formal analysis; validation; investigation; visualization; methodology; writing—review and editing. **Michele Galluccio:** Investigation; methodology; writing—review and editing. **Christina Pfeiffer:** Investigation; methodology; writing—review and editing. **Tamara Borovec:** Investigation; methodology. **Katja Parapatics:** Methodology. **Dijana Vitko:** Methodology. **Nora Dinhopl:** Visualization; methodology; writing—review and editing. **Nicolas Demaurex:** Conceptualization; data curation; writing—original draft; writing—review and editing. **Keiryn L Bennett:** Conceptualization; data curation; formal analysis; methodology; writing—review and editing. **Cesare Indiveri:** Conceptualization; data curation; writing—original draft; writing—review and editing. **Karin Nowikovsky:** Conceptualization; resources; data curation; supervision; funding acquisition; investigation; methodology; writing—original draft; writing—review and editing.

In addition to the CRediT author contributions listed above, the contributions in detail are:

KN conceived the project. KN, SA, SEMM, NDe, CI, and KLB wrote the manuscript. SA, RM, SEMM, MS, MG, W-AW, CP, and TB performed and analyzed experiments. KP and DV participated in method development and running of LC–MS instrumentation. NDi performed TEM. KN, NDe, CI, and KLB conceptualized experiments. All authors edited the final manuscript.

Disclosure and competing interests statement

The authors declare that they have no conflict of interest.

References

Austin S, Nowikovsky K (2019) LETM1: essential for mitochondrial biology and cation homeostasis? *Trends Biochem Sci* 44: 648 – 658

Austin S, Nowikovsky K (2021) Mitochondrial osmoregulation in evolution, cation transport and metabolism. *Biochim Biophys Acta Bioenerg* 1862: 148368

Austin S, Tavakoli M, Pfeiffer C, Seifert J, Mattarei A, De Stefani D, Zoratti M, Nowikovsky K (2017) LETM1-mediated K(+) and Na(+) homeostasis regulates mitochondrial Ca(2+) efflux. *Front Physiol* 8: 839

Basso E, Petronilli V, Forte MA, Bernardi P (2008) Phosphate is essential for inhibition of the mitochondrial permeability transition pore by cyclosporin a and by cyclophilin D ablation. *J Biol Chem* 283: 26307 – 26311

Beghi E, Giussani G (2018) Aging and the epidemiology of epilepsy. *Neuroepidemiology* 51: 216 – 223

Bernardi P (1999) Mitochondrial transport of cations: channels, exchangers, and permeability transition. *Physiol Rev* 79: 1127 – 1155

Blomen VA, Majek P, Jae LT, Bigenzahn JW, Nieuwenhuis J, Staring J, Sacco R, van Diemen FR, Olk N, Stukalov A *et al* (2015) Gene essentiality and synthetic lethality in haploid human cells. *Science* 350: 1092 – 1096

Carafoli E, Tiozzo R, Lugli G, Crovetti F, Kratzing C (1974) The release of calcium from heart mitochondria by sodium. *J Mol Cell Cardiol* 6: 361 – 371

Carrara G, Saraiva N, Gubser C, Johnson BF, Smith GL (2012) Six-transmembrane topology for Golgi anti-apoptotic protein (GAAP) and Bax inhibitor 1 (BI-1) provides model for the transmembrane Bax inhibitor-containing motif (TMBIM) family. *J Biol Chem* 287: 15896 – 15905

Carrer A, Tommasin L, Sileikyte J, Ciscato F, Filadi R, Urbani A, Forte M, Rasola A, Szabo I, Carraro M *et al* (2021) Defining the molecular mechanisms of the mitochondrial permeability transition through genetic manipulation of F-ATP synthase. *Nat Commun* 12: 4835

Choi H, Larsen B, Lin ZY, Breitkreutz A, Mellacheruvu D, Fermin D, Qin ZS, Tyers M, Gingras AC, Nesvizhskii AI (2011) SAINT: probabilistic scoring of affinity purification-mass spectrometry data. *Nat Methods* 8: 70 – 73

Cogliati S, Herranz F, Ruiz-Cabello J, Enriquez JA (2021) Digitonin concentration is determinant for mitochondrial supercomplexes analysis by BlueNative page. *Biochim Biophys Acta Bioenerg* 1862: 148332

Cosco J, Scalise M, Colas C, Galluccio M, Martini R, Rovella F, Mazza T, Ecker GF, Indiveri C (2020) ATP modulates SLC7A5 (LAT1) synergistically with cholesterol. *Sci Rep* 10: 16738

De Marchi U, Castelbou C, Demaurex N (2011) Uncoupling protein 3 (UCP3) modulates the activity of Sarco/endoplasmic reticulum Ca^{2+}-ATPase (SERCA) by decreasing mitochondrial ATP production. *J Biol Chem* 286: 32533 – 32541

De Stefani D, Rizzuto R, Pozzan T (2016) Enjoy the trip: calcium in mitochondria Back and forth. *Annu Rev Biochem* 85: 161 – 192

Del Dotto V, Mishra P, Vidoni S, Fogazza M, Maresca A, Caporali L, McCaffery JM, Cappelletti M, Baruffini E, Lenaers G *et al* (2017) OPA1 isoforms in the hierarchical Organization of Mitochondrial Functions. *Cell Rep* 19: 2557 – 2571

Doonan PJ, Chandramoorthy HC, Hoffman NE, Zhang X, Cardenas C, Shanmughapriya S, Rajan S, Vallem S, Chen X, Foskett JK *et al* (2014) LETM1-dependent mitochondrial Ca^{2+} flux modulates cellular bioenergetics and proliferation. *FASEB J* 28: 4936 – 4949

Frezza C, Cipolat S, Scorrano L (2007) Organelle isolation: functional mitochondria from mouse liver, muscle and cultured fibroblasts. *Nat Protoc* 2: 287 – 295

Galluccio M, Pingitore P, Scalise M, Indiveri C (2013) Cloning, large scale over-expression in *E. coli* and purification of the components of the human LAT 1 (SLC7A5) amino acid transporter. *Protein J* 32: 442 – 448

Giambruno R, Grebien F, Stukalov A, Knoll C, Planyavsky M, Rudashevskaya EL, Colinge J, Superti-Furga G, Bennett KL (2013) Affinity purification strategies for proteomic analysis of transcription factor complexes. *J Proteome Res* 12: 4018 – 4027

Giorgi C, Marchi S, Pinton P (2018) The machineries, regulation and cellular functions of mitochondrial calcium. *Nat Rev Mol Cell Biol* 19: 713 – 730

Glatter T, Wepf A, Aebersold R, Gstaiger M (2009) An integrated workflow for charting the human interaction proteome: Insights into the PP2A system. *Mol Syst Biol* 5: 237

Grandits AM, Nguyen CH, Schlerka A, Hackl H, Sill H, Etzler J, Heyes E, Stoiber D, Grebien F, Heller G *et al* (2021) Downregulation of MTSS1 in acute myeloid leukemia is associated with a poor prognosis, chemotherapy resistance, and disease aggressiveness. *Leukemia* 35: 2827 – 2839

Guo G, Xu M, Chang Y, Luyten T, Seitaj B, Liu W, Zhu P, Bultynck G, Shi L, Quick M *et al* (2019b) Ion and pH sensitivity of a TMBIM Ca(2+) channel. *Structure* 27: e1013

Guo L, Carraro M, Carrer A, Minervini G, Urbani A, Masgras I, Tosatto SCE, Szabo I, Bernardi P, Lippe G (2019a) Arg-8 of yeast subunit e contributes to the stability of F-ATP synthase dimers and to the generation of the full-conductance mitochondrial megachannel. *J Biol Chem* 294: 10987 – 10997

Hasegawa A, van der Bliek AM (2007) Inverse correlation between expression of the wolfs Hirschhorn candidate gene Letm1 and mitochondrial volume in *C. elegans* and in mammalian cells. *Hum Mol Genet* 16: 2061 – 2071

Hashimi H, McDonald L, Stribrna E, Lukes J (2013) Trypanosome Letm1 protein is essential for mitochondrial potassium homeostasis. *J Biol Chem* 288: 26914 – 26925

Hung YP, Albeck JG, Tantama M, Yellen G (2011) Imaging cytosolic NADH-NAD(+) redox state with a genetically encoded fluorescent biosensor. *Cell Metab* 14: 545 – 554

Jiang D, Zhao L, Clapham DE (2009) Genome-wide RNAi screen identifies Letm1 as a mitochondrial Ca^{2+}/H^{+} antiporter. *Science* 326: 144 – 147

Jiang D, Zhao L, Clish CB, Clapham DE (2013) Letm1, the mitochondrial Ca^{2+}/H^{+} antiporter, is essential for normal glucose metabolism and alters brain function in Wolf-Hirschhorn syndrome. *Proc Natl Acad Sci USA* 110: E2249 – E2254

Jinek M, Chylinski K, Fonfara I, Hauer M, Doudna JA, Charpentier E (2012) A programmable dual-RNA-guided DNA endonuclease in adaptive bacterial immunity. *Science* 337: 816 – 821

Jumper J, Evans R, Pritzel A, Green T, Figurnov M, Ronneberger O, Tunyasuvunakool K, Bates R, Zidek A, Potapenko A *et al* (2021) Highly accurate protein structure prediction with AlphaFold. *Nature* 596: 583 – 589

Kim HK, Lee GH, Bhattarai KR, Lee MS, Back SH, Kim HR, Chae HJ (2021) TMBIM6 (transmembrane BAX inhibitor motif containing 6) enhances autophagy through regulation of lysosomal calcium. *Autophagy* 17: 761 – 778

Korge P, Weiss JN (1999) Thapsigargin directly induces the mitochondrial permeability transition. *Eur J Biochem* 265: 273 – 280

Li S, Wang L, Berman M, Kong YY, Dorf ME (2011) Mapping a dynamic innate immunity protein interaction network regulating type I interferon production. *Immunity* 35: 426 – 440

Lisak DA, Schacht T, Enders V, Habicht J, Kiviluoto S, Schneider J, Henke N, Bultynck G, Methner A (2015) The transmembrane Bax inhibitor motif (TMBIM) containing protein family: Tissue expression, intracellular localization and effects on the ER CA(2)(+)-filling state. *Biochim Biophys Acta* 1853: 2104 – 2114

Liu Q (2017) TMBIM-mediated Ca(2+) homeostasis and cell death. *Biochim Biophys Acta Mol Cell Res* 1864: 850 – 857

Luongo TS, Lambert JP, Gross P, Nwokedi M, Lombardi AA, Shanmughapriya S, Carpenter AC, Kolmetzky D, Gao E, van Berlo JH *et al* (2017) The

© 2022 The Authors

mitochondrial Na(+)/Ca(2+) exchanger is essential for Ca(2+) homeostasis and viability. *Nature* 545: 93–97

McQuibban AG, Joza N, Megighian A, Scorzeto M, Zanini D, Reipert S, Richter C, Schweyen RJ, Nowikovsky K (2010) A Drosophila mutant of LETM1, a candidate gene for seizures in Wolf-Hirschhorn syndrome. *Hum Mol Genet* 19: 987–1000

Mellacheruvu D, Wright Z, Couzens AL, Lambert JP, St-Denis NA, Li T, Miteva YV, Hauri S, Sardiu ME, Low TY *et al* (2013a) CRAPome 2.0 database (https://reprint-apms.org/?q=chooseworkflow). [DATASET]

Mellacheruvu D, Wright Z, Couzens AL, Lambert JP, St-Denis NA, Li T, Miteva YV, Hauri S, Sardiu ME, Low TY *et al* (2013b) The CRAPome: a contaminant repository for affinity purification-mass spectrometry data. *Nat Methods* 10: 730–736

Mitchell P (1966) Chemiosmotic coupling in oxidative and photosynthetic phosphorylation. *Biol Rev Camb Philos Soc* 41: 445–502

Moreno-Justicia R, Gonzalez-Franquesa A, Stocks B, Deshmukh AS (2022) Protocol to characterize mitochondrial supercomplexes from mouse tissues by combining BN-PAGE and MS-based proteomics. *STAR Protoc* 3: 101135

Nowikovsky K, Bernardi P (2014) LETM1 in mitochondrial cation transport. *Front Physiol* 5: 83

Nowikovsky K, Froschauer EM, Zsurka G, Samaj J, Reipert S, Kolisek M, Wiesenberger G, Schweyen RJ (2004) The LETM1/YOL027 gene family encodes a factor of the mitochondrial K+ homeostasis with a potential role in the Wolf-Hirschhorn syndrome. *J Biol Chem* 279: 30307–30315

Nowikovsky K, Pozzan T, Rizzuto R, Scorrano L, Bernardi P (2012) Perspectives on: SGP symposium on mitochondrial physiology and medicine: the pathophysiology of LETM1. *J Gen Physiol* 139: 445–454

Nowikovsky K, Reipert S, Devenish RJ, Schweyen RJ (2007) Mdm38 protein depletion causes loss of mitochondrial K^+/H^+ exchange activity, osmotic swelling and mitophagy. *Cell Death Differ* 14: 1647–1656

Oka T, Sayano T, Tamai S, Yokota S, Kato H, Fujii G, Mihara K (2008) Identification of a novel protein MICS1 that is involved in maintenance of mitochondrial morphology and apoptotic release of cytochrome c. *Mol Biol Cell* 19: 2597–2608

Olichon A, Baricault L, Gas N, Guillou E, Valette A, Belenguer P, Lenaers G (2003) Loss of OPA1 perturbates the mitochondrial inner membrane structure and integrity, leading to cytochrome c release and apoptosis. *J Biol Chem* 278: 7743–7746

Olsen JV, de Godoy LM, Li G, Macek B, Mortensen P, Pesch R, Makarov A, Lange O, Horning S, Mann M (2005) Parts per million mass accuracy on an orbitrap mass spectrometer via lock mass injection into a C-trap. *Mol Cell Proteomics* 4: 2010–2021

Pallafacchina G, Zanin S, Rizzuto R (2018) Recent advances in the molecular mechanism of mitochondrial calcium uptake. *F1000Res* 7: 1858

Patron M, Tarasenko D, Nolte H, Kroczek L, Ghosh M, Ohba Y, Lasarzewski Y, Ahmadi ZA, Cabrera-Orefice A, Eyiama A *et al* (2022) Regulation of mitochondrial proteostasis by the proton gradient. *EMBO J* 41: e110476

Perez-Riverol Y, Csordas A, Bai J, Bernal-Llinares M, Hewapathirana S, Kundu DJ, Inuganti A, Griss J, Mayer G, Eisenacher M *et al* (2018) The PRIDE database and related tools and resources in 2019: improving support for quantification data. *Nucleic Acids Res* 47: D442–D450

Pichlmair A, Kandasamy K, Alvisi G, Mulhern O, Sacco R, Habjan M, Binder M, Stefanovic A, Eberle CA, Goncalves A *et al* (2012) Viral immune modulators perturb the human molecular network by common and unique strategies. *Nature* 487: 486–490

Pinto PH, Kroupova A, Schleiffer A, Mechtler K, Jinek M, Weitzer S, Martinez J (2020) ANGEL2 is a member of the CCR4 family of deadenylases with 2′,3′-cyclic phosphatase activity. *Science* 369: 524–530

Poburko D, Santo-Domingo J, Demaurex N (2011) Dynamic regulation of the mitochondrial proton gradient during cytosolic calcium elevations. *J Biol Chem* 286: 11672–11684

Quintana-Cabrera R, Quirin C, Glytsou C, Corrado M, Urbani A, Pellattiero A, Calvo E, Vazquez J, Enriquez JA, Gerle C *et al* (2018) The cristae modulator optic atrophy 1 requires mitochondrial ATP synthase oligomers to safeguard mitochondrial function. *Nat Commun* 9: 3399

Rappsilber J, Mann M, Ishihama Y (2007) Protocol for micro-purification, enrichment, pre-fractionation and storage of peptides for proteomics using StageTips. *Nat Protoc* 2: 1896–1906

Rojas-Rivera D, Hetz C (2015) TMBIM protein family: ancestral regulators of cell death. *Oncogene* 34: 269–280

Rudashevskaya EL, Sacco R, Kratochwill K, Huber ML, Gstaiger M, Superti-Furga G, Bennett KL (2013) A method to resolve the composition of heterogeneous affinity-purified protein complexes assembled around a common protein by chemical cross-linking, gel electrophoresis and mass spectrometry. *Nat Protoc* 8: 75–97

Sambri I, Massa F, Gullo F, Meneghini S, Cassina L, Carraro M, Dina G, Quattrini A, Patanella L, Carissimo A *et al* (2020) Impaired flickering of the permeability transition pore causes SPG7 spastic paraplegia. *EBioMedicine* 61: 103050

Sancak Y, Markhard AL, Kitami T, Kovacs-Bogdan E, Kamer KJ, Udeshi ND, Carr SA, Chaudhuri D, Clapham DE, Li AA *et al* (2013) EMRE is an essential component of the mitochondrial calcium uniporter complex. *Science* 342: 1379–1382

Scalise M, Mazza T, Pappacoda G, Pochini L, Cosco J, Rovella F, Indiveri C (2020) The human SLC1A5 neutral amino acid transporter catalyzes a pH-dependent glutamate/glutamine antiport, as well. *Front Cell Dev Biol* 8: 603

Scalise M, Pochini L, Console L, Pappacoda G, Pingitore P, Hedfalk K, Indiveri C (2018) Cys site-directed mutagenesis of the human SLC1A5 (ASCT2) transporter: structure/function relationships and crucial role of Cys467 for redox sensing and glutamine transport. *Int J Mol Sci* 19: 648

Seitaj B, Maull F, Zhang L, Wullner V, Wolf C, Schippers P, La Rovere R, Distler U, Tenzer S, Parys JB *et al* (2020) Transmembrane BAX Inhibitor-1 motif containing protein 5 (TMBIM5) sustains mitochondrial structure, shape, and function by impacting the mitochondrial protein synthesis machinery. *Cell* 9: 2147

Shao J, Fu Z, Ji Y, Guan X, Guo S, Ding Z, Yang X, Cong Y, Shen Y (2016) Leucine zipper-EF-hand containing transmembrane protein 1 (LETM1) forms a Ca(2+)/H(+) antiporter. *Sci Rep* 6: 34174

Skucha A, Ebner J, Schmollerl J, Roth M, Eder T, Cesar-Razquin A, Stukalov A, Vittori S, Muhar M, Lu B *et al* (2018) MLL-fusion-driven leukemia requires SETD2 to safeguard genomic integrity. *Nat Commun* 9: 1983

Urbani A, Prosdocimi E, Carrer A, Checchetto V, Szabo I (2020) Mitochondrial ion channels of the inner membrane and their regulation in cell death signaling. *Front Cell Dev Biol* 8: 620081

Wilfinger N, Austin S, Scheiber-Mojdekhar B, Berger W, Reipert S, Praschberger M, Paur J, Trondl R, Keppler BK, Zielinski CC *et al* (2016) Novel p53-dependent anticancer strategy by targeting iron signaling and BNIP3L-induced mitophagy. *Oncotarget* 7: 1242–1261

Zhang L, Dietsche F, Seitaj B, Rojas-Charry L, Latchman N, Tomar D, Wust RC, Nickel A, Frauenknecht KB, Schoser B *et al* (2022) TMBIM5 loss of function alters mitochondrial matrix ion homeostasis and causes a skeletal myopathy. *Life Sci Alliance* 5: e202201478

License: This is an open access article under the terms of the Creative Commons Attribution License, which permits use, distribution and reproduction in any medium, provided the original work is properly cited.

Expanded View Figures

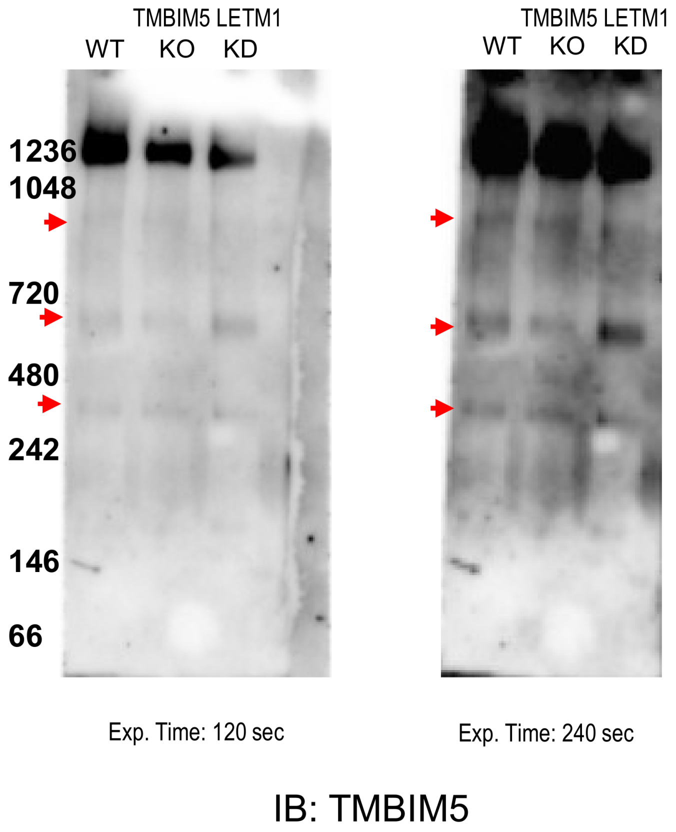

Figure EV1. TMBIM5 and LETM1 are present in protein complexes of the same molecular weight.

Immunoblotting analysis of blue native PAGE of isolated mitochondria from HeLa WT, TMBIM5KO, or LETM1KD cells, using the indicated antibody, arrows indicate TMBIM5 complexes.

 © 2022 The Authors

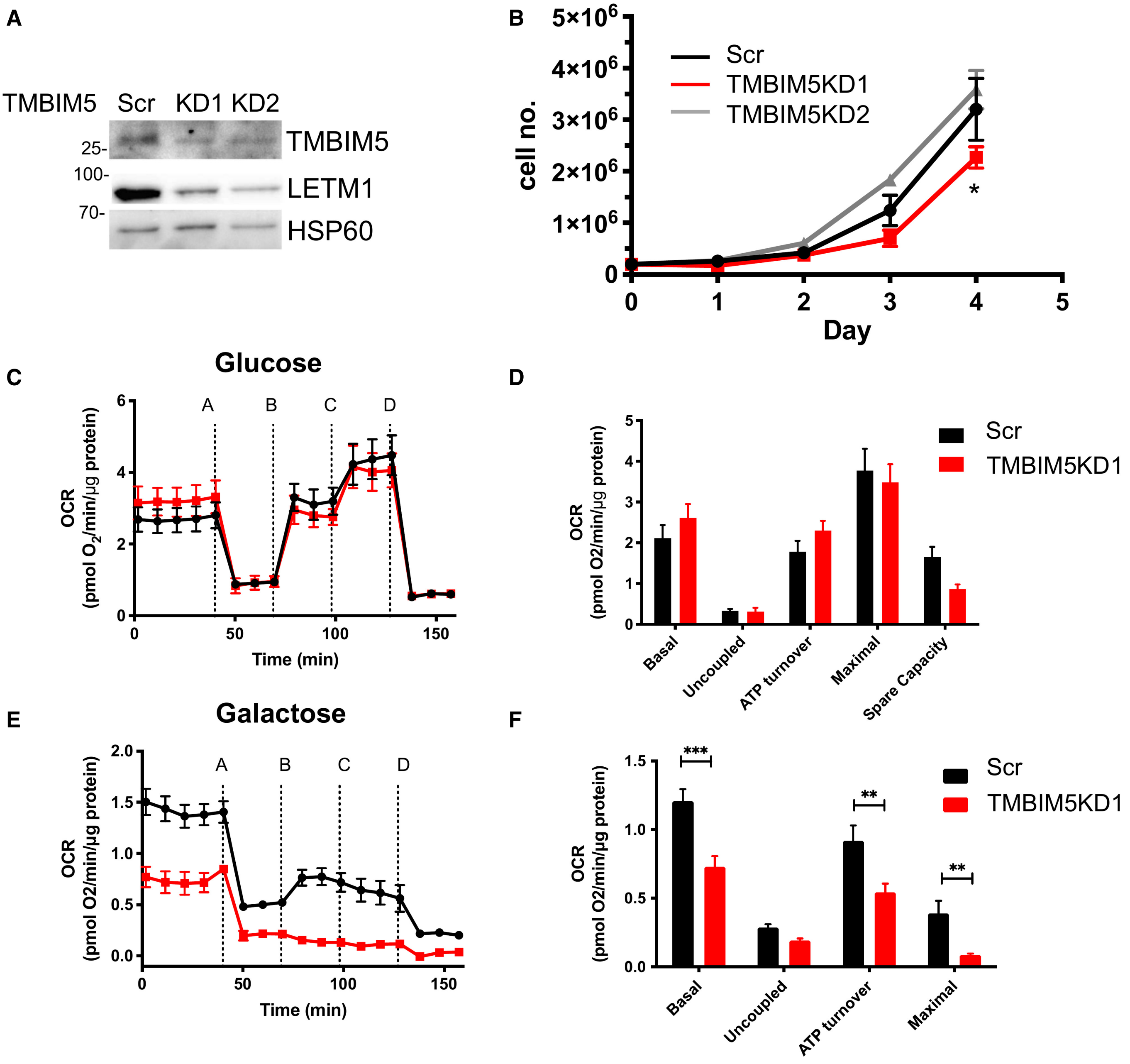

Figure EV2. TMBIM5KD decreases LETM1 and mitochondrial bioenergetics.

A Western blot analysis of LETM1 and TMBIM5 in HEK293 TMBIM5WT cells with a scramble shRNA and two different TMBIM5 knockdowns, HSP60 served as a loading control.

B Proliferation curve of TMBIM5WT scramble controls (scr) compared with TMBIM5KD cells (KD) over 4 days. Data are means ± SEM (scr, KD2 $n = 3$, KD1 $n = 5$) (biological replicates), at 96 h statistical analysis using an unpaired student's *t*-test (*$P < 0.05$).

C–F Cellular bioenergetics of TMBIM5KD cells in various nutrient conditions. Oxygen consumption rate of WT cells with a scrambled control (WT) and TMBIM5KD cells (KD) grown in (C) 25 mM glucose, (E) 10 mM galactose for 24 h before measurement. Data are representative of at least three independent experiments (biological replicates). Shown are the mean data of triplicate measurements ± SEM. Inhibitors as indicated: (A) oligomycin (0.5 μM), (B, C) FCCP (0.2 μM each), (D) antimycin A/rotenone (0.5 μM). (D, F) Bar charts of XF experiment traces (C, E), data are means of multiple time points after experiment start or drug addition of at least three independent experiments ± SEM (biological replicates). Statistical analysis using an unpaired student's *t*-test (**$P < 0.01$, ***$P < 0.001$).

Source data are available online for this figure.

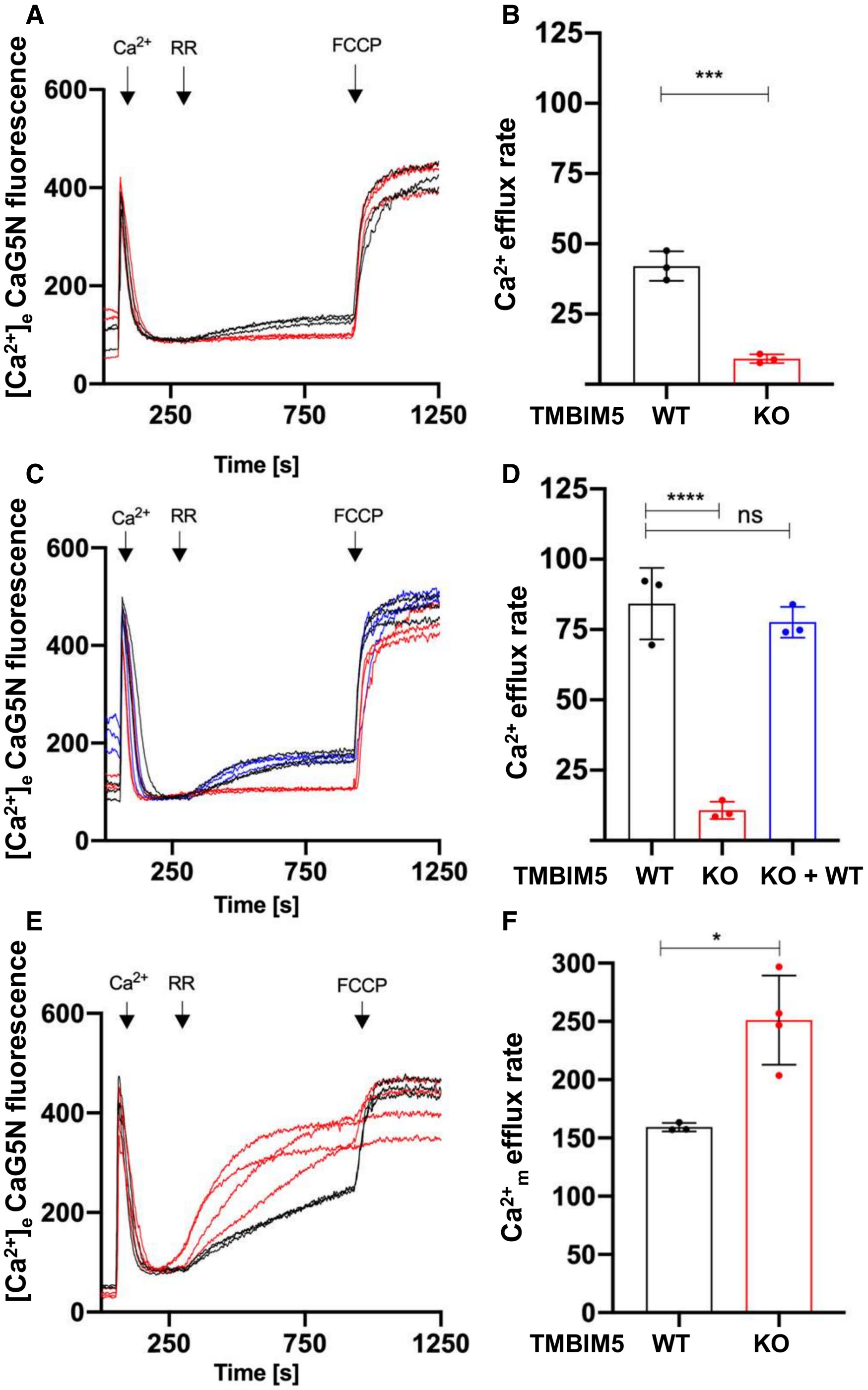

Figure EV3. TMBIM5 mitochondrial Na^{+}-dependent Ca^{2+} release in HeLa cells.

A–D Ca^{2+} uptake release assays were conducted in permeabilized HeLa TMBIM5WT or TMBIM5KO cells or TMBIM5KO cells expressing pcDNA3.1+TMBIM5 in presence of CGP37157 (2 μM) as described in Fig 4A–D applying a 10 μM (A) or 20 μM (C) Ca^{2+} pulse. In (C) Ca^{2+} uptake release was recorded in permeabilized HeLa TMBIM5WT or TMBIM5KO cells or TMBIM5KO re-expressing TMBIM5 cells.

E, F Same experimental setting as in (C) but in presence of Tg (1 μM). Quantification of ≥ 3 independent experiments (biological replicates), data are the mean ± SD with an unpaired student's test (B, F) and one-way ANOVA with Bonferroni correction (D), (*$P < 0.05$, ***$P < 0.001$, ****$P < 0.0001$, ns, not significant).

Source data are available online for this figure.

 © 2022 The Authors

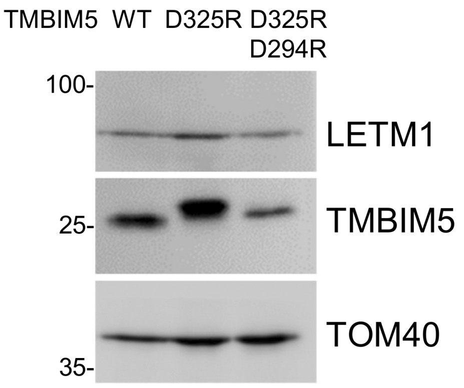

Figure EV4. TMBIM5 protein levels are not decreased by TMBIM5^{D325R}.

Immunoblots of Isolated mitochondria from HEK293 TMBIM5WT and TMBIM5KO expressing TMBIM5^{D325R} or double mutant TMBIM5$^{D325R/D294R}$ using the indicated antibodies, TOM40 served as mitochondrial loading control.

Source data are available online for this figure.

Figure EV5. TMBIM5KO induces PTP opening under Ca^{2+} overload.

A–H Ca^{2+} uptake/release dynamics in presence of CGP37157 (2 μM) and Tg (1 μM) were monitored as in Fig 5. Ca^{2+} (10 μM), RR (0.2 μM), and FCCP (2 μM) were added when indicated. Membrane potential was recorded as the change in fluorescence intensities of TMRM (330 nM) (C, D) corresponding to the measurement of Ca^{2+} fluxes in (A, B). CsA was added 2 min before measurements in (E, F dotted lines). Quantification of Ca^{2+} release rates from three independent experiments (biological replicates) are shown as means ± SD (t: 300–920 s) and statistical analysis: One-Way ANOVA with Bonferroni correction (*$P < 0.05$, **$P < 0.01$, ***$P < 0.001$, ****$P < 0.0001$). Quantification of TMRM performed with an unpaired two-sided t-test (Welsh correction), *$P < 0.05$. (E, F) Calcium retention capacity (CRC) assays showing that the absence of TMBIM5 supersensitizes mitochondria to Ca^{2+}-induced PTP opening by Tg. See also Appendix Fig S6A and B for CRCs in absence of Tg. Permeabilized HEK293 TMBIM5WT (E) and TMBIM5KO1 (F) cells exposed or not to CsA were subjected to sequential Ca^{2+} bolus of 5 μM Ca^{2+}, and fluorescence intensity was recorded.

Source data are available online for this figure.

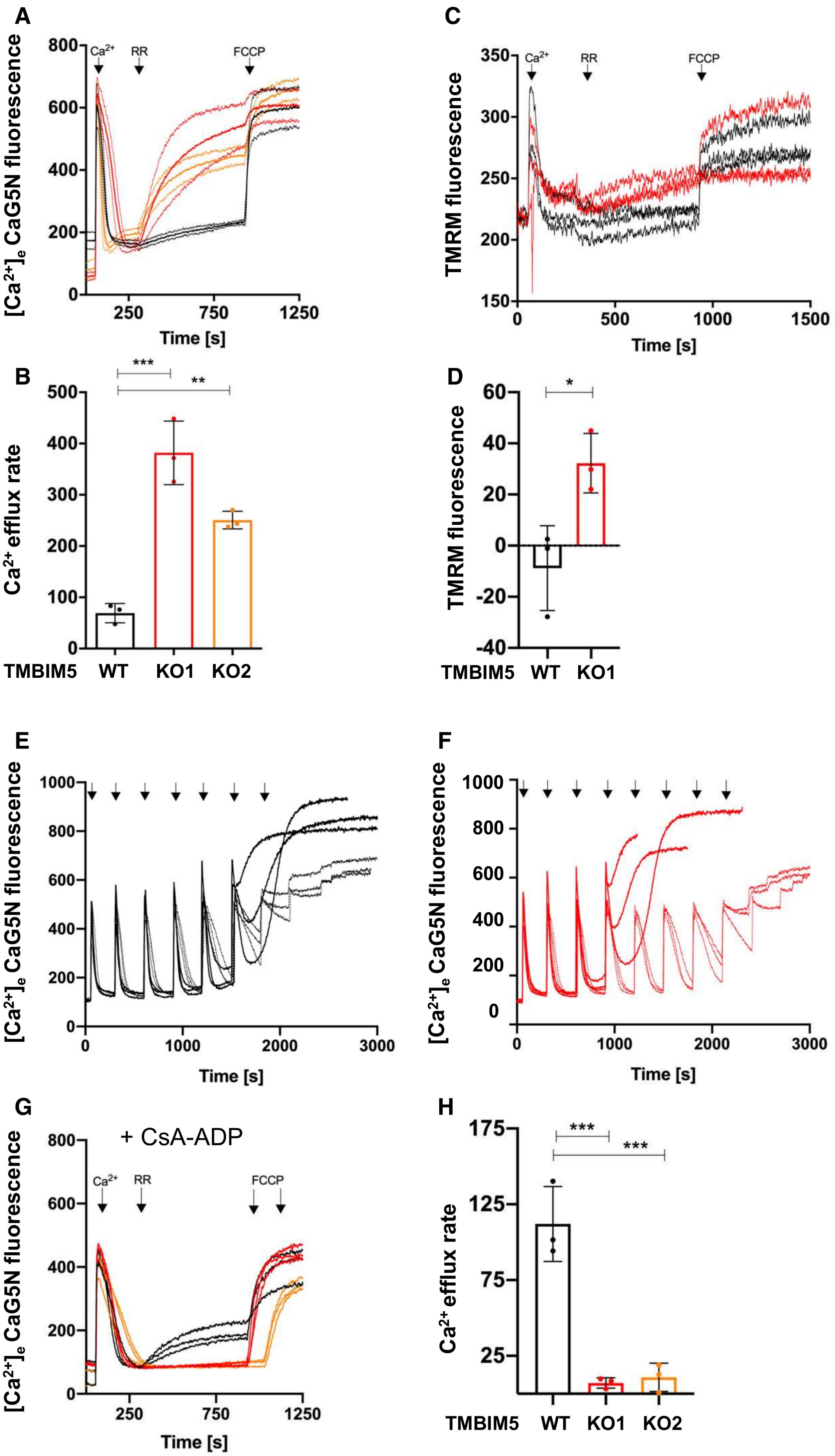

Figure EV5.

© 2022 The Authors
