# Supplementary material for: TMBIM5 is the Ca2+/H+ antiporter of mammalian mitochondria
Source: EMBO Rep. 2022 Nov 2;23(12):e54978. doi: 10.15252/embr.202254978 (PMC9724676; doi:10.15252/embr.202254978)
Supplement: Supplementary file 13 [file EMBR-23-e54978-s009.pdf]

EMBO reports

TMBIM5 is the $Ca2^+/H^+$ antiporter of mammalian mitochondria

Shane Austin, Ronald Mekis, Sami Mohammed, Mariafrancesca Scalise, Wen-An Wang, Michele Galluccio, Christina Pfeiffer, Tamara Borovec, Katja Parapatics, Dijana Vitko, Nora Dinhopl, Nicolas Demaurex, Keiryn Bennett, Cesare Indiveri, and Karin Nowikovsky
DOI: 10.15252/embr.202254978

Corresponding author(s): Karin Nowikovsky (karin.nowikovsky@vetmeduni.ac.at)

Review Timeline:

Submission Date:	4th Mar 22
Editorial Decision:	4th Apr 22
Revision Received:	6th Jul 22
Editorial Decision:	18th Aug 22
Revision Received:	7th Sep 22
Accepted:	7th Oct 22

Editor: Martina Rembold/Deniz Senyilmaz-Tiebe

Transaction Report:

(Note: With the exception of the correction of typographical or spelling errors that could be a source of ambiguity, letters and reports are not edited. Depending on transfer agreements, referee reports obtained elsewhere may or may not be included in this compilation. Referee reports are anonymous unless the Referee chooses to sign their reports.)

Dear Dr. Nowikovsky,

Thank you for the submission of your research manuscript to our journal, which was now seen by three referees, whose reports are copied below.

We concur with the referees that the proposed role of MICS1/TMBIM5 as mitochondrial Ca2+/H+ antiporter is in principle very interesting. However, referees also raise some concerns that need to be addressed to consider publication here.

I find the reports informed and constructive, and believe that addressing the concerns raised will significantly strengthen the manuscript. As the reports are below, and I think all points need to be addressed, I will not detail them here.

Given these positive recommendations, we would like to invite you to revise your manuscript with the understanding that the referee concerns (as in their reports) must be fully addressed and their suggestions taken on board. Please address all referee concerns in a complete point-by-point response. Acceptance of the manuscript will depend on a positive outcome of a second round of review. It is EMBO reports policy to allow a single round of revision only and acceptance or rejection of the manuscript will therefore depend on the completeness of your responses included in the next, final version of the manuscript.

We generally allow three months as standard revision time. As a matter of policy, competing manuscripts published during this period will not negatively impact on our assessment of the conceptual advance presented by your study. However, we request that you contact the editor as soon as possible upon publication of any related work, to discuss how to proceed. Should you foresee a problem in meeting this three-month deadline, please let us know in advance and we may be able to grant an extension.

*** Temporary update to EMBO Press scooping protection policy:
We are aware that many laboratories cannot function at full efficiency during the current COVID-19/SARS-CoV-2 pandemic and have therefore extended our 'scooping protection policy' to cover the period required for a full revision to address the experimental issues highlighted in the editorial decision letter. Please contact the scientific editor handling your manuscript to discuss a revision plan should you need additional time, and also if you see a paper with related content published elsewhere.***

IMPORTANT NOTE: we perform an initial quality control of all revised manuscripts before re-review. Your manuscript will FAIL this control and the handling will be DELAYED if the following APPLIES:
1. A data availability section providing access to data deposited in public databases is missing (where applicable).
2. Your manuscript contains statistics and error bars based on n=2. Please use scatter plots in these cases.

You can submit the revision either as a Scientific Report or as a Research Article. For Scientific Reports, the revised manuscript can contain up to 5 main figures and 5 Expanded View figures. If the revision leads to a manuscript with more than 5 main figures it will be published as a Research Article. In this case the Results and Discussion section should be separate. If a Scientific Report is submitted, these sections have to be combined. This will help to shorten the manuscript text by eliminating some redundancy that is inevitable when discussing the same experiments twice. In either case, all materials and methods should be included in the main manuscript file

Supplementary/additional data: The Expanded View format, which will be displayed in the main HTML of the paper in a collapsible format, has replaced the Supplementary information. You can submit up to 5 images as Expanded View. Please follow the nomenclature Figure EV1, Figure EV2 etc. The figure legend for these should be included in the main manuscript document file in a section called Expanded View Figure Legends after the main Figure Legends section. Additional Supplementary material should be supplied as a single pdf labeled Appendix. The Appendix includes a table of content on the first page with page numbers, all figures and their legends. Please follow the nomenclature Appendix Figure Sx throughout the text and also label the figures according to this nomenclature. For more details please refer to our guide to authors.

Please note that for all articles published beginning 1 July 2020, the EMBO Reports reference style will change to the Harvard style for all article types. Details and examples are provided at
https://www.embopress.org/page/journal/14693178/authorguide#referencesformat

When submitting your revised manuscript, please carefully review the instructions that follow below. Failure to include requested items will delay the evaluation of your revision.

1) a .docx formatted version of the manuscript text (including legends for main figures, EV figures and tables). Please make sure that the changes are highlighted to be clearly visible.

2) individual production quality figure files as .eps, .tif, .jpg (one file per figure).

3) a .docx formatted letter INCLUDING the reviewers' reports and your detailed point-by-point responses to their comments. As

part of the EMBO Press transparent editorial process, the point-by-point response is part of the Review Process File (RPF), which will be published alongside your paper. For more details on our Transparent Editorial Process, please visit our website: https://www.embopress.org/page/journal/14693178/authorguide#transparentprocess
You are able to opt out of this by letting the editorial office know (emboreports@embo.org). If you do opt out, the Review Process File link will point to the following statement: "No Review Process File is available with this article, as the authors have chosen not to make the review process public in this case."

4) a complete author checklist, which you can download from our author guidelines (<http://embor.embopress.org/authorguide>). Please insert information in the checklist that is also reflected in the manuscript. The completed author checklist will also be part of the RPF.

5) Please note that all corresponding authors are required to supply an ORCID ID for their name upon submission of a revised manuscript (<https://orcid.org/>). Please find instructions on how to link your ORCID ID to your account in our manuscript tracking system in our Author guidelines (<http://embor.embopress.org/authorguide>).

6) We replaced Supplementary Information with Expanded View (EV) Figures and Tables that are collapsible/expandable online. A maximum of 5 EV Figures can be typeset. EV Figures should be cited as 'Figure EV1, Figure EV2" etc... in the text and their respective legends should be included in the main text after the legends of regular figures.

- For the figures that you do NOT wish to display as Expanded View figures, they should be bundled together with their legends in a single PDF file called *Appendix*, which should start with a short Table of Content. Appendix figures should be referred to in the main text as: "Appendix Figure S1, Appendix Figure S2" etc. See detailed instructions regarding expanded view here: <http://embor.embopress.org/authorguide#expandedview>.

- Additional Tables/Datasets should be labeled and referred to as Table EV1, Dataset EV1, etc. Legends have to be provided in a separate tab in case of .xls files. Alternatively, the legend can be supplied as a separate text file (README) and zipped together with the Table/Dataset file.

7) We would also encourage you to include the source data for figure panels that show essential data.

Numerical data should be provided as individual .xls or .csv files (including a tab describing the data). For blots or microscopy, uncropped images should be submitted (using a zip archive if multiple images need to be supplied for one panel). Additional information on source data and instruction on how to label the files are available <http://embor.embopress.org/authorguide#sourcedata>.

8) Our journal encourages inclusion of *data citations in the reference list* to directly cite datasets that were re-used and obtained from public databases. Data citations in the article text are distinct from normal bibliographical citations and should directly link to the database records from which the data can be accessed. In the main text, data citations are formatted as follows: "Data ref: Smith et al, 2001" or "Data ref: NCBI Sequence Read Archive PRJNA342805, 2017". In the Reference list, data citations must be labeled with "[DATASET]". A data reference must provide the database name, accession number/identifiers and a resolvable link to the landing page from which the data can be accessed at the end of the reference. Further instructions are available at <http://embor.embopress.org/authorguide#datacitation>.

9) Please make sure to include a Data Availability Section before submitting your revision - if it is not applicable, make a statement that no data were deposited in a public database. Primary datasets (and computer code, where appropriate) produced in this study need to be deposited in an appropriate public database (see <http://embor.embopress.org/authorguide#dataavailability>).

Please remember to provide a reviewer password if the datasets are not yet public.

The accession numbers and database should be listed in a formal "Data Availability " section (placed after Materials & Method) that follows the model below. Please note that the Data Availability Section is restricted to new primary data that are part of this study.

Data availability

The datasets (and computer code) produced in this study are available in the following databases:

- RNA-Seq data: Gene Expression Omnibus GSE46843 (https://www.ncbi.nlm.nih.gov/geo/query/acc.cgi?acc=GSE46843)
- [data type]: [name of the resource] [accession number/identifier/doi] ([URL or identifiers.org/DATABASE:ACCESSION])

*** Note - All links should resolve to a page where the data can be accessed. ***

10) Regarding data quantification, please ensure to specify the name of the statistical test used to generate error bars and P values, the number (n) of independent experiments underlying each data point (not replicate measures of one sample), and the test used to calculate p-values in each figure legend. Discussion of statistical methodology can be reported in the materials and methods section, but figure legends should contain a basic description of n, P and the test applied.
Please note that error bars and statistical comparisons may only be applied to data obtained from at least three independent biological replicates.
Please also include scale bars in all microscopy images.

We would also welcome the submission of cover suggestions, or motifs to be used by our Graphics Illustrator in designing a cover.

11) The journal requires a statement specifying whether or not authors have competing interests (defined as all potential or actual interests that could be perceived to influence the presentation or interpretation of an article). In case of competing interests, this must be specified in your disclosure statement. Further information: https://www.embopress.org/competing-interests

I look forward to seeing a revised version of your manuscript when it is ready. Please let me know if you have questions or comments regarding the revision.

Kind regards,

Deniz Senyilmaz Tiebe

Deniz Senyilmaz Tiebe, PhD
Editor
EMBO Reports

Referee #1:

In mammalian cells, mitochondria play a central role in calcium homeostasis. Despite the important role of mitochondria in this context, the components that mediate calcium transport across mitochondrial membranes are only poorly understood. Even the main calcium transporter, the MCU, was only discovered rather recently.
In this study, the authors characterize the inner membrane protein MICS1/TMBIM5 and identify it as a so far uncharacterized calcium/proton antiporter that is associated with the well-known inner membrane protein LETM1. In particular, the characterization of the transport activity of TMBIM5 is very convincing and the authors provide evidence that TMBIM5 mediates natrium-independent calcium efflux from mitochondria under physiological conditions. Given the novelty of TMBIM5 and the comprehensive analysis of the transport activity of TMBIM5 this study will be of interest for many readers in the future, even though several biochemical questions will have to be addressed in the future in follow-up studies. Most experiments are well controlled and the manuscript is well written and clear. The in vitro reconstitution shown in the last figure is particularly interesting, however, seems a bit immature. This aspect needs to be further improved.

Specific points
1. Fig. 1D. The pulldown of LETM1 efficiently co-isolated MICS1/TMBIM5, resulting in comparable signals. However, the pulldown of MICS1/TMBIM5 does barely isolate any LETM1. This is unexpected and should be repeated. Thereby, splicing of the gels should be avoided.
2. Fig. S1D. The authors speculate about a LETM1-MICS complex of 400 kDa that is detectable with antibodies in BN-PAGE experiments. Since they successfully established MICS KO cells, it should be straightforward to test whether the LETM1 complex is shifted in these mutant cell lines. If the complex indeed consists of both proteins, a change in size or abundance would be expected.
3. The proteomics experiments by which MICS1/TMBIM5 was identified are not clearly described and the data are not included in the supplements. The data set is basically limited to the supplemental figure S1c. The authors should improve this and provide the entire ms data set as excel file.
4. There are several studies on MICS1/TMBIM5 that were recently published or are uploaded to BioRXiv. The authors here should also refer to their protein as TMBIM5 (rather than MICS) to avoid confusion.
5. The authors propose that in vivo, MICS1/TMBIM5 and LETM1 form a complex and cooperate. However, their reconstitution experiment shown in Fig. 8 only shows MICS1/TMBIM5 with LETM1. The authors should make use of this very interesting in vitro approach and co-reconstitute LETM1 into the same vesicles. Does LETM1 alter the calcium-transport properties of MICS1/TMBIM5?
6. The data of the reconstitution experiment are very noisy and look like single-run experiments. The authors should put more

emphasis on this interesting reconstitution system, add for example nigericin and elucidate the pH-dependence of MICS1/TMBIM5-mediated calcium transport more comprehensively. This would make this study certainly more attractive.

Minor points
6. The presentation of the y axis in Fig. 5J is odd. The authors should avoid the rescaling here.
7. Size standards should be added to Fig. 8B.

Referee #2:

The paper by Austin and colleagues claims a very strong message in the Ca2+ field, reporting the molecular identification of the H+/ Ca2+ antiporter. The same group previously reported that LETM1, identified as the mitochondrial CHE, mediates K+/H+ antiporter. Here, they defined the interactome of LETM1 and identified MICS1 as the mitochondrial CHE. Notably, a pre-print article by Langer's group (https://www.biorxiv.org/content/10.1101/2021.12.12.470907v1.full.pdf) almost confirmed the results presented here. However, other findings are required to support MICS1 as the CHE.

Mitochondrial Ca2+ measurements should be performed in intact cells, using specific Ca2+ indicators. Moreover, cytosolic Ca2+ transients following mitochondrial Ca2+ efflux should differ between WT e KO cells.
Key experiments should be performed in other cell types.
Effects of MICS1 overexpression should be evaluated.
MICS1 in Ca2+-mediated cell death should be assessed by manipulating MCU and considering the role of ATP synthase as a key PTP complex component (PMID: 34880425).
The authors should clarify how MICS1 downregulation could impact LETM1 levels and the alteration in fusion/fission machinery. How could MICS1-loss affect OPA1?

Referee #3:

Mitochondrial calcium homeostasis is critical to regulate cellular metabolism and mitochondrial signalling. In this study, Austin et al, using a LETM1 interactome study identified the protein MICS1/TMBIM5 as the long sought after Ca2+/H+ exchanger of the inner mitochondrial membrane.

The manuscript is written in a clear and concise well-readable manner. The experimental parts exploring the Ca2+ transport properties of MICS1/TMBIM5 are extensive and well performed, while the parts assessing the interaction between LETM1 and MICS1/TMBIM5 that forms the starting point of this study fall a bit short of what I would expect.

Points to address:

a/ Fig. 1D: I miss the immunoblot against PHB as negative control; what is the percentage of input to judge for the efficiency of interaction? This experiment should have also been performed with the LETM1 and MICS1 knockout cells.

b/ There is no further characterization of the LETM1-MICS1 interaction which I deem important.
b1/ What are the determinants of interaction between MICS1 and LETM1
b2/ Which parts of the protein are necessary for interaction
b3/ Is the interaction static or dynamic
b4/ what is the stoichiometry of components in the complex
b5/ a BN-PAGE analysis of LETM1 or MICS1 KD/KO cells should reveal whether complex size changes compared to the WT

c/ Fig. 2A/3E: LETM1 and MICS1 levels appear to be dependent on each other; is stability or import impaired? Does LETM1 knockdown result in depletion of MICS1? While siRNA-mediated depletion of MICS1 results in apparent depletion of LETM1 (Fig. 2A), this does not seem to be the case in Fig. 3E for the MICS1 KO. How can this discrepancy be explained?

Minor points:

a/ Molecular weight markers are missing with essentially all immunoblots and should be added.

b/ P4, line 72: the authors should mention that MICS1 is also TMBIM5

c/ P5, line 101: "Thus, the method was sufficiently robust to cover approximately 75% of a mitochondrial core interactome" - This is imprecise as the authors only tested for MCU interactors and not all mitochondrial proteins

Dear Dr. Senyilmaz Tiebe

Thank you very much for your interest in our submission, as well as the handling and evaluation of our manuscript. We appreciate the opportunity to submit a revised version of the manuscript and have added several new experiments, which with the revised text address all major comments of the reviewers that have direct relevance to our conclusions.

Regarding the request to address all point raised by the reviewers, please allow us to remark that we found the suggestion of reviewer #2 to inspect the link to MCU and cell death beyond the scope of this work. In this study, we present a new approach for organellar interactome analysis, introduce MICS1 as interaction partner of LETM1, and our major focus is the functional characterization of MICS1 in mitochondrial Ca2+/H+ exchange. Also, the requests of reviewer #3 are absolutely meaningful, however, to mechanistically unveil under which conditions MICS1 and LETM1 interact and which parts of the proteins are engaged in this interaction is the topic of a more detailed follow up manuscript. Therefore, experimentally answering these questions at this state would provide an appendix with half answered points and this did not feel right. We believe that the data provided in the revised version by following the constructive suggestions of all three reviewers strongly support our conclusion.

It was mentioned in your email that authors should contact the editor upon publication of any related work during the review process. We noticed that Life Science Alliance has recently published a paper by Zhang et al. on the TMBIM5 topic, but it shows different results and therefore probably should not compete with the conceptual advance of our manuscript.
Also, Thomas Langer recently informed me that he had submitted a paper on TMBIM5 to the EMBO Journal that has now been accepted for publication. However, we have not seen that the paper is publicly available. For this reason, we hope that our data will be an interesting and good match to Langer's paper, and that our revision will meet the expectations of the reviewers and the editor, such that perhaps both stories on TMBIM5 could appear at a favorable time interval from each other.

Please find our revised manuscript, which addresses as discussed the major concerns and minor points. We have used a different font color to indicate changes (those to make the text clearer and for changed figure numbers) and highlighted the text for the new experiments. Please find also below a detailed point-by-point response to each of the reviewers' comments

Sincerely yours,

Karin Nowikovsky

Referee #1:

In mammalian cells, mitochondria play a central role in calcium homeostasis. Despite the important role of mitochondria in this context, the components that mediate calcium transport across mitochondrial membranes are only poorly understood. Even the main calcium transporter, the MCU, was only discovered rather recently.
In this study, the authors characterize the inner membrane protein MICS1/TMBIM5 and identify it as a so far uncharacterized calcium/proton antiporter that is associated with the well-known inner membrane protein LETM1. In particular, the characterization of the transport activity of TMBIM5 is very convincing and the authors provide evidence that TMBIM5 mediates natrium-independent calcium efflux from mitochondria under physiological conditions. Given the novelty of TMBIM5 and the comprehensive analysis of the transport activity of TMBIM5 this study will be of interest for many readers in the future, even though several biochemical questions will have to be addressed in the future in follow-up studies. Most experiments are well controlled and the manuscript is well written and clear. The in vitro reconstitution shown in the last figure is particularly interesting, however, seems a bit immature. This aspect needs to be further improved.

We thank the reviewer for their supportive evaluation of our manuscript and encouraging suggestion to improve the in vitro reconstitution data.

Specific points
1. Fig. 1D. The pulldown of LETM1 efficiently co-isolated MICS1/TMBIM5, resulting in comparable signals. However, the pulldown of MICS1/TMBIM5 does barely isolate any LETM1. This is unexpected and should be repeated. Thereby, splicing of the gels should be avoided.

We thank the reviewer for bringing to our attention that we indeed obtained a weak signal for the detection of LETM1 in the MICS1 pulldown. We repeated this experiment several times and have obtained slightly improved signals. However, our continued results indicate that there is a weaker signal with the reverse CoIP, presumably with the MICS1 epitope being less accessible or effective at precipitating the bait protein, resulting in less LETM1 signal in the corresponding immunoblot. We have replaced the spliced gels.

2. Fig. S1D. The authors speculate about a LETM1-MICS complex of 400 kDa that is detectable with antibodies in BN-PAGE experiments. Since they successfully established MICS KO cells, it should be straightforward to test whether the LETM1 complex is shifted in these mutant cell lines. If the complex indeed consists of both proteins, a change in size or abundance would be expected.

This is a very good point. Following this advice, we performed BN-PAGE experiments to observe changes in MICS1 or LETM1 complexes when the expression of one or the other interactor was changed. Knockdown of LETM1 was reflected in a decrease in LETM1 in the 400 kDa complex, while bands for MICS1 increased at ~400 kDa but decreased at ~700 kDa. When we examined LETM1 in MICS1KO, we detected markedly less intense bands at ~400 kDa. Moreover, we observed a subtle shift of this attenuated band. These results are now shown in Fig 1D, right panel.

3. The proteomics experiments by which MICS1/TMBIM5 was identified are not clearly described and the data are not included in the supplements. The data set is basically limited to the supplemental figure S1c. The authors should improve this and provide the entire ms data set as excel file.

We apologize for the lack of clarity in the initial submission and also the lack of an additional Excel file with the entire MS dataset. We have now included detailed information on the experimental methodology in the Method section and an Excel file in the Supplementary section. In addition, all data are publicly available for download from PRIDE as indicated by the links at the end of the Method section.

4. There are several studies on MICS1/TMBIM5 that were recently published or are uploaded to BioRXiv. The authors here should also refer to their protein as TMBIM5 (rather than MICS) to avoid confusion.

Since OKA's group describes this protein in a pioneering paper under the name MICS1 (doi: 10.1091/mbc.E07-12-1205) and there are several other studies on the interaction of MICS1 with CHCHD2, we had chosen the name MICS1. However, we agree with the reviewer and at first occurrence present the protein as TMBIM5/MICS1 to reflect Oka's work and then change completely to TMBIM5 for the remainder of the manuscript.

5. The authors propose that in vivo, MICS1/TMBIM5 and LETM1 form a complex and cooperate. However, their reconstitution experiment shown in Fig. 8 only shows MICS1/TMBIM5 with LETM1. The authors should make use of this very interesting in vitro approach and co-reconstitute LETM1 into the same vesicles. Does LETM1 alter the calcium-transport properties of MICS1/TMBIM5

We thank the reviewer for highlighting this point that might have relevance for confirming the data from intact mitochondria experiments, i.e., no effect of LETM1 on the CHE activity of TMBIM5. However, it has to be stressed that co-reconstitution experiments would need setting up protocols for LETM1 over-expression, purification and reconstitution and then co-reconstitution with MICS1. This is a challenging model that has been rarely employed for transporters (see refs Indiveri et al JBC 1987 and

Schenck et al Nature Neuroscience 2009) due to the difficult goal of ensuring the actual presence of two different proteins in the same vesicle. Indeed, the detergent removal protocol employed in this manuscript is characterized by a relatively high phospholipid/protein ratio; this, on the one hand guaranteed favorable conditions for the proper insertion of functional MICS1 into liposomes. On the other hand, the high phospholipid/protein ratio, is unfavorable for inserting both MICS1 and LETM1 in the same vesicles. As a consequence, the addition of LETM1 to the experiment would result in co-reconstitution of only a very small, if any, percentage of vesicles containing both proteins. This experimental setup would make it difficult to discriminate lack of effect due to no actual interference between the two proteins from lack of effect due to incorporation in different vesicles. Therefore, considering that the in vivo data included in this manuscript already demonstrated that LETM1 is not required for the CHE activity mediated by MICS1, we decided not to further investigate this feature with a complex and not unequivocal co-reconstitution experiment.

6. The data of the reconstitution experiment are very noisy and look like single-run experiments. The authors should put more emphasis on this interesting reconstitution system, add for example nigericin and elucidate the pH-dependence of MICS1/TMBIM5-mediated calcium transport more comprehensively. This would make this study certainly more attractive.

We acknowledge that data are noisy. Thus, we have performed new experiments with better defined traces (see new Figure 7). Following the reviewer's suggestions, we have better investigated the pH dependence by creating a transient pH gradient (delta pH) by acidifying or alkalinizing the external vesicles environment (see new figure 7G). The traces recorded under delta pH conditions were consistent with the existence of a calcium/proton exchange occurring in MICS1-reconstituted liposomes. Moreover, we have also tested the effect of nigericin in the presence of external potassium, which should increase the internal pH. Under this condition, a lower calcium uptake was detected.

Minor points
6. The presentation of the y axis in Fig. 5J is odd. The authors should avoid the rescaling here.

We have rescaled the y axis.

7. The sizes standards should be added to Fig. 8B.

done

Referee #2:

The paper by Austin and colleagues claims a very strong message in the Ca2+ field, reporting the molecular identification of the H+/ Ca2+ antiporter. The same group previously reported that LETM1, identified as the mitochondrial CHE, mediates K+/H+ antiporter. Here, they defined the interactome of LETM1 and identified MICS1 as the mitochondrial CHE. Notably, a pre-print article by Langer's group (https://www.biorxiv.org/content/10.1101/2021.12.12.470907v1.full.pdf) almost confirmed the results presented here. However, other findings are required to support MICS1 as the CHE.

We thank the reviewer for their positive evaluation and constructive comments

Mitochondrial Ca2+ measurements should be performed in intact cells, using specific Ca2+ indicators. Moreover, cytosolic Ca2+ transients following mitochondrial Ca2+ efflux should differ between WT e KO cells.

We thank the reviewer for this valuable suggestion. To detect Ca2+ in intact cells, we conducted the experiment with HEK293 cells using 4mtD3cpv Cameleon to record mitochondrial and Fura 2 for cytosolic Ca2+. The cytosolic Ca2+ recording confirmed that the cells responded to agonist. However, the combined measurements indicated that the Cameleon signal bleeds through the fura-2 channel. In contrast, the converse was not the case and Fura-2 did not contaminate the Cameleon signals. Thus, since we cannot reliably quantify the cytosolic data, we prefer not to include them. Mitochondrial Ca2+ data in intact cells are shown in the new Figure 5 and described in the revised Result, Method and Legend section. We are confident that the results showing a significant reduction of Mito-Ca2+ when MICS1 is ablated support our main findings.

Key experiments should be performed in other cell types.

We have included BN using HEK293 and HeLa cells and repeated Ca2+ uptake release assays in permeabilised HeLa cells. The results were comparable and we noticed on this occasion that HeLa mitochondria seemed to buffer Ca2+ more strongly then HEK293 mitochondria when thapsigargin in not added, or express less TMBIM5 than HEK293 cells. As can be seen in EV3, wild-type mitochondrial responses to Ca2+ at a 10 µM Ca2+ pulse were lower than in HEK293. This is why we then switched to a 20 µM Ca2+ pulse in HeLa cells to reach a comparable amplitude as obtained in HEK293 with a 10 µM Ca2+ pulse.

Effects of MICS1 overexpression should be evaluated.

Although overexpression can have many secondary effects, it is true that functional characterisation is ideally strengthened when protein activity and protein content move proportionally. Therefore, we were very happy to implement the suggestion to study MICS1 overexpression. We performed parallel experiments with aliquoted cells for the Ca2+ uptake-release experiment and for the WB to monitor MICS1 levels in each flux experiment (shown below). Despite several attempts, overexpression as determined by WB could not be confirmed. Alas, in agreement with the work of Oka et al., (doi: 10.1091/mbc.E07-12-1205), which reported that MICS1 overexpression depolarizes the membrane potential and causes vacuolated mitochondria and cytotoxicity, we experienced that overexpression of MICS1 is difficult to achieve, probably because the cells try to suppress the negative effects of overexpression, resultantly fluxes were more or less comparable to those of WT. Hence, the

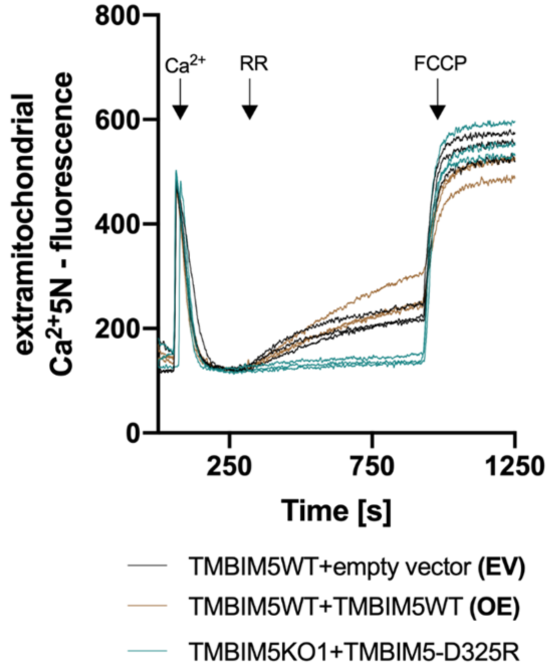

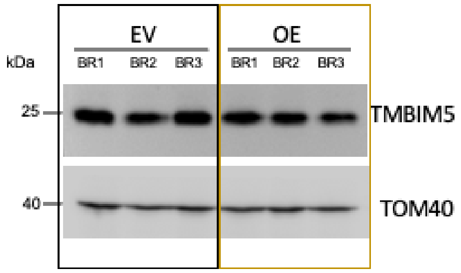

protein concentration was not increased, so that a correspondingly increased Ca2+ release was not achieved.

Finally, although serum deprivation has been shown to increase the expression of MICS1 by Oka et al., this experimental setup is not a well-controlled one, as it would be difficult to separate the effect of serum deprivation on other transporters from the effect increasing MICS1 expression by serum deprivation has on the cell.

MICS1 in Ca2+-mediated cell death should be assessed by manipulating MCU and considering the role of ATP synthase as a key PTP complex component (PMID: 34880425).

It will be important to understand the involvement of MICS1 in Ca2+ mediated cell death in the context of other important regulators of mitochondrial Ca2+ homeostasis. In this work, we focused on the molecular identification of mitochondrial CHE, which we believe is an important step in the field of mitochondrial Ca2+. Of course, we agree that this is only a first step, while raising many more exciting questions. However, we do not believe that engagement in this direction would add evidence supporting the results that MICS1 is the mitochondrial CHE presented in this manuscript.

The authors should clarify how MICS1 downregulation could impact LETM1 levels and the alteration in fusion/fission machinery. How could MICS1-loss affect OPA1?

Based on our immunoblots showing decreased levels of LETM1 (EV1 and Fig 2E_F) and LETM1-containing high molecular complexes when TMBIM5 (Fig 1 D, right panel) was ablated, and qPCR tests routinely conducted in the lab which indicated that LETM1 mRNA did not change in MICS1KO post-translational modifications for proteolytic degradation could be suggested as to how MICS1 downregulation impacts LETM1 levels. Using the proteasome inhibitor MG-123, we compared in a single experiment (shown below) how much more LETM1 was degraded in MICS1WT compared to MICS1KO and found that it was indeed more. (The qPCR data and the Fig below are shown now in Appendix Figure S7).

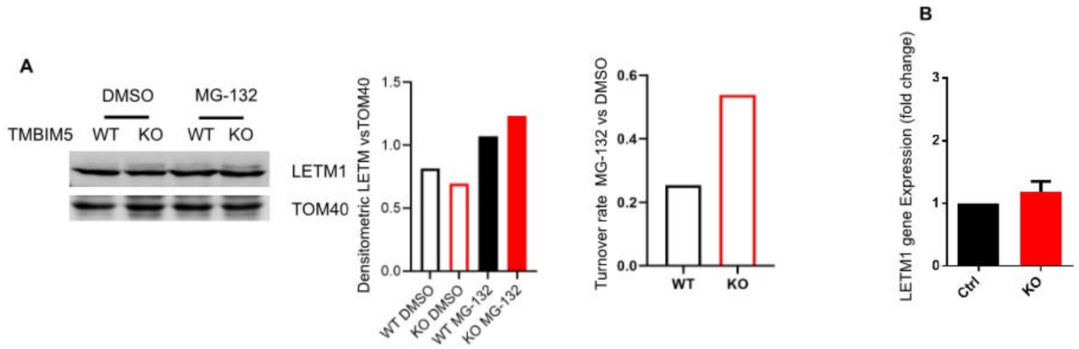

As mentioned in the pre-pint of Patron et al. (https://doi.org/10.1101/2021.12.12.470907) published during revision of this manuscript, TMBIM5 binds to and inhibit m-AAA proteases in wildtype cells, raising the question whether higher protease activity, which was reported TMBIM5KO, initiates LETM1 degradation.

It is premature to answer to the question related to OPA1. The link between OPA1 and MICS1 is now discussed (p17 line 365-369): Consistent with the role of OPA1 in cristae architecture, its interaction with the ATP synthase, the feedback loop between cristae structures and ATP synthase dimerisation (Quintana-Cabrera et al, 2018) and the fact that the PTP is formed by ATP dimers (Carrer et al, 2021), OPA1 changes under TMBIM5 ablation may explain an increased predisposition to cell death in TMBIM5KO cells exposed to Tg. However, regarding how the loss of MICS1 affects OPA1, here we can speculate that it is through its proposed function by Oka et al in "controlling the invagination of inner membranes".

Referee #3:

Mitochondrial calcium homeostasis is critical to regulate cellular metabolism and mitochondrial signalling. In this study, Austin et al, using a LETM1 interactome study identified the protein MICS1/TMBIM5 as the long sought after Ca2+/H+ exchanger of the inner mitochondrial membrane.

The manuscript is written in a clear and concise well-readable manner. The experimental parts exploring the Ca2+ transport properties of MICS1/TMBIM5 are extensive and well performed, while the parts assessing the interaction between LETM1 and MICS1/TMBIM5 that forms the starting point of this study fall a bit short of what I would expect.

We thank the reviewer for their positive evaluation of our work on MICS1 in Ca2+ transport which is a major focus of this work, and their valuable comment on the protein interaction. We agree with the reviewer that this part falls a little short, as a comprehensive analysis of this interaction that addresses the suggestions mentioned by the reviewer is the subject of a separate paper. We have included new BN experiments. We believe that the lack of an extensive interaction study does not diminish the value of our new findings.

Points to address:

a/ Fig. 1D: I miss the immunoblot against PHB as negative control; what is the percentage of input to judge for the efficiency of interaction? This experiment should have also been performed with the LETM1 and MICS1 knockout cells.

We apologize for the missing control. We repeated the experiment and included Prohibitin, which is shown in Fig 1D left. The percentage of input is 10%, as now specified in the manuscript, p29 line: 651. We are now providing a negative control using MICS1 KO.

*b/ There is no further characterization of the LETM1-MICS1 interaction which I deem importan*t.

We appreciate the thoughts of the reviewer as to the importance of the interaction and also share this curiosity, however, the scope of this work is too wide for this manuscript. We are exploring this line of investigation and will likely need to seek new and more extensive funding for a full characterisation to be published. The fact remains that a thorough interaction study as outlined in b1-b4 is outside the scope of this manuscript and will likely need to involve significant cloning and experimentation, which are not achievable in the timeframe of this revision. We want to ensure we include all significant mutations, and the consideration of different physiological status and other interaction partners identified to build a complete mechanistic view of the function of the interaction in our future study.

b1/ What are the determinants of interaction between MICS1 and LETM1

As said above, it is a pertinent question. At the moment we don't know whether the proteins interact to regulate K+, Ca2+, both, or cristae invaginations.

b2/ Which parts of the protein are necessary for interaction

This is an ongoing study in our lab that will require longer term elaboration

b3/ Is the interaction static or dynamic

This point though important will also require further experimentation that will be the focus of another manuscript. We do not wish to distract from the principal point of identifying the mitochondrial CHE at this time. The inclusion of these experiments will likely delay the manuscript and would significantly hold the study on MICS1-mediated Ca2+ transport from being publicly available.

b4/ what is the stoichiometry of components in the complex

We observed a small shift of the complex containing LETM1 when MICS1 is absent and are considering intensifying research to determine the stoichiometry of components in the complex. This again will require longer-term investigation.

b5/ a BN-PAGE analysis of LETM1 or MICS1 KD/KO cells should reveal whether complex size changes compared to the WT

This point has been addressed in the comments to reviewer 1, and we thank the reviewer for making these insightful comments. Briefly, *we found that knockdown of LETM1 was reflected in a decrease in LETM1 in the 400 kDa complex, while bands for MICS1 increased at ~400 kDa but decreased at ~700 kDa. When we examined LETM1 in MICS1KO, we detected markedly less intense bands at~ 400 kDa. The LETM1 containing*

high molecular complex (~700 kDa) decreased in mass when MICS1 was ablated. The figures are now included in Figure 1D right panel.

c/ Fig. 2A/3E: LETM1 and MICS1 levels appear to be dependent on each other; is stability or import impaired?

We did not test whether the import of Letm1 is impaired. Reviewer 2 asked a similar question. We provided a preliminary assay and a response which we include here as well.

“Based on our immunoblots showing decreased levels of LETM1 (EV1 and Fig 2E_F) and LETM1-containing high molecular complexes when TMBIM5 (Fig 1 D, right panel) was ablated, and qPCR tests routinely conducted in the lab which indicated that LETM1 mRNA did not change in MICS1KO, post-translational modifications for proteolytic degradation could be suggested as to how MICS1 downregulation impacts LETM1 levels. Using the proteasome inhibitor MG-123, we compared in a single experiment (shown below) how much more LETM1 was degraded in MICS1WT compared to MICS1KO and found that it was indeed more. (The qPCR data and the Fig below are shown now in Appendix Figure S7).

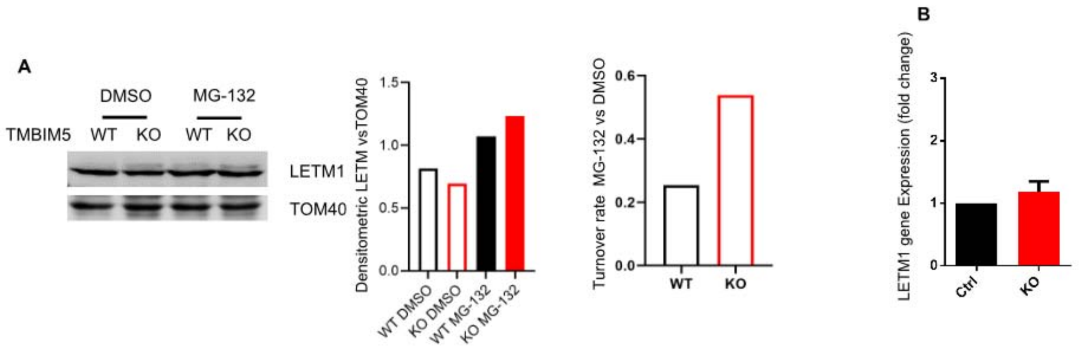

As mentioned in the pre-pint of Patron et al. (https://doi.org/10.1101/2021.12.12.470907) published during revision of this manuscript, TMBIM5 binds to and inhibit m-AAA proteases in wildtype cells, raising the question whether higher protease activity, which was reported TMBIM5KO, initiates LETM1 degradation.”

Does LETM1 knockdown result in depletion of MICS1? No, LETM11 decrease did not deplete MICS1.

While siRNA-mediated depletion of MICS1 results in apparent depletion of LETM1 (Fig. 2A), this does not seem to be the case in Fig. 3E for the MICS1 KO. How can this

discrepancy be explained?

We have clarified the loading control for the LETM1 in the blot to be HSP60, directly beneath the LETM1 immunoblot. When taking the densitometry in the Fig 2F into account there is significantly reduced LETM1, this is in alignment. with the MICS1 KD experiments shown in Figure EV1. Although the immunoblot for LETM1 is not drastically reduced compared to the loading control, there is a reduction in protein content.

Minor points:

a/ Molecular weight markers are missing with essentially all immunoblots and should be added.

done

b/ P4, line 72: the authors should mention that MICS1 is also TMBIM5

As it was also requested by Reviewer #1 to use TMBIM5 rather than MICS1, we switched to TMBIM5/MICS1 and eventually to TMBIM5

c/ P5, line 101: "Thus, the method was sufficiently robust to cover approximately 75% of a mitochondrial core interactome" - This is imprecise as the authors only tested for MCU interactors and not all mitochondrial proteins

We apologize that it was not correctly worded. We rephrased to: Thus, the method was sufficiently robust to cover approximately 75% of the MCU mitochondrial core interactome and therefore likely detect other mitochondrial interactomes with similar accuracy. (See p5 line94)

1st Revision - Editorial Decision 18th Aug 2022

Dear Dr. Nowikovsky

Thank you for the submission of your revised manuscript to EMBO reports. We have now received the full set of referee reports that is copied below.

As you will see, referee #1 and #3 are satisfied with the revision and support publication in EMBO reports, while referee #2 raises a number of remaining concerns and notes discrepancies with data published by Zhang et al. We note that meanwhile two studies were published that reported on the identification of TMBIM5 as mitochondrial Ca2+/H+ exchanger. Please discuss your data and potential similarities and discrepancies with the data reported in Zhang et al and Patron et al.
Further experiments using MEF lines will not be required for publication here, but please discuss potential limitations in your manuscript. Finally, I agree with the concern from referee 2 that a difference in LETM1 levels upon TMBIM5 KO and MG132 treatment is not visible in the Western blot supplied in Appendix Fig. S7. Please either support this statement with further data or acknowledge this concern clearly in the manuscript.

From the editorial side, there are also a few things that we need before we can proceed with the official acceptance of your study.

1) Please update the 'Conflict of interest' paragraph to our new 'Disclosure and competing interests statement'. For more information see
https://www.embopress.org/page/journal/14693178/authorguide#conflictsofinterest

2) Regarding the Author Contributions, we now use CRediT to specify the contributions of each author in the journal submission system. CRediT replaces the author contribution section in the manuscript. Please provide more detailed and complete descriptions of author contributions in our online submission system. See also our guide to authors
https://www.embopress.org/page/journal/14693178/authorguide#authorshipguidelines.
3) References: please list only the first 10 authors followed by et al.

4) Please move the figure legends to after the References.

5) Figure callouts
- Fig 2B, 4F, 5A&B, EV4A-F, Appendix Fig S1C&D, S7B and S8A callouts are missing.
- Fig EV4 is called out before EV3. Appendix Figs S4 and S6 are called out before S3. Please rearrange these figures so that they can be called out in a numerical/alphabetical order.
- Please add a callout to the Dataset (see next point).
- There are callouts to Fig EV5I-J but these panels don't exist.

6) Dataset: Please call it Dataset EV1 and add this name and a legend to a separate tab of the .xls file.

7) Appendix: please add page numbers to the table of content and move all methods and reagents (except the antibody table) to the main manuscript methods section (please update the Author checklist info on this). The Appendix is not typeset, therefore please remove the green highlights.

8) Source data: Please provide the data as one file/folder per figure (also the EV and Appendix source data). Then all can be uploaded in one ZIP file.

8a) Fig 1D seems to have a typo: TMNIM5 instead of TMBIM5. The source data for PHB and LETM1 for the right-most panel appear not to match well. For LETM1, has the band been compressed? For PHB, was a different exposure chosen? The source data label for IB:MICS, left panel is wrong. The third lane should read LETM1.

9) If you re-use and analyse public datasets, it is possible to cite these datasets as Data reference in addition to the primary paper. It appears the Mellacheruvu et al 2013, could be a data reference. See also our guide to authors
<https://www.embopress.org/page/journal/14693178/authorguide#referencesformat>.

10) Figure EV1: please add more space between the two blots to clearly show that these are two separate membranes.

11) Appendix figure legends
- S2B should refer to bands in (A) instead of C?
- S2D: please specify bars and error bars
- S3C: please specify whether n refers to technical or biological replicates. Did you perform the scr control only once? Please define bars and error bars and the statistical test used.
- S4: please define bars and error bars and the nature of the replicates (technical, biological)
- S6: please define the abbreviations (EV, OE), add information on the number of replicates and their nature and define bars and

error bars.
- S7B: please define bars and error bars and the number and nature of the replicates (technical, biological)

12) I attach to this email a related manuscript file with comments by our data editors. Please address all comments and upload a revised file with tracked changes with your final manuscript submission.

13) Finally, EMBO reports papers are accompanied online by A) a short (1-2 sentences) summary of the findings and their significance, B) 2-3 bullet points highlighting key results and C) a synopsis image that is 550x200-600 pixels large (width x height) in .png format. You can either show a model or key data in the synopsis image. Please note that the size is rather small and that text needs to be readable at the final size. Please send us this information along with the revised manuscript.

We look forward to seeing a final version of your manuscript as soon as possible.

With kind regards,

Martina Rembold, PhD
Senior Editor
EMBO reports

****

Referee #1:

The authors satisfactorily addressed all points raised on the previous submission. I fully support publication of this exciting study in its present form.

Referee #2:

The authors only partially dispelled my concerns. I appreciated that key experiments have now been ruled out in two cell lines (Hek293 and HeLa), but primary cells also (i.e., MEFs) should be tested to confirm that TMBIM5 is the CHE. Moreover, experiments in intact cells should be performed in more than one cell line.
The authors state that "overexpression of MICS1/ TMBIM5 is difficult to achieve", citing the work of Oka et al. However, in the recent paper by Zhang et al. (https://doi.org/10.26508/lsa.202201478), TMBIM5 has been successfully overexpressed. In the cover letter, it has been stated that "We noticed that Life Science Alliance has recently published a paper by Zhang et al. on the TMBIM5 topic, but it shows different results and therefore probably should not compete with the conceptual advance of our manuscript". However, in truth, such work presents key findings that compete with the data reported here, thus requiring a thorough evaluation of the role of TMBIM5. Some data by Zhang et al. are in agreement (TMBIM5 deficiency attenuates potassium-proton exchange activity). In contrast, others appear conflictual, such as i) TMBIM5 overexpression increases mitochondrial Ca2+ (the opposite of what I expected), and ii) TMBIM5 does not associate with LETM1 in a macromolecular complex. Thus, other experiments are required to account for such divergent results.
Lastly, looking at appendix figure S7, I cannot detect a difference in LETM1 levels in TMBIM5 KO cells compared to WT and after MG132 addiction. About the role of TMBIM5 in mPTP opening, I think other experiments are required to prove that these effects are strictly related to increased matrix Ca2+ load.

Referee #3:

The authors addressed all my concerns although many of them not experimentally. I can however fully understand that these additional experiments - also in the light of competing publications - are beyond the scope of this nice study. I thus support publication

Dear Dr. Rembold

I would like to thank you very much for the supportive decision letter and apologize for the length of time it took us to finalize our manuscript.

We have carefully read the new work by Patron et al., and Zhang et al., and have included a section in the discussion that briefly and objectively addresses similarities and discrepancies.
We do agree that the difference in restored LETM1 levels after proteasomal inhibition between TMBIM5 wild type (WT) and knockout (KO) is not at all impressive. We have reworded the text and labeling in the Appendix Figure (now S8B, right panel) to clarify this point. We also note that a difference in the turnover rate becomes more apparent when comparing LETM1 levels before and after proteasomal inhibition in TMBIM5KO versus TMBIM5WT. We think that the effect of TMBIM5 on LETM1 levels is more complicated than can be resolved within the scope of this study. Indeed, there is a larger decrease of LETM1 protein levels in TMBIM5KD (Fig. EV2) than in TMBIMKO, suggesting that a futile compensatory mechanism may occur in the latter to attempt restoring KHE.
We greatly appreciate your understanding not having to start a completely new study from the scratch generating TMBIM5KOs in MEFs after having provided a second cell line. We are happy to discuss the limitation of the study. Cell lines and cell-free systems are indeed best suited for a very detailed study of TMBIM5-mediated Ca^{2+}/H^{+} exchange using a number of complementary approaches, but not for understanding its tissue-specific significance. However, future studies of this tissue-specific significance would not be possible without first establishing TMBIM5 as a mitochondrial CHE.

Please find our updated manuscript in which all other changes indicated in your decision letter and by the data editor have been thoroughly made. We have marked sections that have been moved with a different font color and kept the tracking mode for the edits.
Please find also below a detailed point-to-point response to general editorial comments that were listed. We have included a graphic abstract and brief summary and bullet points highlighting key findings and significance.

We look forward to hearing from you.
Sincerely yours,

Karin Nowikovsky

Point to Point response to editorial comments.

From the editorial side, there are also a few things that we need before we can proceed with the official acceptance of your study.

We thank you for the editorial support and time taken to help us meet the required standard of EMBO Reports and have addressed all points.

1) Please update the 'Conflict of interest' paragraph to our new 'Disclosure and competing interests statement'. For more information see https://www.embopress.org/page/journal/14693178/authorguide#conflictsofinterest
Done

2) Regarding the Author Contributions, we now use CRediT to specify the contributions of each author in the journal submission system. CRediT replaces the author contribution section in the manuscript. Please provide more detailed and complete descriptions of author contributions in our online submission system. See also our guide to authors https://www.embopress.org/page/journal/14693178/authorguide#authorshipguidelines
Done

3) References: please list only the first 10 authors followed by et al.
Done

4) Please move the figure legends to after the References.
Done, highlighted in blue color

5) Figure callouts
- Fig 2B, 4F, 5A&B, EV4A-F, Appendix Fig S1C&D, S7B and S8A callouts are missing.
- Fig EV4 is called out before EV3. Appendix Figs S4 and S6 are called out before S3. Please rearrange these figures so that they can be called out in a numerical/alphabetical order.
We apologize for the callouts that were missing or not in numerical/alphabetical order. Line 135: callout for Fig 2B
Line 205: callout Fig 5A&B
Due to the recommended rearrangement, EV4 is now EV3 and former EV3 is now EV4
Line 178: callout EV3
Line 191: EV4 (previous EV3)
Lines 102, 111, 297, 341: callout for Appendix Fig 1C&D, S7B and S8A, respectively
Lines 173 and 1075: Appendix Fig S3 (previous S4)
Line 194: Appendix Fig S4 (previous S6)
Line 217: Appendix Fig S5 (previous S3)

- Please add a callout to the Dataset (see next point).
Line 97: Dataset EV1
- There are callouts to Fig EV5I-J but these panels don't exist.
Thank you for noticing and apologies for having overlooked it;
Fig EV5I-J is now Fig EV3E-F

6) Dataset: Please call it Dataset EV1 and add this name and a legend to a separate tab of the .xls file.

7) Appendix: please add page numbers to the table of content and move all methods and reagents (except the antibody table) to the main manuscript methods section (please update the Author checklist info on this). The Appendix is not typeset, therefore please remove the green highlights.
Done

8) Source data: Please provide the data as one file/folder per figure (also the EV and Appendix source data). Then all can be uploaded in one ZIP file.
Done, uploaded as one Zip File for main Figs and one Zip file for EV figs and Appendix Figs.

8a) Fig 1D seems to have a typo: TMNIM5 instead of TMBIM5. The source data for PHB and LETM1 for the right-most panel appear not to match well. For LETM1, has the band been compressed? For PHB, was a different exposure chosen? The source data label for IB:MICS, left panel is wrong. The third lane should read LETM1.
Thank you for noticing the typo, which we have now corrected.
As for the data source, the LETM1 band was not compressed. In error, we had uploaded the raw blot directly from the instrument (Chem Studio) instead of exporting it with Image Lab like the other blots. We apologize for the inadvertence. We now display below the previous blot data as well as the correctly exported one, also showing the overlay with the molecular marker. For the data source, we propose replacing the previous version with the correctly exported one to avoid further confusion, as in the current updated version.

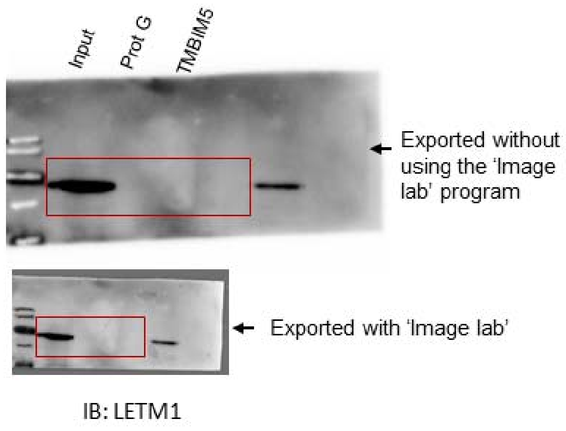

9) If you re-use and analyse public datasets, it is possible to cite these datasets as Data reference in addition to the primary paper. It appears the Mellacheruvu et al 2013, could be a data reference. See also our guide to authors <https://www.embopress.org/page/journal/14693178/authorguide#referencesformat>:.
We inserted the data reference

10) Figure EV1: please add more space between the two blots to clearly show that these are two separate membranes.
Done.

Dr. Karin Nowikovsky
University of Veterinary Medicine Vienna
Biomedical Sciences; Physiology and Biophysics
Building HA
Veterinaerplatz 1
Wien 1210
Austria

Dear Karin,

Thank you very much for your recent e-mail and for introducing the suggested textual changes. I am now very pleased to accept your manuscript for publication in the next available issue of EMBO reports. Thank you for your contribution to our journal.

At the end of this email I include important information about how to proceed. Please ensure that you take the time to read the information and complete and return the necessary forms to allow us to publish your manuscript as quickly as possible.

As part of the EMBO publication's Transparent Editorial Process, EMBO reports publishes online a Review Process File to accompany accepted manuscripts. As you are aware, this File will be published in conjunction with your paper and will include the referee reports, your point-by-point response and all pertinent correspondence relating to the manuscript.

If you do NOT want this File to be published, please inform the editorial office within 2 days, if you have not done so already, otherwise the File will be published by default [contact: emboreports@embo.org]. If you do opt out, the Review Process File link will point to the following statement: "No Review Process File is available with this article, as the authors have chosen not to make the review process public in this case." Please note that the author checklist will still be published even if you opt out of the transparent process.

Thank you again for your contribution to EMBO reports and congratulations on a successful publication. Please consider us again in the future for your most exciting work.

Kind regards,
Martina

Martina Rembold, PhD
Senior Editor
EMBO reports

****

THINGS TO DO NOW:

Once your article has been received by Wiley for production, the corresponding author will receive an email from Wiley's Author Services system which will ask them to log in and will present them with the appropriate license for completion.

You will receive proofs by e-mail approximately 2-3 weeks after all relevant files have been sent to our Production Office; you should return your corrections within 2 days of receiving the proofs.

Please inform us if there is likely to be any difficulty in reaching you at the above address at that time. Failure to meet our deadlines may result in a delay of publication, or publication without your corrections.

All further communications concerning your paper should quote reference number EMBOR-2022-54978V3 and be addressed to emboreports@wiley.com.

Should you be planning a Press Release on your article, please get in contact with emboreports@wiley.com as early as possible, in order to coordinate publication and release dates.

EMBO Press Author Checklist

Corresponding Author Name: Karin Nowikovsky
Journal Submitted to: EMBO Reports
Manuscript Number: EMBOR-2022-54978V2

USEFUL LINKS FOR COMPLETING THIS FORM
The EMBO Journal - Author Guidelines
EMBO Reports - Author Guidelines
Molecular Systems Biology - Author Guidelines
EMBO Molecular Medicine - Author Guidelines

Reporting Checklist for Life Science Articles (updated January 2022)
This checklist is adapted from Materials Design Analysis Reporting (MDAR) Checklist for Authors. MDAR establishes a minimum set of requirements in transparent reporting in the life sciences (see Statement of Task: 10.31222/osf.io/9sm4x). Please follow the journal's guidelines in preparing your manuscript.
Please note that a copy of this checklist will be published alongside your article.

Abridged guidelines for figures
1. Data
The data shown in figures should satisfy the following conditions:
- the data were obtained and processed according to the field's best practice and are presented to reflect the results of the experiments in an accurate and unbiased manner.
- ideally, figure panels should include only measurements that are directly comparable to each other and obtained with the same assay.
- plots include clearly labeled error bars for independent experiments and sample sizes. Unless justified, error bars should not be shown for technical replicates.
- if n<5, the individual data points from each experiment should be plotted. Any statistical test employed should be justified.
- Source Data should be included to report the data underlying figures according to the guidelines set out in the authorship guidelines on Data Presentation.

2. Captions
Each figure caption should contain the following information, for each panel where they are relevant:
- a specification of the experimental system investigated (eg cell line, species name).
- the assay(s) and method(s) used to carry out the reported observations and measurements.
- an explicit mention of the biological and chemical entity(ies) that are being measured.
- an explicit mention of the biological and chemical entity(ies) that are altered/varied/perturbed in a controlled manner.
- the exact sample size (n) for each experimental group/condition, given as a number, not a range;
- a description of the sample collection allowing the reader to understand whether the samples represent technical or biological replicates (including how many animals, litters, cultures, etc.).
- a statement of how many times the experiment shown was independently replicated in the laboratory.
- definitions of statistical methods and measures:
  - common tests, such as t-test (please specify whether paired vs. unpaired), simple χ2 tests, Wilcoxon and Mann-Whitney tests, can be unambiguously identified by name only, but more complex techniques should be described in the methods section;
  - are tests one-sided or two-sided?
  - are there adjustments for multiple comparisons?
  - exact statistical test results, e.g., P values = x but not P values < x;
  - definition of 'center values' as median or average;
  - definition of error bars as s.d. or s.e.m.

Please complete ALL of the questions below.
Select "Not Applicable" only when the requested information is not relevant for your study.

Materials

Newly Created Materials	Information included in the manuscript?	In which section is the information available? (Reagents and Tools Table, Materials and Methods, Figures, Data Availability Section)
New materials and reagents need to be available; do any restrictions apply?	Not Applicable	

Antibodies	Information included in the manuscript?	In which section is the information available? (Reagents and Tools Table, Materials and Methods, Figures, Data Availability Section)
For **antibodies** provide the following information:<br>- Commercial antibodies: RRID (if possible) or supplier name, catalogue number and or/clone number<br>- Non-commercial: RRID or citation	Yes	Appendix Table S1

DNA and RNA sequences	Information included in the manuscript?	In which section is the information available? (Reagents and Tools Table, Materials and Methods, Figures, Data Availability Section)
**Short novel DNA or RNA including primers, probes**: provide the sequences.	Yes	Materials and Methods

Cell materials	Information included in the manuscript?	In which section is the information available? (Reagents and Tools Table, Materials and Methods, Figures, Data Availability Section)
**Cell lines:** Provide species information, strain. Provide accession number in repository **OR** supplier name, catalog number, clone number, and/**OR** RRID.	Yes	Materials and Methods
**Primary cultures:** Provide species, strain, sex of origin, genetic modification status.	Not Applicable	
Report if the cell lines were recently **authenticated** (e.g., by STR profiling) and tested for mycoplasma contamination.	Yes	Materials and Methods

Experimental animals	Information included in the manuscript?	In which section is the information available? (Reagents and Tools Table, Materials and Methods, Figures, Data Availability Section)
**Laboratory animals or Model organisms:** Provide species, strain, sex, age, genetic modification status. Provide accession number in repository **OR** supplier name, catalog number, clone number, **OR** RRID.	Not Applicable	
**Animal observed in or captured from the field:** Provide species, sex, and age where possible.	Not Applicable	
Please detail **housing and husbandry conditions**.	Not Applicable	

Plants and microbes	Information included in the manuscript?	In which section is the information available? (Reagents and Tools Table, Materials and Methods, Figures, Data Availability Section)
**Plants:** provide species and strain, ecotype and cultivar where relevant, unique accession number if available, and source (including location for collected wild specimens).	Not Applicable	
**Microbes:** provide species and strain, unique accession number if available, and source.	Yes	Materials and Methods

Human research participants	Information included in the manuscript?	In which section is the information available? (Reagents and Tools Table, Materials and Methods, Figures, Data Availability Section)
If collected and within the bounds of privacy constraints report on age, sex and gender or ethnicity for all study participants.	Not Applicable	

Core facilities	Information included in the manuscript?	In which section is the information available? (Reagents and Tools Table, Materials and Methods, Figures, Data Availability Section)
If your work benefited from core facilities, was their service mentioned in the acknowledgments section?	Yes	Materials and Methods

Design

Study protocol	Information included in the manuscript?	In which section is the information available? (Reagents and Tools Table, Materials and Methods, Figures, Data Availability Section)
If study protocol has been **pre-registered, provide DOI in the manuscript**. For clinical trials, provide the trial registration number **OR** cite DOI.	Not Applicable	
Report the **clinical trial registration number** (at ClinicalTrials.gov or equivalent), where applicable.	Not Applicable	

Laboratory protocol	Information included in the manuscript?	In which section is the information available? (Reagents and Tools Table, Materials and Methods, Figures, Data Availability Section)
Provide DOI OR other citation details if **external detailed step-by-step protocols** are available.	Yes	Materials and Methods

Experimental study design and statistics	Information included in the manuscript?	In which section is the information available? (Reagents and Tools Table, Materials and Methods, Figures, Data Availability Section)
Include a statement about **sample size** estimate even if no statistical methods were used.	Yes	Materials and Methods
Were any steps taken to minimize the effects of subjective bias when allocating animals/samples to treatment (e.g. **randomization procedure**)? If yes, have they been described?	Not Applicable	
Include a statement about **blinding** even if no blinding was done.	Not Applicable	
Describe **inclusion/exclusion criteria** if samples or animals were excluded from the analysis. Were the criteria pre-established? If sample or data points were omitted from analysis, report if this was due to attrition or intentional exclusion and provide justification.	Not Applicable	
For every figure, are **statistical tests** justified as appropriate? Do the data meet the assumptions of the tests (e.g., normal distribution)? Describe any methods used to assess it. Is there an estimate of variation within each group of data? Is the variance similar between the groups that are being statistically compared?	Yes	Figure legends

Sample definition and in-laboratory replication	Information included in the manuscript?	In which section is the information available? (Reagents and Tools Table, Materials and Methods, Figures, Data Availability Section)
In the figure legends: state number of times the experiment was **replicated** in laboratory.	Yes	Figure legends
In the figure legends: define whether data describe **technical or biological replicates**.	Yes	Figure legends

Ethics

Ethics	Information included in the manuscript?	In which section is the information available? (Reagents and Tools Table, Materials and Methods, Figures, Data Availability Section)
Studies involving **human participants**: State details of **authority granting ethics approval** (IRB or equivalent committee(s), provide reference number for approval.	Not Applicable	
Studies involving **human participants**: Include a statement confirming that **informed consent** was obtained from all subjects and that the experiments conformed to the principles set out in the WMA Declaration of Helsinki and the Department of Health and Human Services Belmont Report.	Not Applicable	
Studies involving **human participants:** For publication of **patient photos**, include a statement confirming that consent to publish was obtained.	Not Applicable	
Studies involving experimental **animals**: State details of **authority granting ethics approval** (IRB or equivalent committee(s), provide reference number for approval. Include a statement of compliance with ethical regulations.	Not Applicable	
Studies involving **specimen and field samples:** State if relevant **permits** obtained, provide details of authority approving study; if none were required, explain why.	Not Applicable	

Dual Use Research of Concern (DURC)	Information included in the manuscript?	In which section is the information available? (Reagents and Tools Table, Materials and Methods, Figures, Data Availability Section)
Could your study fall under dual use research restrictions? Please check biosecurity documents and list of **select agents and toxins** (CDC): https://www.selectagents.gov/sat/list.htm	Not Applicable	
If you used a select agent, is the security level of the lab appropriate and reported in the manuscript?	Not Applicable	
If a study is subject to dual use research of concern regulations, is the name of the **authority granting approval and reference number** for the regulatory approval provided in the manuscript?	Not Applicable	

Reporting

The MDAR framework recommends adoption of discipline-specific guidelines, established and endorsed through community initiatives. Journals have their own policy about requiring specific guidelines and recommendations to complement MDAR.

Adherence to community standards	Information included in the manuscript?	In which section is the information available? (Reagents and Tools Table, Materials and Methods, Figures, Data Availability Section)
State if relevant guidelines or checklists (e.g., **ICMJE, MIBBI, ARRIVE, PRISMA**) have been followed or provided.	Not Applicable	
For **tumor marker prognostic studies,** we recommend that you follow the **REMARK** reporting guidelines (see link list at top right). See author guidelines, under 'Reporting Guidelines'. Please confirm you have followed these guidelines.	Not Applicable	
For **phase II and III randomized controlled trials**, please refer to the **CONSORT** flow diagram (see link list at top right) and submit the CONSORT checklist (see link list at top right) with your submission. See author guidelines, under 'Reporting Guidelines'. Please confirm you have submitted this list.	Not Applicable	

Data Availability

Data availability	Information included in the manuscript?	In which section is the information available? (Reagents and Tools Table, Materials and Methods, Figures, Data Availability Section)
Have **primary datasets** been deposited according to the journal's guidelines (see 'Data Deposition' section) and the respective accession numbers provided in the Data Availability Section?	Yes	Data Availability Section
Were **human clinical and genomic datasets** deposited in a public access-controlled repository in accordance to ethical obligations to the patients and to the applicable consent agreement?	Not Applicable	
Are **computational models** that are central and integral to a study available without restrictions in a machine-readable form? Were the relevant accession numbers or links provided?	Not Applicable	
If publicly available data were reused, provide the respective **data citations in the reference list**.	Yes	References
